# Multi-source temporal fusion and multi-station learning for early warning of diamondback moth infestation in *Brassica* crops

**DOI:** 10.3389/fpls.2026.1953590

**Published:** 2026-10-12

**Authors:** Dong Zhang, Jiale Wang

**Affiliations:** 1 School of Information Management, Sun Yat-sen University, Guangzhou, Guangdong, China; 2 Institute of Quality Standard and Monitoring Technology for Agro-products of Guangdong Academy of Agricultural Sciences, Guangzhou, China

**Keywords:** high-frequency meteorology, integrated pest management, interpretable deep learning, multi-station learning, pest forecasting, *Plutella xylostella*

## Abstract

**Introduction:**

Meteorological variability influences diamondback moth (DBM; *Plutella xylostella*) infestation and the resulting damage to *Brassica* crops. Accurate multi-horizon forecasting requires models that preserve short-lived meteorological exposures while integrating thermal timing, spatial dependence, and recent infestation dynamics within a transparent framework.

**Methods:**

The Temporal–Geospatial Pest Network (TG-PestNet) forecasts the weekly percentage of inspected plants classified as DBM-infested 1 to 4 weeks ahead at four Guangdong stations, from origin-time information only. It preserves hourly exposure, encodes lookback weeks on a thermal-time axis, couples stations through a wind-informed graph, and protects infestation history in a gated anchor. Evaluation used 344 synchronized weeks, 66 later test origins, 13 history-only comparators, retraining ablations, matched-width controls, and input-matched generic encoders.

**Results and discussion:**

TG-PestNet achieved the lowest observed root-mean-square error, mean absolute error, and mean absolute percentage error in all 12 station–metric combinations, with mean reductions of 5.9%–13.0%. Year-ahead forecasts outperformed persistence in all four test years. Removing the history anchor increased error by 14.3% and remained significant after multiplicity correction, demonstrating its central contribution to forecast accuracy. Meteorological information provided complementary predictive value: its occlusion increased the error by 9.3%, and retraining without it increased the macro RMSE by 3.6%. TG-PestNet also substantially outperformed the two input-matched generic encoders, whose errors were approximately one quarter higher. These results establish TG-PestNet as an accurate, reproducible and auditable framework for multi-station pest forecasting, with component-level analyses that identify the dominant contribution of recent infestation history and characterize the predictive roles of high-resolution meteorology and structured spatial information.

## 1 Introduction

Diamondback moth (DBM; *Plutella xylostella*) is a major constraint on horticultural *Brassica* and oilseed production worldwide. Rapid development, multiple generations, high dispersal capacity, and widespread insecticide resistance combine to make populations difficult to suppress once crop damage has escalated. The combined cost of crop loss and control has been estimated at several billion US dollars annually (Zalucki et al., 2012; Furlong et al., 2013). There is therefore a clear need for anticipatory rather than reactive management: a 1- to 4-week warning would support targeted scouting, biological-control preparation, and selective intervention before injury becomes severe, and reliable early warning is a key component of integrated pest management and landscape-level biological control (Gurr et al., 2018).

Temperature is central to DBM risk. It regulates development, fecundity, and survival; accumulated warmth rather than calendar time sets the pace of the life cycle; and intrinsic growth is highest at around 20 °C–25 °C, with oviposition declining above approximately 30 °C (Trudgill et al., 2005; Marchioro and Foerster, 2012; Bahar et al., 2014; Ngowi et al., 2017). Because these responses are nonlinear, 2 weeks with the same mean temperature can differ in the number of hot hours, the rate of warming, and the degree-days accumulated, and weekly averaging discards that difference.

Temperature alone does not, however, provide a complete causal account of DBM dynamics. Rainfall can dislodge eggs and young larvae (Kobori and Amano, 2003), wind affects flight and long-distance movement (Goodwin and Danthanarayana, 1984; Chapman et al., 2002; Wei et al., 2013), and weather interacts with host availability, crop stage, natural enemies, resistance, and management (Li et al., 2012, 2016a, 2016b; Gurr et al., 2018; Machekano et al., 2019). Accordingly, precipitation and wind are retained as predictors, and wind direction conditions cross-station information exchange; the learned coupling is evaluated through predictive diagnostics and retraining experiments.

There is a growing body of literature that applies statistical learning and deep neural networks to meteorology-based pest forecasting. Conventional machine learning models have been used to combine compact weather, crop, or remote-sensing inputs (Skawsang et al., 2019), recurrent architectures have represented temporal dependence in cotton pest and disease forecasting (Xiao et al., 2019; Chen et al., 2020), and graph models have introduced explicit connectivity for migratory rice pests (Zhang et al., 2023). For DBM, ALIC combined feature attention, LSTM encoding, and interaction convolution over monthly meteorological variables and pest history (Wang and Zhang, 2024), while MeteoSCOPE represented multidimensional monthly descriptors as structured feature-lag tokens (Zhang and Wang, 2026). These pest-specific developments complement generic sequence models such as LSTM (Hochreiter and Schmidhuber, 1997), temporal convolutional networks (Bai et al., 2018), N-HiTS (Challu et al., 2023), NLinear (Zeng et al., 2023), and the Transformer (Vaswani et al., 2017).

Despite these advances, two gaps remain. First, the structural choices the biology suggests—hourly rather than aggregated exposure, a temperature-dependent rather than calendar time axis, wind-conditioned rather than independent stations, and an explicit treatment of bounded, endpoint-inflated infestation history—have seldom been built into a single forecaster and then tested for whether each of them contributes. Second, the biological interpretation of fitted parameters and attention weights benefits from explicit tests of movement from initialization, cross-seed stability, and agreement with perturbation-based attribution. This study applies these checks to every biologically motivated component as a hypothesis about input representation.

To address these gaps, we propose the Temporal–Geospatial Pest Network (TG-PestNet), a direct multi-horizon architecture that jointly forecasts the weekly DBM-infested plant percentage—the percentage of inspected plants classified as DBM-infested in a station’s weekly surveillance report—at four monitoring stations in Guangdong Province, southern China, from information available at the forecast origin. It converts 8 weeks of hourly meteorology into weekly station embeddings through distributional summaries, differentiable exposure counts, and attention pooling, adds an embedding of the observed infestation history to each of those embeddings, replaces calendar distance between lookback weeks by degree-day distance in the temporal attention bias, conditions cross-station attention on a learned wind-and-distance edge bias, and combines a bounded latent-state recursion with a learnable history anchor through a logit-space gate. The reported output is a bounded point forecast. Entomological evidence guides three representational choices: the exposures preserved at hourly resolution, the six variables equipped with threshold operators, and wind-conditioned cross-station information exchange. The fitted thresholds, base temperature, and station-pair bias are evaluated as predictive operating points through stability, perturbation, and retraining analyses.

Against this background, this study set out to answer two questions. The first is whether TG-PestNet improves 1- to 4-week forecasts beyond strong history-only benchmarks and beyond generic architectures given the same inputs. The second is which of its components remain empirically supported once each is removed and the model retrained and how the contribution of meteorology varies among stations. It makes two contributions: a multi-station architecture that protects the autoregressive signal in recent infestation history through a learnable anchor and gate while preserving high-frequency meteorological exposure and an evaluation and auditing framework that is applied to the proposed model itself rather than only to competitors. Evaluation follows a strictly forward chronological design, with test labels excluded from the architecture search and from the selection of the reported configuration. The present study focuses on bounded point forecasting and component-level predictive evidence, while uncertainty quantification and mechanistic validation provide directions for subsequent work.

## 2 Materials and methods

### 2.1 Study design, surveillance data, and forecasting task

Let *t* denote a weekly forecast origin, *S* = 4 the number of monitoring stations, *L* = 8 the lookback length in completed weeks, *V* = 39 the number of hourly meteorological variables, and *H* = 4 the forecast horizons. For each origin, the model receives standardized hourly weather for the previous 8 weeks, raw temperature for degree-day calculation, the most recent eight weekly DBM-infested plant percentages, weekly mean 100-m wind components, station-pair bearings and distances, and known target-week calendar features. The observed history enters at three points: the projection described in Section 2.3.1, and the decoder initialization and history anchor described in Section 2.3.3. The model outputs bounded point forecasts of the DBM-infested plant percentage for every station at *t* + 1,…, *t* + 4 in a single forward pass; it also produces auxiliary encoder-branch ZOIB parameters used only in the training objective.

The formulation is direct rather than recursive with respect to predicted observations: the model never feeds a forecast back as an input. The four-step latent-state decoder is internal to the output head and is initialized from an endpoint-clipped version of the last observed DBM-infested plant percentage. All meteorological inputs end at *t*, so every forecast is generated exclusively from information available at its origin. The DBM-infested plant percentages are divided by 100 and modeled on [0, 1]. A full notation table is provided in **Supplementary Table 8**.

The response is the weekly percentage of inspected plants classified as infested by DBM at four vegetable-production monitoring sites in Guangdong Province, southern China: Huiyang, Shantou, Shenzhen, and Dongguan (**Table 1**). *P. xylostella* infestation observations are aligned to common weekly windows. Huiyang, Shenzhen, and Dongguan form a relatively compact cluster, whereas Shantou lies farther to the northeast. Previous Guangdong studies have shown that DBM dynamics vary with local climate, cropping pattern, natural enemies, and management (Li et al., 2012, 2016a, 2016b; Gurr et al., 2018; Machekano et al., 2019), which provides the rationale for a cross-station layer that retains the bearing and distance between sites.

**Table 1:** Study sites, the synchronized weekly calendar, and the test-period response.

Station	Coordinates (°N, °E)	Synchronized weeks	Test weeks	Mean ± SD (%)	Median (%)
Huiyang	22.98, 114.47	344	69	19.84 ± 15.46	16.00
Shantou	23.35, 116.68	344	69	19.46 ± 3.88	21.25
Shenzhen	22.55, 114.06	344	69	8.38 ± 8.06	6.00
Dongguan	23.02, 113.75	344	69	8.49 ± 5.11	7.45

The coordinates are decimal degrees. The synchronized calendar is the 344 Sunday-ended weeks from 7–13 September 2015 to the week ending 10 April 2022; the test period covers the 69 target weeks of the 66 test origins, December 14, 2020 to April 2022. The weekly DBM-infested plant percentage is reported on the original percentage scale. Exact zeros occur in one test week at Huiyang and nine at Shenzhen and in none at Shantou or Dongguan. MAPE excludes exact zeros and remains sensitive to small positive observations, especially in volatile series. Over the full calendar, Shenzhen is the most volatile series (SD 28.39 percentage points) and Shantou the most stable (SD 7.28), so a pooled error criterion would be dominated by the higher-variance stations; the primary score gives equal weight to each station-by-metric cell.

Weekly diamondback moth surveillance data were provided by the Department of Agriculture and Rural Affairs of Guangdong Province through its Crop Pest Forecast and Control Center. The records originated from the systematic-survey component of the provincial plant protection surveillance network. Field monitoring was conducted under a common operational protocol implemented in accordance with GB/T 23392.3-2009, Technical specification for the forecast of diseases and insects on cruciferous plants—Part 3: *Plutella xylostella*. The provider confirmed in writing that the same formal protocol was applied throughout 2015–2022. **Supplementary Table 11** and **Supplementary Methods SM1.1, SM1.2** document the protocol dimensions and the metadata available in the supplied extract.

At each survey round, trained personnel from the local plant protection station examined one to three representative *Brassica* fields within each station’s designated administrative monitoring area. Each field was sampled at five points, with 20 plants physically inspected at each point and therefore 100 plants inspected per field per survey round. Surveys were normally conducted every 3–5 days. The sampled fields were maintained as untreated reference fields, with no insecticides applied throughout the crop-growing period.

The provincial database recorded the response as the infested-plant rate, termed in this study as the weekly DBM-infested plant percentage and defined as the percentage of inspected plants classified as infested by DBM. Under the provider’s operational definition, a plant was classified as DBM-infested when either a DBM individual at any life stage was directly observed or characteristic DBM feeding signs were present, including early-instar window-pane feeding and the characteristic shot holes or defoliation associated with later instars.

For station *s* and reporting week *w*, the provincial reporting system pooled all contributing field-survey records according to Equation 1:

(1) $$y_{s,w}=100\times \frac{\sum_{r\in R_{s,w}}a_{r}}{\sum_{r\in R_{s,w}}n_{r}}$$

where *R*_s,w_ is the set of field-survey records contributing to the station-week report, *a*_r_ is the number of plants classified as DBM-infested, and *n*_r_ is the number of plants inspected. Each surveyed field contributed 100 inspected plants. Thus, *y*_s,w_ is a denominator-weighted percentage calculated from all plant inspections contributing to the reporting week, rather than an unweighted average of the individual survey percentages.

The cross-station layer and its joint batch require simultaneous observations at all four sites. Huiyang and Shantou records begin in 2004 and 2005, whereas Shenzhen and Dongguan records begin on September 7, 2015; the primary analysis therefore uses the common Sunday-ended calendar from the week of September 7–13, 2015 through the week ending April 10, 2022. Weeks run Monday to Sunday and are labeled by their Sunday end date. This interval contains exactly 344 synchronized weekly labels per station, retains 36% of the Huiyang archive and 39% of the Shantou archive, and yields a first admissible training origin of November 1, 2015. Protocol consistency is confirmed for 2015–2022; the supplied extract does not document the protocol for the earlier Huiyang and Shantou records, and both archive starts predate the 2009 standard (**Supplementary Methods SM1.2**). Section 3.3 quantifies the forecasting value of extending the training history before 2015.

### 2.2 Meteorological data and preprocessing

Hourly reanalysis meteorology was obtained from the HTQX meteorological data platform (https://htqx.cn/meteo), which distributes the ERA5 reanalysis produced by ECMWF and the Copernicus Climate Change Service. Each record carries a timestamp in UTC + 8 and 39 numeric variables, all of which enter the hourly encoder. Section 3.2 reports their variance characteristics. One input, weather_code, is the WMO 4677 present-weather code. In the frozen preprocessing, it was retained as a single standardized numeric implementation feature rather than being one-hot encoded or embedded; no physical meaning is assigned to the numerical spacing between codes. A model input week is retained when all 168 hourly observations are present, ensuring complete hourly blocks without imputation. The variables are organized into the seven physically coherent groups of **Supplementary Table 9**, which are also the units used by the variable-group attention. The complete file manifest with record counts and checksums, and the near-zero-variance screening of all 39 variables, are presented in **Supplementary Methods SM1.3** and **Supplementary Table 13**.

Six variables receive differentiable soft thresholds, at four thresholds each: 2-m temperature, vapor-pressure deficit, precipitation, 10-m wind speed, 10-m wind gusts, and shortwave radiation. They were selected *a priori*. For temperature, rainfall, and wind, the DBM-specific evidence is direct—temperature governs development, survival, and reproduction (Marchioro and Foerster, 2012; Bahar et al., 2014; Ngowi et al., 2017), rainfall dislodges eggs and early instars (Kobori and Amano, 2003), and wind governs flight and long-distance movement (Goodwin and Danthanarayana, 1984; Chapman et al., 2002; Wei et al., 2013). Vapor pressure deficit and shortwave radiation are included as physically motivated proxies: humidity interacts with temperature (Yamada and Kawasaki, 1983; Guo and Qin, 2010), host water deficit lowers DBM performance (Valim et al., 2016), leaf-surface humidity reflects atmospheric humidity (Pincebourde and Woods, 2012), and solar UV-B alters host attractiveness and resistance (Caputo et al., 2006; Foggo et al., 2007; McInnes et al., 2023). Their role is documented in **Supplementary Methods SM1.4**, and all learned thresholds are trained as predictive operating points without predefined stress labels.

Standardization moments were estimated from the hours of the fitting block of each stage—training hours during model selection and training-plus-validation hours for the final refit—and applied unchanged to all later data. All standardization moments are derived exclusively from fitting-block hours, with the last fitting week preceding the first test forecast origin. Raw temperature remains in degrees Celsius for degree-day calculation, and all meteorological inputs end at the forecast origin (**Supplementary Methods SM1.5**).

### 2.3 TG-PestNet architecture

TG-PestNet comprises four computational modules and an infestation-history projection (**Figure 1**): a threshold-exposure hourly encoder that transforms 168 hourly values per week into a structured weekly station embedding, a thermal-time encoder that encodes the 8-week sequence using degree-day-relative attention, wind-informed cross-station coupling that exchanges information among sites through a learned edge bias, and a latent-state decoder that advances a bounded state, forms an auxiliary encoder-branch ZOIB mean, and blends that mean with a learned history anchor to produce the final point forecast. Encoder weights are shared across stations, while station embeddings and station-horizon gates allow local adaptation. The infestation-history projection adds an embedding of the observed weekly value to each weekly station embedding, making the encoder pathway jointly meteorology- and history-informed. Each named component is evaluated as a structural design choice through the retraining ablations of Section 2.4.2.

**Figure 1:**
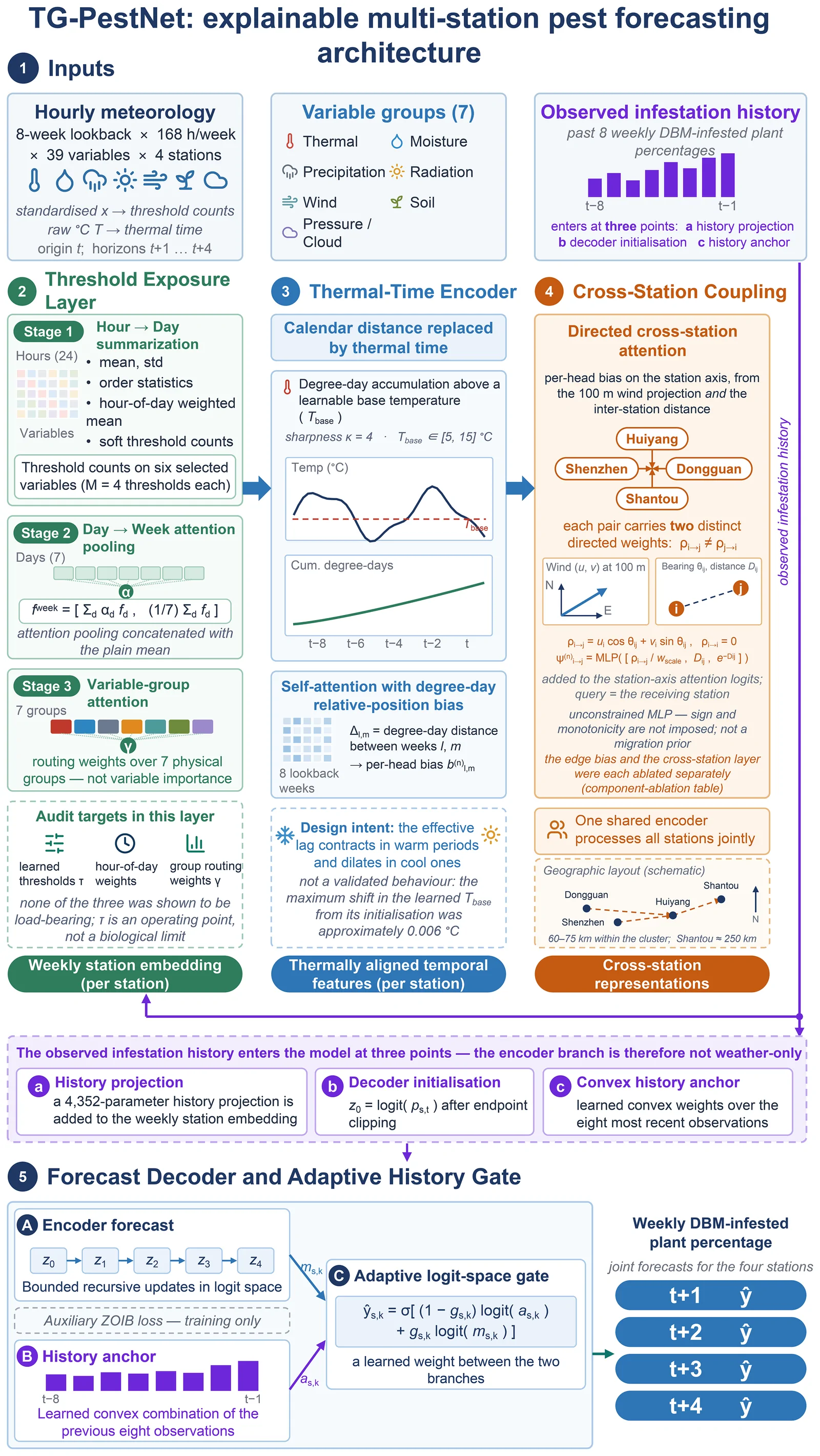
TG-PestNet architecture. Hourly meteorology and eight weeks of observed infestation history are processed by a threshold-exposure hourly encoder, with an infestation-history projection added to each weekly station embedding (Section 2.3.1); a thermal-time encoder; wind-informed cross-station coupling; and a latent-state decoder with a protected history path and auxiliary ZOIB head. Observed history enters through the weekly projection, decoder initialization and history anchor, so both forecasting branches incorporate infestation history. The final output is a bounded point forecast for horizons one to four weeks; the auxiliary ZOIB head supplies an encoder-branch training objective.

#### 2.3.1 Hourly-to-weekly representation and history projection

A station-week contains 168 hourly readings for each of 39 variables. TG-PestNet summarizes this full tensor hierarchically in three stages—hour to day, day to week, and variable group to station embedding—preserving high-frequency exposure patterns in a compact and auditable weekly representation.

For day *d* and variable *v*, the encoder computes the mean, the standard deviation, *K* = 5 order statistics, and an hour-of-day weighted mean over the 24 within-day hours indexed by *r*; the hour-of-day weights are shared within the physical group of the variable. With four exposure-count slots, identically zero for the 33 variables that carry no thresholds, each variable-day yields a 12-element summary, and the weekly per-variable vector concatenates the attention-pooled and mean-pooled day summaries (24 elements). The order-statistic ranks and every initialization constant are presented in **Supplementary Methods SM2.1, SM2.2**.

For the six threshold-enabled variables, the encoder additionally computes differentiable exposure counts, as defined in Equation 2. Each learned threshold *τ* is paired with a positive sharpness parameter *s*, so the count approaches a hard exceedance count as *s* decreases while remaining differentiable during training:

(2) $$c_{d,v,m}=\frac{1}{24}\sum_{r=1}^{24}\sigma \left(\frac{x_{d,r,v}-\tau_{v,m}}{s_{v,m}}\right),\;s_{v,m}\text{=softplus}\left(\varsigma_{v,m}\right)+10^{-3}$$

Thresholds are optimized on the standardized scale for numerical stability and can be transformed back to physical units. Section 3.2 evaluates their movement from initialization, cross-seed stability, and out-of-sample predictive relevance, with physical-unit values reported alongside these diagnostics in **Supplementary Table 17**.

The seven daily vectors are pooled by content-dependent attention, which avoids assigning meaning to an arbitrary weekday label and lets a short extreme episode outweigh uneventful days. The pooled vector is concatenated with the unweighted weekly mean.

The 39 variables are partitioned into the seven groups of **Supplementary Table 9**, each group is projected independently, and a second attention mechanism combines the group representations into the weekly station embedding. The group weights are an internal routing quantity, and Section 3.2 tests whether they track perturbation-based importance. Equation 3 defines the weekly station embedding and the group attention weights:

(3) $$E^{\text{week}}=\text{LayerNorm​}\left(\sum_{ɡ=1}^{G}\gamma_{ɡ}{\text{MLP}}_{ɡ}\left(f_{ɡ}^{\text{week}}\right)\right),\;\gamma_{ɡ}={\text{softmax}}_{ɡ}\left(a^{⊤}\tanh\left(We_{ɡ}\right)\right)$$

The observed weekly value for each station and lookback week enters the encoder path through a two-layer projection defined in Equation 3a. With ${\bar{p}}_{s,l}$, the observed weekly infestation value at station *s* and lookback week l on the [0, 1] scale of Section 2.1, clipped to [ϵ_ɡ_, 1 − ϵ_ɡ_],

(3a) $$h_{s,l}=W_{2}\text{GELU}\left(W_{1}{\left[{\bar{p}}_{s,l},\mathrm{logit}{\bar{p}}_{s,l}\right]}^{T}+b_{1}\right)+b_{2},\;{{\tilde{E}}_{s,l}}^{\text{week}}={E_{s,l}}^{\text{week}}+h_{s,l}$$

where W_1_ ∈ ℝ^{64×2}, W_2_ ∈ ℝ^{64×64}, the second term is the normalized output of Equation 3, and the sum is not renormalized. The projection is applied to all *L* = 8 lookback weeks, and the resulting sequence is the one passed to the thermal-time encoder. It contains 4,352 parameters (**Supplementary Table 1**). This projection makes the thermal-time encoder jointly history- and meteorology-informed. The gate described in Section 2.3.3 therefore compares the integrated encoder pathway with the convex history anchor.

#### 2.3.2 Thermal-time and cross-station encoding

Insect phenology is conventionally expressed in thermal time, the accumulation of degree-days above a base temperature, rather than in calendar time (Trudgill et al., 2005). TG-PestNet offers this scale to the temporal attention as an alternative to calendar distance between lookback weeks so that the effective memory span can depend on accumulated warmth; whether it improves on calendar distance is tested by retraining (Section 3.2). Hourly contributions are computed with a smooth rectifier of fixed sharpness *κ* = 4, a differentiable approximation to positive thermal accumulation (**Supplementary Methods SM2.4**).

The base temperature is constrained to a 5 °C–15 °C search interval that brackets published developmental thresholds for DBM and is initialized at 8 °C. It is treated as a predictive thermal-time parameter, with its fitted value and diagnostic evidence reported in Section 3.2.

Cumulative degree-days define the relative displacement between any two lookback weeks, as specified in Equation 4:

(4) $$\Delta_{l,m}=\frac{1}{{\text{dd}}_{\text{scale}}}\sum_{j\leq l}{\text{DD}}_{j}-\frac{1}{{\text{dd}}_{\text{scale}}}\sum_{j\leq m}{\text{DD}}_{j},\;b_{l,m}^{\left(n\right)}\text{=MLP}{\left(\left[\Delta_{l,m},\;e^{-\left|\Delta_{l,m}\right|}\right]\right)}_{n}$$

This displacement enters the temporal self-attention logits through a learned head-specific bias, as shown in Equation 5:

(5) $${\text{Attn}}^{\left(n\right)}=\mathrm{softmax}\left(\frac{Q^{\left(n\right)}K^{\left(n\right)⊤}}{\sqrt{d_{h}}}+b^{\left(n\right)}\right)V^{\left(n\right)}$$

The encoder uses two pre-normalized Transformer blocks, model width 64, four attention heads, GELU feed-forward layers, and dropout 0.10 (Vaswani et al., 2017).

All stations share the same hourly and thermal-time encoders. At each week, the resulting station representations interact through directed attention. For an edge from source station *i* to receiving station *j*, the model projects the weekly 100-m wind vector onto the geographic bearing from *i* to *j*, as defined in Equation 6:

(6) $$\rho_{i\to j,l}=u_{i,l}\cos\theta_{ij}+v_{i,l}\sin\theta_{ij},\;\rho_{i\to i,l}=0$$

The wind projection, normalized station distance, and an exponential distance term are passed to a head-specific multilayer perceptron to form an additive attention bias, as defined in Equation 7:

(7) $$\psi_{i\to j,l}^{\left(n\right)}\text{=MLP}{\left(\left[\rho_{i\to j,l}/w_{\text{scale}},\;D_{ij},\;e^{-D_{ij}}\right]\right)}_{n}$$

The receiving station supplies the query, so *i*→*j* and *j*→*i* can differ. The unconstrained multilayer perceptron accommodates the flexible, including nonmonotonic, effects of wind projection and distance. Section 3.2 separately evaluates the predictive value of wind inputs and the contribution and response shape of the edge-bias pathway.

#### 2.3.3 Decoder, history anchor, gate, and training objective

The decoder starts from the final encoded week. Rather than mapping the context directly to four unrelated levels, it predicts a bounded latent increment for each forecast step. The increment depends on the station context, target-week seasonal phase, and horizon embedding, as specified in Equation 8:

(8) $$r_{k}=r_{scale}\tanh\left(MLP([ctx,\psi (\omega_{k}),\zeta_{k}])\right)$$

Target week-of-year is represented by a Fourier basis with harmonic index *q*, as defined in Equation 9. This basis captures a recurrent annual structure that the meteorological tensor does not carry directly. The frozen preprocessing used the ISO week number with a denominator of 52; retraining with an exact day-of-year phase changed the macro RMSE by only +0.10% (**Supplementary Table 10**), indicating limited sensitivity to this encoding choice while supporting the continuous phase for calendar precision:

(9) $$\varphi \left(\omega \right)={\left[\sin\left(\frac{2\pi q\omega }{52}\right),\;\cos\left(\frac{2\pi q\omega }{52}\right)\right]}_{q=1}^{F}$$

Before conversion to logit space, the last observed DBM-infested plant percentage is clipped to the interval [ϵ_g, 1 − ϵ_g], where ϵ_g = 10^−4^. Equation 10 uses this endpoint-safe value and jointly defines the initialization, recursive latent-state update, and bounded state:

(10) $$z_{0}=\mathrm{logit}\left({\bar{p}}_{s,t}\right),\;z_{k}=z_{k-1}+r_{k}-\beta p_{k-1},\;p_{k}=\sigma \left(z_{k}\right)$$

A softplus reparameterization keeps the scalar *β* positive, and *β* concentrates a density-style adjustment in one explicit parameter. Because the state is expressed in logit space, the recursion remains compatible with a bounded rate after inverse transformation. The parameter functions as an explicit forecasting regularizer within the bounded latent-state recursion.

Infestation history is a strong short-range predictor, and several competitive baselines are smoothers of the 8-week history. Accordingly, TG-PestNet constructs a station- and horizon-specific convex history anchor, as defined in Equation 11:

(11) $$a_{s,k}=\sum_{l=1}^{L}w_{s,k,l}\;p_{s,\;t-L+l},\;w_{s,k,\cdot }=\mathrm{softmax}\left(\upsilon_{s,k,\cdot }\right)$$

This form contains LastValue and Mean8 as special cases and can learn intermediate lag profiles, so the learned lag weights summarize directly how each station and horizon use recent infestation history. The lag logits form a single 4 × 4 × 8 tensor of 128 parameters, initialized with logit 2 on the most recent lag so that the anchor starts from a LastValue-leaning profile (**Supplementary Methods SM2.6**).

Weekly DBM-infested plant percentages can equal zero, can occasionally equal one, and are otherwise continuous on (0, 1). A standard Gaussian head does not respect this support. The decoder therefore parameterizes an auxiliary zero-one-inflated Beta distribution for the encoder branch, drawing on beta regression for rates and proportions and its extension to endpoint-inflated data (Ferrari and Cribari-Neto, 2004; Ospina and Ferrari, 2012). The latent state supplies the interior Beta mean, while separate outputs determine precision and endpoint inflation. The resulting auxiliary encoder-branch distribution is given in Equation 12:

(12) $$f_{s,k}^{\left(w\right)}\left(y\right)=\pi_{s,k}^{0}\delta_{0}\left(y\right)+\pi_{s,k}^{1}\delta_{1}\left(y\right)+\pi_{s,k}^{\text{int}}\text{Beta}\left(y;\mu_{s,k}\nu_{s,k},\left(1-\mu_{s,k}\right)\nu_{s,k}\right)$$

The mean of this auxiliary encoder-branch mixture is:

(13) $$m_{s,k}^{\left(w\right)}=E_{f_{s,k}^{\left(w\right)}}\left[Y\right]=\pi_{s,k}^{1}+\pi_{s,k}^{\text{int}}\mu_{s,k}$$

Equation 13 gives the support-aware encoder-branch mixture mean used within the auxiliary training objective. Equation 14 subsequently combines this mean with the history anchor to produce the reported bounded point forecast.

The history anchor and the auxiliary encoder-branch mixture mean are combined by a station-horizon gate in logit space. Before applying the logit, both quantities are clipped to [ϵ_g, 1 − ϵ_g]; Equation 14 denotes the clipped quantities with overbars. Gate values near zero favor the history anchor, whereas values near one favor the encoder branch:

(14) $${\hat{y}}_{s,k}=\sigma \left(\left(1-{ɡ}_{s,k}\right)\mathrm{logit}\left({\bar{a}}_{s,k}\right)+{ɡ}_{s,k}\mathrm{logit}\left({\bar{m}}_{s,k}^{\left(w\right)}\right)\right),\;{ɡ}_{s,k}=\sigma \left(\xi_{s,k}\right)$$

The explicit gate provides a directly inspectable, station- and horizon-specific weight between the integrated encoder pathway and the convex history anchor, alongside the learned lag weights. The 16 gate logits form a 4 × 4 tensor initialized at zero, corresponding to an initial gate of 0.5. Because observed history also enters the encoder through the projection of Section 2.3.1, the fitted gate is interpreted as a pathway weight.

Equation 14 blends the two means in logit space to produce the final bounded point forecast; the endpoint probabilities of the auxiliary ZOIB head remain specific to the encoder-branch training objective.

In Equation 15, the first term is the masked negative log likelihood of the auxiliary encoder-branch ZOIB distribution. MAE and stabilized relative-error terms are applied to the final gated point forecast. These point losses optimize the history–encoder gate, whereas the auxiliary likelihood regularizes the encoder branch:

(15) $$L=L_{\text{ZOIB}}^{\left(w\right)}+\lambda_{1}L_{\text{MAE}}\left(\hat{y},y\right)+\lambda_{2}L_{\text{rel}}\left(\hat{y},y;\epsilon_{r}\right)$$

The auxiliary ZOIB term is the mean masked negative log likelihood of the encoder-branch distribution, the MAE term is the mean absolute error, and the relative-error term averages $\left|\hat{y}-\text{y}\right|/\max\left(\text{y},\epsilon \text{\_r}\right)$ over valid station-horizon targets, with λ_1_ = 2.0, λ_2_ = 1.0, ϵ_r = 0.05, and ϵ_g = 10^−4^.

#### 2.3.4 Audit of the fitted model and the frozen configuration

Six *post hoc* analyses were carried out on the 10 frozen final seeds over the held-out test split: group occlusion, which replaces every value in one physical-variable group with that group’s test mean; exact group-level Shapley values over all 2^7^ = 128 coalitions of the seven groups; thermal diagnostics that occlude the degree-day pathway and shift the whole temperature field from −4 °C to +4 °C; movement from initialization for every fitted physical and history parameter; Spearman rank agreement among attention, occlusion, and Shapley rankings with exact permutation *p*-values; and a counterfactual sweep of the downwind projection of Equation 6.

All interventions used a single inference harness that reproduced the stored frozen-checkpoint predictions exactly. Attention is analyzed as an internal routing quantity and triangulated with mean-imputation occlusion (Jain and Wallace, 2019; Ras et al., 2022) and exact Shapley attribution (Lundberg and Lee, 2017). Because mean-imputation occlusion is off-manifold, its magnitudes are interpreted comparatively, while retraining ablations determine which components materially support forecast performance.

The configuration those checkpoints came from was fixed as follows. Optimization was performed using AdamW with three parameter groups (Loshchilov and Hutter, 2019). The neural backbone, which includes the infestation-history projection, receives the base learning rate; the base temperature and β receive one-tenth of it and no weight decay, which limits abrupt movement away from their initial values; and the 128 lag logits and 16 gate logits receive 20 times it and no weight decay to promote the effective optimization of these compact parameter blocks. The learning rates for the three parameter groups are summarized in Equation 16:

(16) $$\eta_{\text{backbone}}=\eta ,\;\eta_{\text{physical}}=0.1\eta ,\;\eta_{\text{anchor}}=20\eta$$

Training used cosine annealing, gradient-norm clipping, early stopping, and deterministic seeding; after the configuration was fixed, the model was refitted on the combined training and validation data for 12 epochs under the minimum-of-mean-curve rule. Every frozen setting is shown in **Supplementary Table 1**, panel A, and the optimizer schedule in **Supplementary Methods SM2.7**. The frozen model has 246,757 trainable parameters in 101 tensors, reconciled by module in panel B and listed tensor by tensor in panel C; panel C also identifies one softmax-invariant final bias in the variable-group scorer. Independent re-evaluation of all 10 final checkpoints reproduces the stored test predictions to float32 round-off, and an automated test asserts the checkpoint hashes, the parameter total, the tensor count, and the module totals.

### 2.4 Evaluation design

#### 2.4.1 Chronological splits, baselines, metrics, and inference

Forecast origins were synchronized across stations. A sample was admissible only when all eight lookback weeks, all four target weeks, and all complete hourly weather blocks existed at every station. The 344-week common calendar permits 333 nominal origins. A 4-week boundary embargo, equal to the maximum forecast horizon, was imposed between training and validation and again between validation and test. This excludes the three intervening origin dates at each boundary, for six exclusions in total and 327 joint origins. Each origin contributes 16 targets (four stations × four horizons). **Supplementary Table 14** gives the resulting forward train–validation–test split.

The split is strictly forward in time, without shuffling or overlap, following the principle that forecast models must be assessed on observations later than those used for estimation and selection (Tashman, 2000). The test period is later than all observations used for fitting or architecture selection. Model selection was performed within the training-validation era using three expanding development folds (**Supplementary Table 14**, panel B). Each fold selects an epoch on an inner validation block, refits on the outer-fit block, and is scored on a held-out outer-evaluation block.

A hyperparameter search over these three folds compared 16 TG-PestNet candidates by mean inner-validation macro RMSE, averaged over the folds and three selection seeds (37–39), with the test split held out throughout. The reported model retained the hyperparameters recorded in the frozen configuration file dated July 25, 2026 for every searched dimension and subsequently incorporated the history-anchor block of Section 2.3.3. This architecture was frozen before final refitting on the combined training and validation data and evaluation on the test split (**Supplementary Table 1**, panel A).

The primary station-level comparison contains 12 benchmark families spanning classical machine learning and deep time-series forecasting (Hochreiter and Schmidhuber, 1997; Vaswani et al., 2017; Bai et al., 2018; Challu et al., 2023; Zeng et al., 2023): Ridge, random forest, Extra Trees, histogram gradient boosting, and support vector regression and TemporalConvNet, N-HiTS-small, NLinear, MLP, GRU, LSTM, and Transformer. All receive the same history-only input set—the eight most recent weekly values, 14 summary features of that window, sine–cosine encodings of the four target weeks, and station indicators—and no meteorological input. The column composition, the candidate grid of each family, and every selected configuration are shown in **Supplementary Methods SM3.1, SM3.2** and **Supplementary Table 19**. This comparison evaluates the complete weather-aware system against strong autoregressive alternatives, so its performance difference reflects the combined value of input information and architecture.

To examine whether conventional learners benefit from meteorological information, the same 12 families were also fitted locally, one estimator per station, under two paired feature conditions. The history-only condition is the input set above at local scope; the history-plus-weather condition adds 168 leakage-safe columns derived solely from meteorology observed before the forecast origin, built from the 14 weekly variables of **Supplementary Table 18** as an 8-week lagged sequence and four window statistics per variable. The two conditions share the same test keys, observations and seed sets, and hyperparameters were searched separately for each condition (**Supplementary Methods SM3.3**).

The paired experiment estimates the effect of adding this flat weather-summary block within each model family. TG-PestNet’s raw-hourly, shared-station and hierarchical representation is evaluated separately through the input-matched encoder controls of Section 2.4.2. A two-sided sign test summarizes the direction of the 48 model-by-station RMSE changes, and Diebold–Mariano tests compare origin-level squared-error losses within each pair.

Performance was evaluated on the original percentage scale using RMSE, MAE, and MAPE. For station *s* and forecast horizon *k*, RMSE is computed over the 66 test origins and then averaged equally across the four horizons. MAE and MAPE are pooled over the station’s 264 station–horizon test rows. MAPE excludes exact zeros and remains sensitive to small positive observations; RMSE and MAE are therefore reported in parallel as complementary absolute-error measures, with all three definitions given in **Supplementary Methods SM3.4**.

Each metric is computed independently for every seed and then averaged, and uncertainty is reported as the standard deviation of seed-level metrics. Per-cell best-baseline rankings are presented descriptively, while formal inference uses familywise-adjusted comparisons and paired bootstrap intervals.

Forecast-loss differences were assessed using the Diebold–Mariano test on origin-level squared-error differentials, TG-PestNet minus comparator, with a Newey–West heteroskedasticity- and autocorrelation-consistent variance estimator and the Harvey–Leybourne–Newbold small-sample correction to accommodate serial dependence from overlapping multi-step targets. Because no confirmatory reference was prespecified, nominal *p*-values are Holm-adjusted within each station. Every 95% interval on a relative difference comes from a hierarchical paired moving-block bootstrap that resamples forecast origins in blocks and draws the seed index jointly for both arms, preserving seed-level pairing; the primary analysis uses 8-week blocks and 2,000 replicates, with 4- and 12-week blocks reported as sensitivity checks in **Supplementary Table 7**. **Table 2**, panel B, reports complementary station-wise intervals against two post-selection references: a common history-only TemporalConvNet chosen after reviewing macro RMSE across comparators and the strongest tuned comparator at each station. These analyses together assess robustness to comparator choice (**Supplementary Methods SM3.4, SM3.5**).

**Table 2:** Forecasting performance and statistical separation.

Station	(A) Descriptive performance against the lowest-error comparator in each station–metric cell			
	RMSE	MAE	MAPE (%)	Lowest-error comparator, RMSE
Huiyang	10.749 ± 0.417	7.352 ± 0.375	53.10 ± 1.83	TemporalConvNet, 10.834
Shantou	3.032 ± 0.192	2.226 ± 0.123	14.24 ± 0.96	Extra Trees, 3.329
Shenzhen	6.407 ± 0.150	4.378 ± 0.209	108.35 ± 7.28	Raw-hourly TCN, weather off, 6.995
Dongguan	5.395 ± 0.284	4.052 ± 0.309	80.78 ± 7.92	Raw-hourly TCN, weather off, 5.714

Station	(B) Statistical separation			
	Reduction, RMSE/MAE/MAPE (%)	Holm-adjusted DM *p* < 0.05, *n*/12	Δ vs. history-only TemporalConvNet, 95% CI	Δ vs. strongest tuned comparator, 95% CI
Huiyang	0.8/4.4/12.7	2	−0.79% [−12.0, +4.7]	−0.79% [−12.0, +4.7]
Shantou	8.9/13.7/11.1	7	−19.31% [−36.0, −0.03]	−8.93% [−24.8, +1.9]
Shenzhen	8.4/12.1/27.4	12	−31.61% [−52.7, −9.7]	−8.40% [−19.9, +4.9]
Dongguan	5.6/5.0/0.7	11	−27.78% [−38.7, −7.5]	−5.59% [−11.0, +5.0]

RMSE and MAE are in percentage points; MAPE and reductions are percentages. TG-PestNet values are mean ± SD across seeds 40–49, with metrics computed per seed before averaging. In A, the comparator is selected separately within each station–metric cell, so the comparisons are descriptive; the MAE and MAPE comparators and the complete rankings of all 13 history-only families are presented in **Supplementary Table 2**. In B, the loss differential is TG-PestNet squared error minus comparator squared error, so negative values favor TG-PestNet, and the count is of Holm-adjusted Diebold–Mariano tests within that station across the 12 comparator families (**Supplementary Table 5**). Intervals come from the hierarchical paired moving-block bootstrap of Section 2.4.1 with 2,000 replicates and 8-week blocks, Holm-adjusted within each arm across the four stations. The history-only TemporalConvNet is the strongest tuned family at Huiyang but not elsewhere and was selected after the macro RMSE of every comparator was known; the second arm therefore reports the strongest tuned comparator at each station on the test period as a complementary, deliberately stringent comparison. Holm-adjusted bootstrap *p*-values are 0.478, 0.100, 0.006, and 0.004 in the first arm and 0.744, 0.404, 0.744, and 0.744 in the second. No interval changes side of zero at 4- or 12-week blocks except the Shantou first-arm interval, which includes zero at 12 weeks.

DM, Diebold–Mariano.

#### 2.4.2 Ablations, controls, and generalizability tests

Four sets of experiments evaluate the fitted system, all using the frozen configuration without re-selection. The first set concerns generalizability. In leave-one-station-out transfer, each station, in turn, has its labels excluded from the training loss and from epoch selection, while its weather and observed history remain inputs, so its gate and anchor logits stay at initialization; results are compared with the jointly supervised model from the same harness and with the LastValue and Mean8 anchors (**Supplementary Methods SM4.1**).

In year-ahead evaluation, each calendar year from 2018 to 2021 is held out in turn, the preceding complete year selects the refit epoch, all earlier origins form the training block, and the 4-week embargo separates them, giving 59, 111, 164, and 213 training origins. Nothing after the start of the test year enters the scaler, the epoch choice, or the fit. Comparators are the history-only TemporalConvNet re-tuned on pre-year data, LastValue, Mean8, and the no-cross-station variant (**Supplementary Methods SM4.2**). A third arm quantifies the forecasting value of extending the training history before 2015 by comparing the joint model, a capacity-matched self-edge variant and single-station models fitted on the common calendar and on the full archive (**Supplementary Methods SM4.3**).

The second set comprises 10 retrained structural variants and one optimization-sensitivity control. All use the frozen configuration, chronological split, same three tuning seeds, and same 10 final seeds without repeated hyperparameter search, providing controlled estimates of each named intervention under a common optimization protocol.

The variants are of two kinds. In the first, no module is bypassed and, at most, a few scalars are frozen: cross-station exchange is masked to the diagonal, the wind projection is zeroed while both distance terms are kept, the history anchor and the encoder branch are removed by freezing the 16 gate logits at *g* = 1 and *g* = 0, degree-day relative attention is replaced by calendar-week attention through the same bias network, the base temperature is frozen at its initialization, and the exposure-count channels are zeroed at the input. These seven hold the active parameter count at 246,757 and constitute the multiplicity family. Module-removal variants jointly alter structure and capacity and are therefore reported as a separate exploratory group outside the capacity-matched multiplicity family. The optimization-sensitivity control removes two near-zero-variance precipitation-group inputs to quantify sensitivity to rare training extremes while preserving validation- and test-time information. Every switch is specified in **Supplementary Methods SM3.6**.

Inference uses the bootstrap of Section 2.4.1 with 8-week blocks, Holm correction is applied within the family of seven, and the optimization-sensitivity control is reported in its sorted position in **Table 3** as a reference level rather than as a hypothesis.

**Table 3:** Retraining ablations with paired block-bootstrap inference.

Ablation	Macro RMSE	Δ (%)	Seeds with positive ΔRMSE (*n*/10)	95% CI (%)	Boot *p*	Holm *p*
Reference (un-ablated)	6.397	—	—	—	—	—
Remove history anchor (*g* = 1)	7.309	+14.26	10/10	[+6.3, +19.8]	<0.001	<0.001
Remove encoder branch (*g* = 0)	7.031	+9.91	10/10	[+2.3, +18.5]	0.011	0.066
No cross-station (self-edge)	6.659	+4.09	10/10	[−0.2, +6.9]	0.070	0.350
Mask threshold exposures	6.590	+3.03	8/10	[−1.5, +6.0]	0.203	0.812
Edge bias: distance only	6.585	+2.95	8/10	[−1.5, +5.9]	0.235	0.812
Optimization-sensitivity control	6.583	+2.91	8/10	[−1.8, +5.9]	0.272	—
Degree-day → calendar bias	6.581	+2.87	8/10	[−1.6, +5.9]	0.242	0.812
Fixed T_base at 8 °C	6.396	−0.01	5/10	[−0.03, +0.01]	0.980	0.980
Module-bypass variants with reduced active capacity (reported outside the correction family)						
Remove history projection	7.003	+9.48	10/10	[+3.0, +12.6]	0.004	—
Remove threshold-count features	6.624	+3.56	10/10	[+0.2, +6.0]	0.040	—
Remove cross-station layer	6.480	+1.30	7/10	[−3.1, +3.8]	0.797	—

Macro RMSE is the mean of the 16 station-by-horizon RMSE cells, averaged over the 10 final seeds; the across-seed SD of the reference is 0.168. Δ, its interval, and the bootstrap *p*-value come from the hierarchical paired moving-block bootstrap of Section 2.4.1 with 2,000 replicates and 8-week blocks. Holm correction is applied over the family of seven capacity-matched variants defined in Section 2.4.2, which excludes the optimization-sensitivity control; the optimization-sensitivity control is printed in its sorted position as a reference level. The three variants below the rule bypass a module, so capacity falls together with information. Active-parameter counts, per-variant contrasts between capacity-matched and bypassed implementations, and the 4- and 12-week block sensitivity checks are presented in **Supplementary Table 7**. In the *g* = 0 variant, the encoder branch still receives gradient from the auxiliary ZOIB likelihood but does not enter the point forecast; in the *g* = 1 variant, the 128 anchor lag logits remain trainable but receive no gradient and stay at their initialization.

The third set is representation controls. To test whether the flat weather block carries information at all, two conditions of identical width were built for three representative families at both feature scopes: a shuffled block, which preserves every marginal and the cross-variable structure while destroying only the correspondence between weather and target, and a noise block of independent standard normal draws. Each was drawn 50 times, and every draw re-selected hyperparameters under the same grid as the real block, so the control distribution carries selection variability as well as sampling variability; significance is a one-sided permutation *p*-value whose smallest attainable value is 0.02 at 50 draws (**Supplementary Methods SM3.7**).

The fourth set asks whether a conventional deep encoder can use the raw hourly tensor. Two generic architectures—dilated causal convolutions over the full 1,344-h window and self-attention over 56 daily tokens—receive the same hourly tensor as TG-PestNet while omitting its task-specific modules. Their readout heads additionally receive the same observed infestation history and target-week calendar values together with 14 engineered history summaries, providing a strong input-matched comparison. Capacity is held to within ±10% of TG-PestNet’s parameter count, and each architecture is repeated with the hourly tensor zeroed (**Supplementary Methods SM3.7**).

Two secondary analyses complete the design. The first descriptively re-aggregates test errors by the thermal conditions of the target week, which no model receives at forecast time, so the strata locate where errors occur without implying causality; the three stratifications partition the same 1,056 forecasts per seed by target-week mean temperature, maximum hourly temperature, and accumulated degree-days, with bin edges fixed before the errors were examined (**Supplementary Methods SM5.1**).

The second addresses the edge-bias network of Equation 7 directly, in two steps. An inference-time counterfactual sweep holds each station pair’s distance at its true value and replaces the downwind projection by 41 equally spaced values across the training-era range, recomputing the realized attention weight from the queries and keys captured from the frozen checkpoint; this gives one response curve per pair and seed, 120 curves in all, from which monotonicity, a violation fraction, and a downwind-minus-upwind difference are read (**Supplementary Methods SM5.2**).

A retrained variant then replaces the perceptron by a sign-constrained form that increases with downwind projection and decreases with distance by construction. The sign-constrained variant uses three active scalars per head instead of the 260-parameter perceptron, whose allocated weights receive no gradient; because active capacity differs, it is reported separately from the multiplicity family in **Supplementary Table 28** (**Supplementary Methods SM5.3**).

## 3 Results

Section 3.1 reports forecast accuracy against the history-only comparators and the statistical separation that supports it. Section 3.2 examines how meteorological representation affects forecast performance and audits every structured component of the model. Section 3.3 reports generalizability across stations and years together with the secondary robustness analyses.

### 3.1 Forecast accuracy and statistical separation

TG-PestNet produced the lowest observed RMSE, MAE, and MAPE across the four monitoring stations, giving 12 lowest-error cells out of 12 (**Table 2**, panel A). The mean reduction relative to the lowest-error comparator in each cell was 5.9% for RMSE, 8.8% for MAE, and 13.0% for MAPE; the RMSE reduction ranged from 0.8% at Huiyang to 8.9% at Shantou, MAE from 4.4% to 13.7%, and MAPE from 0.7% to 27.4%. Seven of the 12 cells are measured against the raw-hourly TemporalConvNet with its weather input switched off, which is the strongest history-only model in this study; against the 12 tuned baseline families alone the reductions would be larger. The largest reduction is Shenzhen MAPE, and the smallest is Dongguan MAPE, where the two models are within 0.7% of each other and should be read as tied. The complete rankings for every family and metric are presented in **Supplementary Table 2**.

The cell-wise comparisons of panel A are descriptive because the reference model is selected separately after observing each test cell, and MAPE excludes exact zeros and is sensitive to small positive observations, which is why its absolute values remain high at Shenzhen and Dongguan. Seed-to-seed variability was modest: across seeds 40–49, the RMSE standard deviation reached at most 0.417 percentage points and the MAE standard deviation at most 0.375.

Across all 12 baseline families, the mean squared-error loss differential favored TG-PestNet at every station. After Holm adjustment within station, TG-PestNet achieved significantly lower loss than two of 12 comparators at Huiyang, seven at Shantou, 12 at Shenzhen, and 11 at Dongguan (**Table 2**, panel B), with the clearest separation at Shenzhen and Dongguan; comparator-specific estimates are provided in **Supplementary Table 5**. These analyses together provide comparator-wide evidence, with no single baseline specified as a confirmatory reference.

At Huiyang, TG-PestNet’s mean RMSE was 0.085 percentage points lower than that of the history-only TemporalConvNet, and it achieved lower RMSE in six of the 10 paired seeds (**Supplementary Table 21**). Panel B of **Table 2** provides the paired block-bootstrap intervals. Against the history-only TemporalConvNet used at all four stations, the advantage was separable from zero at Shenzhen and Dongguan after Holm adjustment, but not at Shantou or Huiyang; against the strongest tuned comparator at each station, no interval excluded zero at any of the three block lengths. Panel B therefore provides the inferential context for the descriptive ordering in panel A.

### 3.2 Weather representation and structured-component audit

Forecast performance depended strongly on how meteorological information was represented. When the 12 baseline families were rerun locally under paired history-only and history-plus-weather conditions, adding the 168-column flat weather block increased the RMSE in 41 of the 48 model–station pairs, with a median station-level change of +21.6% and a two-sided sign test on direction giving *p* = 6.2 × 10^−7^ (**Table 4**, panel A). The direction was consistent at every station, and the increase was organized more strongly by model family than by station: families with shrinkage or built-in feature selection showed smaller median increases than gradient-trained networks receiving the raw block (**Supplementary Figure 3**). Full local values and paired tests are provided in **Supplementary Table 6**.

**Table 4:** Effects of meteorological representation and structured inputs.

A. Adding the 168-column flat weather block to 12 local baseline families				
Model–station pairs with higher RMSE after weather is added			41 of 48	
Median station-level change in RMSE after adding weather			+21.6%	
Two-sided sign test on the direction of the 48 changes			*p* = 6.2 × 10^−7^	
Model-by-scope cells in which a matched-width information-free block was at least as good			10 of 12	

B. Input-matched raw-hourly encoders (macro RMSE, percentage points)				
Model	Macro RMSE	Δ vs. TG-PestNet	95% CI	*p*
TG-PestNet	6.397 ± 0.168	—	—	—
Raw-hourly TCN	8.085 ± 0.140	+26.4%	[+15.8, +35.4]%	<0.001
Raw-hourly TCN, hourly tensor zeroed	7.336 ± 0.091	+14.7%	[+3.9, +21.8]%	0.006
Raw-hourly transformer	7.986 ± 0.189	+24.8%	[+14.3, +33.9]%	<0.001
Raw-hourly transformer, hourly tensor zeroed	7.646 ± 0.236	+19.5%	[+6.9, +28.1]%	<0.001
Within architecture: TCN, with minus without	—	+10.2%	[+3.7, +19.0]%	0.006
Within architecture: Transformer, with minus without	—	+4.5%	[−2.4, +14.7]%	0.169

C. Three estimates of what meteorology contributes to TG-PestNet				
Intervention	Macro RMSE	Δ	95% CI	*p*
Occlusion of the frozen model: how strongly the fitted model relies on meteorology	—	+9.28%	—	0.001
Encoder branch bypassed: removes the branch and the history projection within it	7.031	+9.91%	[+2.3, +18.5]%	0.077
Retrained with meteorology zeroed: retraining estimate of meteorological contribution	6.625 ± 0.112	+3.57%	[−0.45, +8.98]%	0.064
Retrained with meteorology zeroed, Huiyang alone	—	+9.17%	[+1.7, +19.7]%	0.018

Panel A summarizes the paired history-only versus history-plus-weather experiment on 12 local baseline families together with the matched-width shuffled and noise controls; per-family values are presented in **Supplementary Tables S6** and **S24** and **Supplementary Figure 3**. Panel B is given in full in **Supplementary Table 25**. Δ, its interval, and *p* come from the paired block bootstrap of Section 2.4.1 with 2,000 replicates and 8-week blocks, and Holm correction is applied across the six comparisons of panel B; no interval there changes side of zero at 4 or 12 weeks. Panel C is descriptive, and its *p*-values are uncorrected because the three interventions act on different objects and are not competing tests of one hypothesis; the Huiyang row is Holm-adjusted to 0.072 across the four stations, and the other three stations are Shenzhen +2.76%, Dongguan −2.35%, and Shantou −3.88%, all with intervals containing zero. For context, TG-PestNet retained its default configuration, whereas each generic encoder’s reported configuration was selected from a 144-fit search (Section 2.4.1).

Matched-width controls indicated that dimensionality alone did not explain the pattern. Against shuffled and noise blocks of identical width, drawn 50 times with hyperparameters re-selected on every draw, an information-free block matched or outperformed the real block in 10 of the 12 model-by-scope cells (permutation *p* between 0.63 and 1.00). In the two remaining cells—global Ridge, where no control draw of 50 matched the real block (*p* = 0.020), and the local TemporalConvNet, where three did (*p* = 0.078)—the real block produced smaller RMSE increases than noise, and neither comparison remained significant after Holm correction across the 12 cells. The real block still increased the RMSE relative to history alone; for the two regularized local families, shuffled blocks produced lower RMSE than the real block in 48 and 50 of 50 draws, consistent with training-period associations that did not persist into the test period. Thus, the flat representation did not provide statistically resolved incremental information in this sample (**Supplementary Table 24**).

The same pattern was observed in the input-matched deep encoders. Two generic architectures received the identical hourly tensor, the same observed infestation history and target-week calendar values, and a matched parameter and search budget; their readout heads also received 14 engineered history summaries that TG-PestNet does not use. The raw-hourly TCN and Transformer had macro RMSE values 26.4% and 24.8% above TG-PestNet, respectively. Within architectures, zeroing the hourly tensor reduced TCN RMSE by 10.2% (95% CI [+3.7%, +19.0%]) and Transformer RMSE by 4.5% ([−2.4%, +14.7%]), with the former statistically resolved and the latter not (**Table 4**, panel B; **Supplementary Table 25**). Against every local baseline under both feature conditions, all 96 loss differentials favored TG-PestNet, and 74 were nominally significant. These comparisons indicate that input dimensionality alone does not explain TG-PestNet’s advantage and support the tested structured system as a whole. TG-PestNet additionally combines hierarchical compression, joint four-station fitting, and a protected infestation-history pathway; the ablations below assess their contributions.

Three interventions estimate meteorological contributions to TG-PestNet, and panel C of **Table 4** presents them together because they answer distinct questions. Occluding weather in the frozen model increased the macro RMSE by 9.28%, quantifying fitted-model reliance on meteorology. Bypassing the encoder branch increased the macro RMSE by 9.91% while also removing the infestation-history projection within that branch. Retraining with every meteorological input held at its training mean increased the macro RMSE by 3.57% (95% CI [−0.45%, +8.98]; *p* = 0.064), directly evaluating whether meteorology was needed to reach the observed accuracy. Although the four-station macro effect was not statistically resolved, the retrained model reached 6.625 and remained ahead of the strongest history-only comparator at 7.336. Station-level effects varied: retraining without meteorology increased the RMSE by 9.17% at Huiyang (95% CI [+1.7%, +19.7%]; all 10 paired seed-level differences were positive; uncorrected *p* = 0.018; Holm-adjusted *p* = 0.072) and by 2.76% at Shenzhen, whereas point estimates were −2.35% at Dongguan and −3.88% at Shantou, with both intervals containing zero. The clearest station-specific meteorological contribution occurred at Huiyang, although it did not remain significant after Holm adjustment across the four stations; contributions at the other three stations were not statistically resolved.

The fitted-model audit clarifies how its named components should be interpreted. Across the 10 frozen seeds, T_base moved by at most 0.006 °C from its 8.00 °C initialization, below 0.1% of its value and negligible relative to the 5 °C–15 °C constraint interval; it is, therefore, treated as an operational model parameter rather than a data-estimated biological threshold. Occluding the degree-day pathway changed the macro RMSE by −0.0065%, and the uniform −4 °C to +4 °C perturbation was monotonic rather than minimized at zero. The thermal variable group showed modest, method-dependent effects: occlusion increased the macro RMSE by 0.60% (*p* = 0.100), while its exact group Shapley value was +0.0988 RMSE units (*p* = 0.014).

Learning-rate sweeps assessed whether the limited movement of the physical parameters reflected optimization or weak identification. At the backbone rate, the base temperature moved by at most 0.05 °C with unchanged accuracy; at 10 times that rate, it moved by at most 0.23 °C, and the macro RMSE increased by 3.10% (95% CI [−1.44%, +5.98%]); at 20 times the rate with a 60-epoch refit, the fitted values ranged from 6.39 °C to 10.18 °C across seeds, while macro RMSE increased by 7.15% (**Supplementary Table 22**). This cross-seed dispersion without improved forecast accuracy indicates weak identification. Accordingly, the reported 8 °C value denotes initialization rather than an empirically estimated developmental threshold.

The learned exposure thresholds likewise remained close to initialization, moving by at most 0.005 standardized units across the 10 seeds. Four of the 24 thresholds lie below the physical minimum of their non-negative variables, so every station-hour in the window exceeds them; under the fitted sharpness, these channels therefore operate as compressed monotone transforms rather than exposure cut-points. All 24 physical-unit values are provided in **Supplementary Table 17**.

**Figure 2** summarizes the audit. The all-weather occlusion of **Table 4** (panel C) raised error in all 10 seeds (*t* = 4.79, *p* = 0.001), so the fitted model relies on meteorology; the infestation-history projection remains active under this intervention. Among individual groups, significant occlusion effects were found for radiation (+1.18%, *p* = 0.033), wind (+0.87%, *p* = 0.007), and pressure/cloud (+0.99%, *p* < 0.001). By contrast, the base temperature, density-style term, and hour-of-day weights remained near their initial states, while the history-anchor lag weights moved by 37% and the gate by 17% on the convex-weight and gate scales (**Figure 2E**). This movement confirms that optimization updated the history parameters, and the retraining ablations below evaluate whether those updates translate into forecasting contributions.

**Figure 2:**
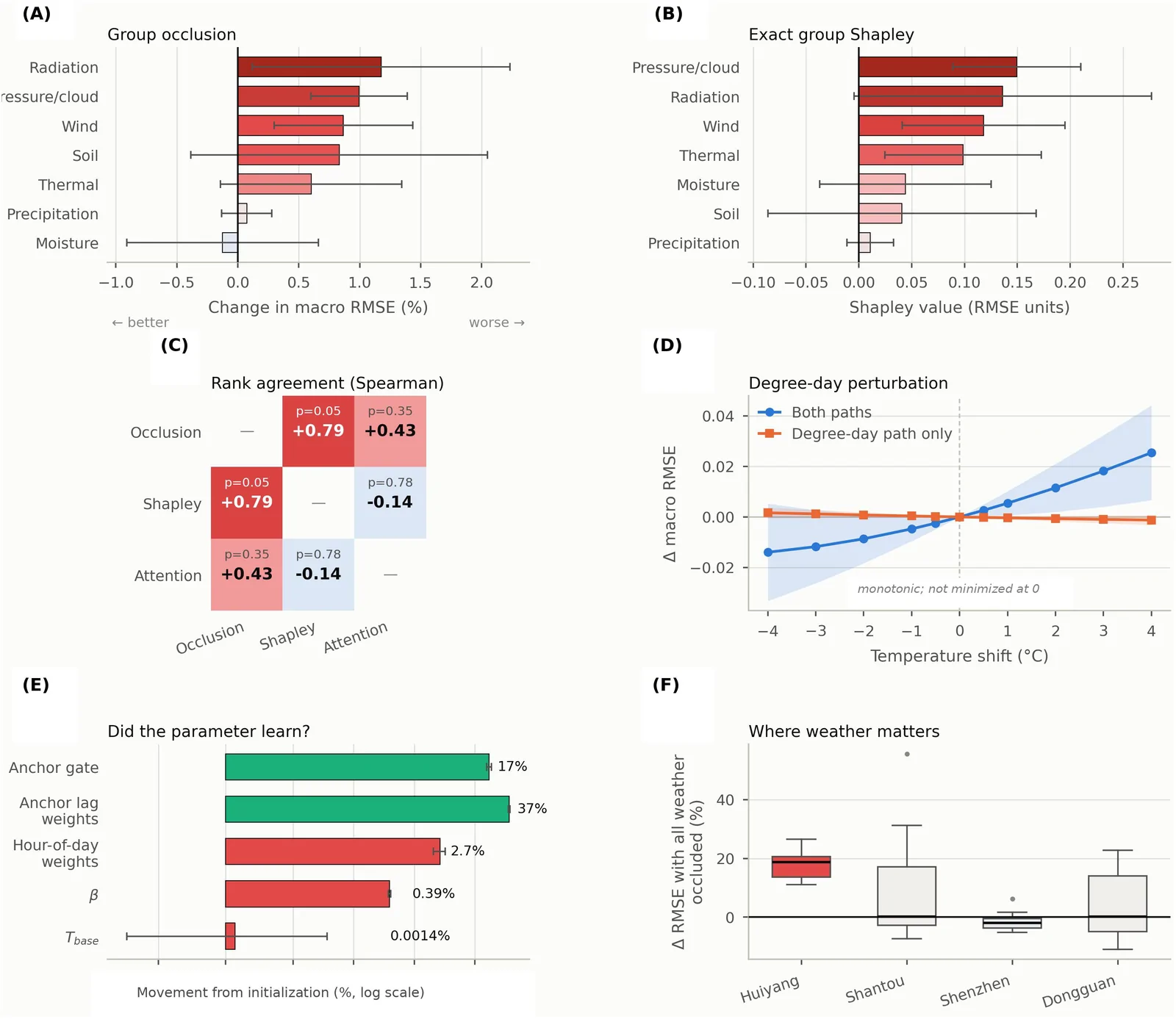
Empirical audit of the structured components. **(A)** Change in macro RMSE after physical-group occlusion. **(B)** Exact group Shapley values over all 128 coalitions. **(C)** Rank agreement among occlusion, Shapley and group attention. **(D)** Temperature perturbation with both thermal paths or the degree-day path alone. **(E)** Parameter movement from initialization. **(F)** Effect of occluding all weather by station. Error bars, bands and boxplots summarize variation across the ten frozen seeds.

Occlusion and exact Shapley values produced broadly concordant seed-averaged rankings (Spearman *ρ* = 0.79, exact permutation *p* = 0.05), whereas attention–Shapley rank agreement was low (*ρ* = −0.14, *p* = 0.78; **Supplementary Table 4**); the attention weights themselves spanned 0.1334–0.1549 around the uniform value of 0.1429. Across seeds, the positive-versus-negligible grouping was more stable than the precise ordering within either group, with within-method rank reliabilities below 0.40. Accordingly, attention weights are interpreted as routing coefficients rather than variable-importance estimates.

The all-weather intervention was strongly station dependent: RMSE increased by 18.3% at Huiyang, 9.8% at Shantou, and 3.6% at Dongguan and decreased by 1.4% at Shenzhen. The macro percentage is computed from aggregated RMSE and is not the arithmetic mean of the four station values. The fitted model therefore uses meteorology unevenly across stations; whether meteorology is necessary is a different question, which is answered by the retraining reported above.

To complement the frozen-checkpoint audit with training-level evidence, 10 structural variants were retrained under the protocol of Section 2.4.2 and are reported in **Table 3** alongside an optimization-sensitivity control that removes two sparse precipitation-group inputs. Removing them changes no validation- or test-time information yet shifted the macro RMSE by +2.91% (95% CI [−1.77%, +5.95%]). Snow depth was identically zero, while 11 snowfall hours standardized to approximately +165 σ and affected eight training origins; removing the pair, therefore, changed the optimization trajectory. The +2.91% point estimate provides a contextual benchmark for optimization sensitivity in this training design rather than a universal resolution threshold. Its interval spans more than seven percentage points, indicating that effects of this order remain statistically unresolved.

Two variants freeze the gate of Equation 14. Because the equation blends in logit space, freezing at *g* = 1 leaves the encoder branch as the sole contributor to the point forecast, whereas freezing at *g* = 0 leaves the convex 8-week anchor as the sole contributor. Because the encoder branch also receives the 8-week infestation history through the projection of Section 2.3.1, the *g* = 1 variant remains jointly history- and meteorology-informed. **Table 3**, therefore, names each gate row by the pathway removed. Removing the history anchor increased the macro RMSE by 14.26% (95% CI [+6.3%, +19.8%]; Holm *p* < 0.001) and remained significant after multiplicity correction. Removing the encoder branch increased the macro RMSE by 9.91% (95% CI [+2.3%, +18.5%]; Holm *p* = 0.066), with the interval excluding zero before multiplicity adjustment. Bypassing the 4,352-parameter infestation-history projection of Section 2.3.1 increased the macro RMSE by 9.48% (95% CI [+3.0%, +12.6%]; uncorrected *p* = 0.004). These interventions together quantify how the encoder branch and recent infestation history are combined.

The gated combination improved forecast accuracy beyond the protected history path alone. With *g* = 0, only the protected history path contributes to the point forecast, while the active parameter budget is unchanged and the anchor’s lag weights remain trained through the point-forecast terms; the macro RMSE increased by 9.9% relative to the gated model. This result shows that the encoder branch contributes information beyond the convex 8-week anchor. Because the encoder branch also contains an infestation-history projection, the all-weather occlusion reported above, which deliberately leaves that projection active, separately evaluates the fitted model’s reliance on meteorology.

Pathway contributions varied among stations. Removing the history anchor increased the RMSE by 38.5% at Shantou, 23.8% at Dongguan, 13.6% at Shenzhen, and 2.9% at Huiyang, whereas removing the encoder branch changed the RMSE by +17.8% at Huiyang, +9.9% at Dongguan, +2.5% at Shenzhen, and −2.0% at Shantou. The fitted gate followed the encoder-branch ordering—0.70 at Huiyang, 0.54 at Dongguan, 0.46 at Shenzhen, and 0.43 at Shantou (**Supplementary Table 16**)—a descriptive correspondence given the four-station sample. All-weather occlusion produced a different station ordering (*ρ* = 0.40 across four points), while Huiyang showed the clearest agreement across the gate, occlusion, and retraining analyses.

Cross-station exchange produced positive but statistically unresolved point estimates. Restricting the layer to self-edges retained all modules and increased the macro RMSE by 4.09%, whereas bypassing the layer removed the same cross-station information together with 17,284 active parameters and increased the macro RMSE by 1.30%; both intervals included zero. Because layer bypass also changes capacity and regularization, the difference between these estimates does not isolate the pure information-flow effect. The corresponding mask-versus-bypass estimates for the exposure-threshold channels were +3.03% and +3.56%.

Three capacity-matched thermal and dispersal interventions yielded point estimates close to the optimization-sensitivity control’s +2.91%: dropping the wind term from the cross-station edge bias while retaining both distance terms (+2.95%), replacing degree-day relative attention with calendar-week relative attention (+2.87%), and masking the learned exposure-threshold channels (+3.03%). All intervals included zero and all adjusted *p*-values were 0.812, so their independent forecasting contributions were not statistically resolved in the present experiment.

Wind contributed differently at the input-group and edge-bias levels. Occluding the wind input group increased the macro RMSE by 0.87% (*p* = 0.007), and these variables remain in the 39-variable input set in every reported variant. Removing the wind projection from the cross-station edge bias increased the macro RMSE by 2.95%, with an interval containing zero. Section 3.3 further evaluates the monotonicity and seed stability of the edge-bias response.

Masking and bypassing the threshold-exposure channels increased the macro RMSE by 3.0% and 3.6%, respectively; all 10 paired seed-level differences for the bypass were positive, and its unadjusted interval excluded zero. Because these estimates overlap the optimization-sensitivity range, the channels’ independent forecasting contribution remains statistically unresolved.

The degree-day comparison is like-for-like: the degree-day and calendar displacements enter the same bias network with the same parameter count, and because the accumulation scale is 100 °C·day while a week accumulates about 98 °C·day at these sites, the two inputs are also on the same numerical scale. Under this matched parameterization and similar input scale, the accuracy difference between thermal-time and calendar-time relative attention was not statistically resolved in this dataset.

Freezing the base temperature at 8 °C changed the macro RMSE by −0.01% (95% CI [−0.03%, +0.01%]). Because the parameter remained within 0.006 °C of its initialization, the fixed and learned variants were numerically similar. The narrow interval therefore reflects similarity between the fitted models rather than precision in estimating a biological quantity; the base temperature is treated as an initialization value rather than a data-identified thermal threshold.

The relation between removed parameter count and RMSE change was non-monotone: the three bypass variants removed 1.8%, 7.0%, and 8.1% of parameters while changing the macro RMSE by +9.48%, +1.30%, and +3.56%, respectively. This pattern indicates that computation-specific changes, rather than parameter count alone, shaped the outcomes; the present bypass comparisons do not isolate pure capacity effects because each removes a different computation together with its parameters. Per-variant contrasts and 4- and 12-week block sensitivity checks are reported in **Supplementary Table 7**. Two preprocessing corrections retrained under the same protocol are reported in **Supplementary Table 10** and yielded the same section-level interpretation.

### 3.3 Generalizability and secondary robustness analyses

Transfer performance varied among stations. Withholding station labels increased the RMSE by 6.8% at Shenzhen, 17.3% at Dongguan, 23.9% at Huiyang, and 61.8% at Shantou relative to joint supervision (**Table 5**). Relative to LastValue at the same station, the transferred model achieved lower RMSE in nine of 10 seeds at Dongguan and seven of 10 at Shenzhen; at Huiyang, its mean RMSE was higher, although six of 10 paired seeds were lower, and at Shantou it was lower in one of 10 seeds. The largest increase and seed-to-seed spread occurred at Shantou, where the jointly supervised forecast relied strongly on the history anchor, while the withheld-station protocol used a default gate of 0.5 (Section 2.4.2); with four stations, this correspondence is descriptive rather than causal. These results motivate station-specific local calibration, particularly at Shantou, before deployment at a station whose labels were not used for training.

**Table 5:** Leave-one-station-out transfer (RMSE in percentage points, mean ± SD over seeds 40–49).

Withheld station	Transfer, labels withheld	Jointly supervised	Δ vs. joint supervision (%)	LastValue	Δ vs. LastValue	Seeds better than LastValue
Shenzhen	6.857 ± 0.688	6.419 ± 0.127	+6.8%	6.962	−1.5%	7/10
Dongguan	6.359 ± 0.584	5.421 ± 0.235	+17.3%	7.356	−13.6%	9/10
Huiyang	13.252 ± 1.398	10.699 ± 0.262	+23.9%	12.976	+2.1%	6/10
Shantou	4.931 ± 1.762	3.048 ± 0.223	+61.8%	3.139	+57.1%	1/10

The jointly supervised column is the identity control of the same harness, which retrains from the frozen configuration rather than reloading the frozen checkpoints and reproduces the frozen macro RMSE to 0.001. LastValue is computed on the same 66 test origins with the same equal-weight macro over horizons.

In year-ahead evaluation (**Table 6**), TG-PestNet had lower macro RMSE than LastValue in every test year, by 8.3%–17.8%, and each difference remained significant after Holm correction. This consistency across years supports temporal robustness beyond the terminal test block of Section 3.1. Against a history-only TemporalConvNet re-tuned each year, the smaller network had the lower point estimate with 59 and 111 training origins, whereas TG-PestNet had the lower point estimate with 164 and 213; only the 2021 difference (15.7%, 95% CI 7.6%–22.2%) was separable from zero. Because training-set size and test year changed together, these comparisons characterize year-specific performance rather than identifying a sample-size threshold; removing cross-station exchange changed the year-ahead accuracy by less than the seed spread in every year. Re-aggregated by target-week meteorological season (**Supplementary Table 12**), TG-PestNet had lower macro RMSE than LastValue in all four seasons, by 6.2% in December–February, 16.4% in March–May, 11.0% in June–August, and 11.6% in September–November; error was highest in the cool season, when infestation peaks in Guangdong (Li et al., 2012, 2016b).

**Table 6:** Year-ahead evaluation by test year (macro RMSE in percentage points).

Test year	Training origins	TG-PestNet	No cross-station	TemporalConvNet	LastValue	Mean8	Δ vs. LastValue
2018	59	10.255 ± 0.346	10.102 ± 0.244	9.921 ± 0.115	11.182	13.284	−8.3%
2019	111	11.784 ± 0.348	11.470 ± 0.209	11.467 ± 0.120	13.577	13.212	−13.2%
2020	164	7.790 ± 0.230	7.921 ± 0.190	8.561 ± 0.137	8.909	8.627	−12.6%
2021	213	6.152 ± 0.247	6.360 ± 0.106	7.115 ± 0.109	7.481	8.255	−17.8%
4-year macro		8.995	8.963	9.266	10.287	10.844	−13.0%

The final column of the macro row is the mean of the four yearly reductions; computed from the macro RMSE values, it is −12.6%.

Restoring the pre-2015 history changed the single-station test RMSE by −8.95% at Shantou (95% CI [−19.8%, +2.4]) and +5.63% at Huiyang ([−1.5%, +16.9]%), with Holm *p* = 0.71 for both (**Supplementary Table 26**). Both intervals included zero and were sufficiently broad that this experiment could resolve only effects on the order of ±10 percentage points. Against single-station models fitted to the full archive, the joint model changed the RMSE by −5.84% at Huiyang ([−12.5%, −1.3%]; uncorrected *p* = 0.018; Holm *p* = 0.14) and +9.39% at Shantou. The Huiyang interval was the only one of the eight to exclude zero, doing so with 8- and 12-week blocks, but not with 4-week blocks.

The Huiyang estimate may reflect differences between the pre-2015 population and management regime and the 2020–2022 test period, incomplete protocol metadata for the earlier records, or a configuration selected with 195 origins and then applied to 797. Because these factors vary together, the comparison does not isolate the accuracy effect of common-calendar synchronization. The synchronization requirement enabled cross-station exchange while reducing the available station-specific history, quantifying a data-retention trade-off, and motivating asynchronous-history designs that retain longer records without requiring complete calendar overlap.

When the same test forecasts are re-aggregated by the thermal regime of the target week, error was largest in the cool, low-degree-day strata and smallest in the warm, high-degree-day ones: macro RMSE was 7.06 below 20 °C against 4.95 at 25 °C –30 °C and 7.13 in the lowest degree-day tercile against 5.02 in the highest (**Supplementary Table 27**). This follows the seasonal course of infestation, which peaks in the cool season in Guangdong (Li et al., 2012, 2016b): absolute error is largest where the observed level is largest. The ordering is not uniform across stations—it holds at Huiyang, is reversed at Shantou, and is not monotone at Shenzhen or Dongguan (**Supplementary Table 20**)—so the pooled ordering is carried by Huiyang, the station with the highest and most variable test-period infestation.

Across the eight occupied thermal strata, replacing degree-day relative attention with calendar-week attention changed the macro RMSE by −2.24% to +6.14%, and masking the exposure-count channels changed it by −1.77% to +6.01%; every 95% interval included zero. Thus, the within-stratum contributions of the two thermal components were not statistically resolved under the evaluated conditions.

The reported model had lower macro RMSE than both the LastValue anchor and the raw-hourly encoder in every occupied thermal stratum. The test set contained no forecasts in two extreme-temperature strata: the warmest target-week hour was 37.5 °C, and the warmest weekly mean was 29.8 °C, leaving the ≥38 °C maximum-temperature and >30 °C mean-temperature strata empty. Behavior near the reported larval-survival boundary therefore remains outside the support of the current evaluation data.

The wind edge bias was audited directly by counterfactual sweep. Across the 120 pair-by-seed response curves, realized cross-station attention increased monotonically with the downwind projection in 78, was non-increasing in 32, and was non-monotone in 10. The mean violation fraction was 0.293, and the 42 curves that were not monotone increasing moved against the intended direction on 84% of their steps. The mean downwind-minus-upwind difference was +0.0010 against a mean realized weight of 0.246 or 0.4% of the weight itself; for every station pair, its standard deviation across seeds exceeded its absolute mean, indicating a small and seed-variable response. The fitted bias alone showed a nearly identical pattern, locating the non-monotonicity in the fitted perceptron (**Supplementary Table 28** and **Supplementary Figure 2**).

Two retraining results complete the analysis. Removing the wind term from the edge bias while retaining both distance terms changed the macro RMSE by +2.95% (95% CI [−1.5%, +5.9%]), and constraining the bias to the intended signs changed it by +2.55% ([−1.8%, +5.4%]). Both point estimates were close to the optimization-sensitivity control’s +2.91%, and every interval included zero at all three block lengths. These accuracy differences were not statistically resolved, and the intervals do not establish equivalence among variants. The analyses together show fitted-model sensitivity to the wind input group and a small, seed-variable edge-bias response. Equation 7 is therefore interpreted as a forecasting bias rather than an identified dispersal mechanism.

## 4 Discussion

### 4.1 What the study establishes

This study evaluated whether a high-frequency, wind-informed multi-station network could improve direct 1- to 4-week forecasts of the DBM-infested plant percentage. TG-PestNet achieved the lowest descriptive RMSE, MAE, and MAPE in all 12 station–metric cells, and all 96 comparisons with local history-only and history-plus-weather baselines favored it. Performance gains were directionally consistent across stations, although uncertainty varied spatially: separation was strongest at Shenzhen and Dongguan, moderate at Shantou, and smaller at Huiyang; station-specific intervals against the strongest tuned comparator included zero.

Another important finding concerns how meteorological information enters the tested systems. Adding a flat 168-column weather block to generic local learners increased the error in 41 of 48 model–station pairs, with a median change of 21.6%, and matched-width controls detected no stable incremental test-period benefit from that representation in 10 of 12 model-by-scope cells. Two generic deep encoders given the same raw hourly tensor, observed infestation history, and a matched budget were less accurate than TG-PestNet, and zeroing the tensor improved one encoder while leaving the other statistically unresolved. These controls together indicate that input dimensionality alone does not explain the cross-architecture gap. The capacity-matched ablation identifies the protected history anchor as a major contributor, while targeted factorial ablations can further partition the roles of hierarchical compression and joint four-station fitting.

The audit supports a focused interpretation of the fitted components. Retraining without meteorology increased the four-station macro RMSE by 3.6%, while the model still outperformed every tested history-only comparator. Effects varied by station: Huiyang showed a 9.2% increase with an unadjusted interval excluding zero, although this result did not survive Holm adjustment across four stations (*p* = 0.072), while Dongguan and Shantou showed small negative point estimates. The protected history anchor was the clearest isolated contributor, increasing the error by 14.3% when removed and remaining significant after multiplicity correction. The remaining audits position the thermal, attention, and spatial components as testable representational hypotheses and define concrete targets for future validation.

### 4.2 Biological, agronomic, and surveillance implications

ALIC combines feature attention, recurrent encoding, and interaction convolution over compact monthly weather and pest history (Wang and Zhang, 2024), while MeteoSCOPE emphasizes structured multidimensional meteorological memory (Zhang and Wang, 2026). The present findings extend that line of work by moving to a weekly, multi-station scale that begins with hourly reanalysis and by auditing the resulting components rather than reading their attention weights.

Relative to generic sequence models, the main contribution is not depth alone but the way the hypothesis space is constrained. LSTM, temporal convolution, N-HiTS, NLinear, and Transformer families provide flexible sequence representations (Hochreiter and Schmidhuber, 1997; Vaswani et al., 2017; Bai et al., 2018; Challu et al., 2023; Zeng et al., 2023), yet the paired history-plus-weather experiment shows that simply adding a large weather block often reduces accuracy. One plausible explanation for TG-PestNet’s robustness is the protected history path, which allows recent infestation history to remain available while meteorological and spatial information modify the forecast.

Temperature remains biologically central to DBM risk: development and reproduction accelerate within favorable ranges, accumulated warmth shifts phenology, oviposition declines above approximately 30 °C, and larval survival is constrained near more extreme temperatures (Trudgill et al., 2005; Marchioro and Foerster, 2012; Bahar et al., 2014; Ngowi et al., 2017; Wang and Zhang, 2024). These findings motivated the use of hourly exposure and thermal accumulation. In this dataset, forecast errors concentrated in cooler, low-degree-day weeks, where infestation peaks in Guangdong (Li et al., 2012, 2016b), and the thermal components added little measurable accuracy. Because the warmest observed hour was 37.5 °C, evaluation in warmer regimes is needed to test behavior near the approximately 38 °C larval-survival boundary.

Weather dependence varied across sites. All-weather occlusion increased the error by 18.3% at Huiyang, 9.8% at Shantou, and 3.6% at Dongguan while reducing it by 1.4% at Shenzhen; this pattern differed from the raw forecasting gains, which were largest at Shenzhen and Dongguan. Ablations suggest complementary station-specific roles: history-anchor effects were largest at Shantou, Dongguan, and Shenzhen, encoder-branch effects were largest at Huiyang, and Dongguan benefited from both pathways. Retraining without meteorology produced the same qualitative pattern: Huiyang was the only station whose unadjusted interval excluded zero, although no station-specific result survived Holm correction. Future station-level analyses incorporating crop stage, host continuity, and management can test these alternatives.

The response variable is the percentage of inspected plants classified as DBM-infested rather than a direct demographic measure, and recorded infestation is jointly shaped by pest density, crop stage, cultivar, natural enemies, pesticide use in surrounding commercial fields, scouting practice, and local cropping patterns (Li et al., 2012; Furlong et al., 2013; Li et al., 2016a, 2016b; Gurr et al., 2018; Machekano et al., 2019). Accordingly, the fitted temperature and wind-edge parameters should be interpreted as predictive model quantities rather than direct estimates of physiology or migration.

The standardized protocol supports consistent operational surveillance forecasting through a fixed per-field sampling effort of 100 inspected plants, a common formal scouting protocol, and untreated reference fields. Because representative fields could be replaced and cultivar, crop stage, natural-enemy abundance, surrounding-field management, and observer identity were not recorded, the resulting forecasts should be interpreted as predictions of the historical operational surveillance series rather than causal estimates of meteorological effects.

The practical contribution is a 1- to 4-week risk-screening window aligned with surveillance and integrated pest-management schedules. Current evidence supports use within jointly supervised station networks, and it shows that high-frequency meteorology should be encoded deliberately rather than appended as a flat feature block. Prospective studies should assess transfer to new stations and quantify effects on yield, pesticide use, and economic outcomes against local action thresholds.

An initial operational workflow would use TG-PestNet as a triage system: station- and horizon-specific forecasts would identify where additional scouting is warranted, and field observations of crop stage, pest density, natural enemies, and recent control actions would inform intervention decisions.

Management value should be assessed prospectively through decision-curve, cost-loss, and threshold analyses, because the consequences of false alarms and missed outbreaks differ by forecast horizon. At the present stage, TG-PestNet is best positioned as a regional risk-screening tool that triggers targeted scouting and field confirmation.

### 4.3 Scope and priorities for validation

The component audit supports a predictive rather than mechanistic interpretation. Variable-group attention was unrelated to exact Shapley values and nearly uniform. Base-temperature estimates showed no consistent direction or improvement in accuracy across the learning-rate sweep. Wind-edge effects were neither monotone nor stable across seeds, and the removal and sign-constrained variants had intervals spanning zero. These diagnostics define concrete targets for constrained-model development and external validation.

The auxiliary ZOIB head models the bounded, endpoint-inflated encoder branch. Its auxiliary-branch diagnostics showed modest calibration departures (**Supplementary Table 23**): the randomized probability integral transform departed from uniformity, with a mean per-seed Kolmogorov–Smirnov statistic of 0.086, while empirical coverage was 86.1% for the nominal 90% interval and 56.7% for the nominal 50% interval. Because the final logit-space gate combines the encoder mean with the history anchor without propagating a full distribution, these diagnostics do not describe the predictive distribution of the reported point forecast. A future probabilistic extension should train the final gated distribution directly and evaluate calibration, sharpness, and proper scoring rules (Gneiting et al., 2007).

The study’s scope defines five priorities for validation. First, architectures that accommodate asynchronous station records could recover 64% of the Huiyang archive and 61% of the Shantou archive currently excluded and support evaluation beyond four stations in one subtropical province. Second, larger, prospectively powered samples are needed to resolve effects near three percentage points and distinguish component contributions from optimization sensitivity. Third, broader generic-encoder benchmarks and prospectively defined rapid-warming and high-temperature regimes would test generality. Fourth, linking field-level survey sheets with crop phenology, host continuity, natural enemies, surrounding-field pesticide use, cultivar, management intensity, and within-field microclimate would permit reconstruction of station-week sampling and strengthen ecological interpretation. Fifth, the final gated output should be trained and evaluated as a coherent predictive distribution.

Transfer experiments show that unsupervised use at new stations is not yet supported: the error was 7%–62% higher than under joint supervision and did not reliably improve on persistence. Deployment at a new station should, therefore, begin with locally labeled calibration. Retrospective full-history comparisons suggest that performance also varied with the number of training origins, but training-set size remains confounded with test-year composition. The next decisive evaluation is prospective and timestamped, using the frozen model to issue weekly forecasts before the corresponding survey rounds and scoring them after each survey season.

Targeted follow-up experiments should compare literature-fixed thresholds, isolate the growth recursion, and train without the auxiliary ZOIB term. Larger training sets and designs that separate component effects from optimization sensitivity will improve precision for effects of this order.

Taken together, the study establishes a promising, auditable crop-protection forecaster and a clear validation agenda. Its strongest supported contribution is forecasting with a protected history pathway; thermal, attention, and spatial components remain testable hypotheses for future work.

## 5 Conclusions

TG-PestNet provides an auditable framework for 1- to 4-week forecasting of DBM infestation at monitored stations. In chronological tests, it achieved the lowest descriptive RMSE, MAE, and MAPE in every station–metric cell, beat persistence in all four year-ahead evaluations, and outperformed two input-matched generic encoders. These gains were directionally consistent, although station-specific intervals against the strongest tuned comparator included zero. Component audits identify the protected history anchor as the clearest contributor and show which thermal, attention, and spatial hypotheses require further testing. These results together support TG-PestNet as a promising regional forecasting tool and provide a clear pathway to prospective, timestamped validation across additional stations and management contexts.

## Data Availability

The raw data supporting the conclusions of this article will be made available by the authors, without undue reservation.
