# Supplementary material for: Multi-source temporal fusion and multi-station learning for early warning of diamondback moth infestation in Brassica crops
Source: Front Plant Sci. 2026 Oct 12;17:1953590. doi: 10.3389/fpls.2026.1953590 (PMC13696400; doi:10.3389/fpls.2026.1953590)
Supplement: Supplementary file 1 [file DataSheet1.pdf]

Supplementary Materials

Multi-source temporal fusion and multi-station learning for early warning of diamondback moth infestation in Brassica crops

Contents of the Supplementary Materials

Supplementary Methods SM1. Data provenance, file manifest, and preprocessing.

Supplementary Methods SM2. Full TG-PestNet implementation and parameter accounting.

Supplementary Methods SM3. Baseline grids, controls, ablation protocol, and statistical implementation.

Supplementary Methods SM4. Transfer, year-ahead, and early-history protocols.

Supplementary Methods SM5. Thermal-regime and wind-edge counterfactual protocols.

Tables S1–S8. Frozen configuration and parameter ledger (S1); complete test rankings (S2); flat-block penalties by family (S3); occlusion, Shapley values and group attention (S4); comparator-level Diebold–Mariano tests (S5); paired local baselines with and without weather (S6); capacity-matched versus bypassed ablations and block-length sensitivity (S7); notation (S8).

Tables S9–S15. Meteorological variable groups (S9); preprocessing corrections (S10); field-surveillance protocol and metadata availability (S11); year-ahead evaluation by season (S12); near-zero-variance screening of the 39 hourly variables (S13); chronological partition and development folds (S14); audit index by structured component (S15).

Tables S16–S23. Fitted history–encoder gate (S16); fitted exposure thresholds in physical units (S17); the 14 weekly variables of the flat block (S18); baseline hyperparameter candidates (S19); reported model by station and target-week temperature (S20); seed-level test RMSE (S21); physical-parameter learning-rate sweep (S22); auxiliary ZOIB calibration diagnostics (S23).

Tables S24–S28. Shuffled and noise representation controls (S24); raw-hourly encoders and the three estimates of the meteorological contribution (S25); archive coverage and the four-arm early-history study (S26); test RMSE by thermal regime (S27); wind edge-bias sweep and the sign-constrained variant (S28).

Figures S1–S3. Fitted anchor lag weights (S1); wind edge-bias counterfactual sweep (S2); flat-weather-block penalty by model family (S3).

Supplementary Methods

These sections carry the implementation detail that the main text summarizes. Section numbers refer to the revised manuscript.

SM1. Data provenance, file manifest, and preprocessing

SM1.1 Surveillance frame and recoverable metadata. The administrative monitoring area, rather than an unchanged physical field, constituted the longitudinal surveillance frame. Representative fields could be replaced within the same monitoring area, for example following crop turnover, while field selection and scouting continued under the same formal protocol. The authors received weekly station-level percentages rather than the underlying field-survey sheets, so the exact number of fields, survey rounds and plants contributing to each station-week could not be reconstructed from the supplied extract. Cultivar, exact transplanting date, crop stage, crop rotation and individual observer identifiers were also not systematically included, so these variables were neither standardized across the series nor available as model covariates. Throughout the manuscript the weekly value is interpreted as a provider-recorded operational surveillance measure rather than epidemiological incidence, a direct count of DBM abundance, or an estimate obtained from

permanently fixed physical fields. Supplementary Table S11 lists each protocol dimension and whether it is confirmed, partially recoverable or absent.

SM1.2 Written clarification and the early archive. According to written clarification from the data provider, the same formal surveillance protocol was applied throughout 2015–2022. That clarification covers 2015–2022 only. The protocol under which the earlier Huiyang and Shantou records were collected is not documented in the extract supplied to the authors, and the standard cited for the later period carries a 2009 designation, five years after the Huiyang archive begins. The original station archives begin on 23 February 2004 at Huiyang and 30 May 2005 at Shantou, whereas Shenzhen and Dongguan begin on 7 September 2015; this is why the joint analysis is restricted to the common Sunday-ended calendar and why the value of the discarded history is measured separately (SM4.3).

SM1.3 Meteorological source files. Hourly reanalysis meteorology was obtained from the HTQX meteorological data platform (https://htqx.cn/meteo), which distributes the ERA5 reanalysis produced by ECMWF and the Copernicus Climate Change Service. One annual comma-separated file was retrieved per station for the location labels 惠阳区 (Huiyang), 汕头市 (Shantou), 深圳市 (Shenzhen) and 东莞市 (Dongguan), covering 2015 to 2022 and downloaded on 15 June 2026; for the full-archive arm the same platform supplied the earlier annual files for Huiyang from 2004 and for Shantou from 2005, downloaded on the same date. Each record carries a timestamp in UTC+8 and 39 numeric variables, all of which enter the hourly encoder. The weather_code field follows WMO code 4677. In the frozen preprocessing it was retained as one raw numeric column and standardized with the continuous channels; it was not one-hot encoded or embedded. Eleven distinct codes occur in the study window (Supplementary Table S13). This implementation detail is reported for reproducibility, but numeric distance between codes is not interpreted as physical distance or as a continuous precipitation-intensity scale. Alternative categorical encodings were not tested, and transferability to products with a different code set remains unestablished. All 53 files, with their time spans, row and column counts and SHA-256 checksums, are listed in the source-file manifest released with the code. The 32 files covering the joint window carry 280,512 hourly records, of which the 231,168 falling in the joint study window are used; the 21 earlier files, covering Huiyang from 2004 to 2014 and Shantou from 2005 to 2014, carry a further 184,080 records and enter only the full-archive arm. A model input week must contain all 168 hourly observations; incomplete weeks were dropped and no imputation was performed.

SM1.4 Why six variables carry thresholds. Six variables receive differentiable soft thresholds: 2-m temperature, vapor-pressure deficit, precipitation, 10-m wind speed, 10-m wind gusts and shortwave radiation. These were selected a priori. For temperature, rainfall and wind the DBM-specific evidence is direct: temperature governs development, survival and reproduction, rainfall dislodges eggs and early instars, and wind governs flight and long-distance movement. For vapor-pressure deficit and shortwave radiation the evidence is indirect. Humidity affects DBM in interaction with temperature, host water deficit lowers DBM survival, reproduction and oviposition, and leaf-surface humidity is a filtered version of atmospheric humidity; solar UV-B alters host attractiveness and resistance to DBM, radiation drives leaf temperature above air temperature, and DBM flight is light-dependent. These two variables were therefore included as proxies for atmospheric drying power and for UV-B exposure, leaf-temperature excess and light-limited flight, not as variables with documented DBM thresholds. The supporting citations are given in the Introduction and Section 2.2 of the manuscript.

SM1.5 Standardization and leakage control. Standardization moments (per-variable mean and scale) were estimated from the hours of the fitting block of each stage — training hours during model selection, and training-plus-validation hours for the final refit — and applied unchanged to all later data. No test-period hour enters any moment: the last fitting week (index 270) precedes the first test forecast origin (index 274). Variables with a standard deviation below 10^{-6} receive unit scale. Raw temperature remains in degrees Celsius for degree-day calculation. All meteorological inputs end at the forecast origin; no realized future weather is included. No variance screening was applied to the reported model, which retains all 39 variables; the screening diagnostics are Supplementary Table S13 and a variant retrained on the cleaned set is Supplementary Table S10.

SM2. Full TG-PestNet implementation and parameter accounting

SM2.1 Hour-to-day summarization. For day d and variable v the encoder computes the mean, the standard deviation, K = 5 order statistics and an hour-of-day weighted mean across the 24 within-day hours. The order statistics are the sorted values at zero-based ranks (0, 6, 12, 17, 23), obtained as $r_k = \mathrm{round}(23 q_k)$ for q = (0.00, 0.25, 0.50, 0.75, 1.00) under round-half-to-even: the daily minimum,

the approximate lower quartile, the upper median, the approximate upper quartile and the daily maximum. The 24 hour-of-day attention weights are shared within the physical group of the variable, which allows thermal variables to emphasize a different part of the diurnal cycle from wind or moisture variables. With four exposure-count slots, which are identically zero for the 33 variables that carry no thresholds, each variable-day yields a 12-element summary ($f_{day} = 12$), and the weekly per-variable vector concatenates the attention-pooled and mean-pooled day summaries ($f_{week} = 24$).

SM2.2 Soft exposure counts and initialization. For the six threshold-enabled variables the encoder computes differentiable exposure counts; each learned threshold is paired with a positive sharpness parameter, so the count approaches a hard exceedance count as the sharpness decreases while remaining differentiable during training. Thresholds are optimized on the standardized scale and transformed back to physical units for reporting only. Four thresholds are parameterized per selected variable, initialized at (−1.5, −0.5, 0.5, 1.5) standardized units, with sharpness initialized at 0.41; the hour-of-day logits are initialized at zero, that is, exactly uniform after the softmax. The fitted values in physical units are Supplementary Table S17.

SM2.3 Pooling, group attention and the history projection. The seven daily vectors are pooled by content-dependent attention, which avoids assigning meaning to an arbitrary weekday label and allows a short extreme episode to receive more weight than uneventful days; the attention-pooled vector is concatenated with the unweighted weekly mean, which keeps a stable summary when the attention distribution becomes diffuse. The 39 variables are partitioned into the seven groups of Supplementary Table S9, each group is projected independently, and a second attention mechanism combines the group representations into the weekly station embedding. The infestation-history projection is a two-layer map with $W_1 \in \mathbb{R}^{64\times2}$ and $W_2 \in \mathbb{R}^{64\times64}$ applied to the endpoint-clipped observed value and its logit at all $L = 8$ lookback weeks; its output is added to the normalized weekly station embedding without renormalization, before the station embeddings of the cross-station layer. It contains 4,352 parameters and belongs to the backbone learning-rate group.

SM2.4 Thermal-time encoder. Hourly degree-day contributions are computed with a smooth rectifier of fixed sharpness $\kappa = 4$, which provides a differentiable approximation to positive thermal accumulation. The base temperature is constrained to a 5–15 °C search interval that brackets published developmental thresholds for DBM and is initialized at 8 °C. Cumulative degree-days define the relative displacement between any two lookback weeks, and that displacement enters the temporal self-attention logits through a learned head-specific bias. The production encoder uses two pre-normalized Transformer blocks, model width 64, four attention heads, GELU feed-forward layers and dropout 0.10. Fixed scales are $dd_{scale} = 100$ °C·day, $w_{scale} = 10$ m s^{-1} and $r_{scale} = 2.0$.

SM2.5 Cross-station coupling. All stations share the hourly and thermal-time encoders. At each week the station representations interact through directed attention: for an edge from source station i to receiving station j the model projects the weekly 100-m wind vector onto the geographic bearing from i to j, and the projection, the normalized station distance and an exponential distance term are passed to a head-specific multilayer perceptron to form an additive attention bias. The receiving station supplies the query, so $i \rightarrow j$ and $j \rightarrow i$ can differ. The perceptron is unconstrained, so neither the sign nor the monotonicity of the learned bias is guaranteed: a positive downwind projection need not increase compatibility, a larger distance need not reduce it, and setting the self-edge projection to zero does not force the self-edge bias itself to zero.

SM2.6 Decoder, anchor, auxiliary head and objective. The decoder predicts a bounded latent increment for each forecast step from the station context, the target-week seasonal phase and a horizon embedding. Target week-of-year is represented by a Fourier basis with harmonic index q; the frozen preprocessing used the ISO week number with a denominator of 52, a 52-week approximation rather than an exact solar-year phase, so rare ISO week 53 values are not strictly periodic. Retraining with an exact day-of-year phase changed macro RMSE by +0.10% (Supplementary Table S10). A separate positivity constraint keeps the density-style scalar β positive; because the state is expressed in logit space the recursion remains compatible with a bounded rate after inverse transformation. The history anchor is a station- and horizon-specific convex combination of the eight lookback values whose 128 lag logits are initialized with logit 2 on the most recent lag and 0 elsewhere, giving an initial weight of approximately 0.51 on the most recent week. The auxiliary zero-one-inflated Beta head takes the interior Beta mean from the latent state and determines precision and endpoint inflation from separate outputs; its mixture mean is the encoder-branch quantity that the gate blends, not the reported forecast. The objective is the masked negative log likelihood of that auxiliary distribution plus $\lambda_1 = 2.0$ times the mean absolute error and $\lambda_2 = 1.0$ times a stabilized relative error on the final gated point forecast, with $\varepsilon_r = 0.05$ in the relative-error denominator and $\varepsilon_g = 10^{-4}$ used for endpoint clipping before every logit operation.

SM2.7 Optimization and the frozen configuration. Optimization used AdamW with three parameter groups. The neural backbone, which includes the infestation-history projection, receives the base learning rate of 3×10^{-4} with weight decay 1×10^{-4}. The base temperature and β receive one tenth of that rate and no weight decay, which limits abrupt movement away from their initial values. The 128 station-horizon lag logits and 16 gate logits receive twenty times the base rate and no weight decay, because they otherwise move slowly in the small-sample regime. Training uses cosine annealing to 1×10^{-5}, gradient-norm clipping at 1.0, a maximum of 80 epochs with patience 15, deterministic seeding and a batch of 8 joint origins. After the configuration was fixed the model was refitted on the combined training and validation data for 12 epochs under the minimum-of-mean-curve rule. All frozen settings are Supplementary Table S1, Panel A.

SM2.8 Parameter accounting. The frozen model has 246,757 trainable parameters in 101 tensors, each assigned to exactly one optimizer group: 97 tensors and 246,611 parameters in the backbone group; two tensors in the physical group; and the 128 lag logits and 16 gate logits, 144 parameters in two tensors, in the anchor group. Panel B of Supplementary Table S1 reconciles the module totals as 91,942 + 100,325 + 17,284 + 32,838 + 4,352 + 16 = 246,757 and Panel C lists every tensor with its name, shape, element count and gradient status. One tensor, the final bias of the variable-group scorer, is structurally inert: it adds the same constant to all seven group logits before the softmax and therefore receives an exactly zero gradient. Independent re-evaluation of all ten final checkpoints reproduces the stored test predictions to float32 round-off, with a maximum absolute difference of 1.5×10^{-5} percentage points, and an automated test asserts the checkpoint hashes, the parameter total, the tensor count and the module totals.

SM3. Baseline grids, controls, ablation protocol, and statistical implementation

SM3.1 History-only input set. All baseline families receive the history-only input set: the eight most recent weekly infested-plant values; 14 summary features of that window (last value, mean, standard deviation, minimum, maximum, range, slope, absolute slope, last difference, mean and maximum absolute difference, share of zero weeks, share of weeks at or below 5%, and last value minus mean); sine–cosine encodings of the ISO week of each of the four target weeks, which are known at the forecast origin; and four station indicators — 34 columns in the global scope and 30 in the local scope, which omits the station indicators. No meteorological input enters this set.

SM3.2 Candidate grids and selected configurations. Candidate grids were 4 configurations for Ridge, Random Forest, Extra Trees, Histogram Gradient Boosting, N-HiTS-small, NLinear, MLP and GRU, 16 for TemporalConvNet and 18 for SVR, LSTM and Transformer; the first nine families were selected on the three development folds and the last three on training-to-validation macro RMSE. Every candidate and its score is in Supplementary Table S19. The selected configurations were an RBF-kernel SVR with C = 1.0, epsilon = 0.2 and gamma = 0.01; an LSTM with 16 hidden units, dropout 0.2, learning rate 1e-3 and weight decay 1e-4; and a Transformer with 32 hidden units and the same dropout, learning rate and weight decay. The selected LSTM and Transformer were refitted on training plus validation for 345 and 164 epochs respectively. Models are summarized over seeds 40–49, with metrics computed per seed before averaging. The four families whose grids are smallest and whose accuracy is most tuning-sensitive — N-HiTS-small, NLinear, MLP and GRU — are never the lowest-error comparator in any station–metric cell.

SM3.3 Paired history-plus-weather condition. The history-plus-weather condition adds 168 leakage-safe columns derived solely from meteorology observed before the forecast origin: 14 weekly variables, listed with their source hourly variable and transform in Supplementary Table S18, entering both as an eight-week lagged sequence (112 columns) and as four window statistics per variable — the means of lookback weeks 1–4, 5–6 and 7–8 and the eight-week maximum (56 columns) — for 198 columns in total at local scope. The two conditions share the same 1,056 sample-by-station-by-horizon test keys, identical observations and the same seed sets. Hyperparameters were searched separately for each feature condition, on the grounds that a configuration tuned for the 30 history columns cannot be assumed appropriate for 198.

SM3.4 Metrics. Performance is evaluated on the original percentage scale. For station s and horizon k, RMSE is computed over the 66 test origins and then averaged equally across the four horizons; MAE and MAPE are pooled over the station's 264 station–horizon test rows. Exact zeros are excluded from MAPE, which avoids undefined ratios but does not remove the metric's known instability at small

positive observations. Each metric is computed independently for every seed and then averaged, and uncertainty is reported as the standard deviation of seed-level metrics.

SM3.5 Inference. Forecast-loss differences were assessed using the Diebold–Mariano test on origin-level squared-error differentials, TG-PestNet minus comparator, so that negative values favor TG-PestNet. Overlapping multi-step targets induce serial dependence; variance is estimated with the Newey–West heteroskedasticity- and autocorrelation-consistent estimator using $k - 1$ Bartlett lags and the Harvey–Leybourne–Newbold small-sample correction, and tests are read against a t distribution with 65 degrees of freedom. All 12 baseline families were tested at each station and no single baseline was designated in advance as a confirmatory reference, so nominal two-sided p values are accompanied by Holm adjustment within each station across the 12 comparator tests. Every 95% interval on a relative difference comes from a hierarchical paired moving-block bootstrap that resamples forecast origins in blocks and draws the seed label jointly for both arms under the registered same-label pairing convention; this is an analysis convention rather than a claim that identical seed numbers induce identical stochastic trajectories across architectures; the primary block length is eight weeks with 2,000 replicates, and four- and twelve-week block lengths are reported as a sensitivity check in Supplementary Table S7.

SM3.6 Retraining-ablation protocol. Ten structural variants and one optimization-sensitivity control were retrained from scratch. Every variant shares the frozen configuration, the chronological split with 195 training, 66 validation and 66 test joint forecast origins, the same three tuning seeds (37–39) for selecting the refit epoch on validation macro RMSE, and the same ten final seeds (40–49) refit on training plus validation. No hyperparameter search was repeated for any variant, so a difference between rows can arise only from the switch named in that row, and each switch is a field of the frozen model configuration whose default reproduces the reported model exactly. The variants are of two kinds. In the first, no module is bypassed and at most a few scalars are frozen: cross-station exchange is removed by masking the station attention to its diagonal; wind by zeroing the projection term while retaining both distance terms; the history anchor and the encoder branch by freezing the sixteen gate logits at $g = 1$ and $g = 0$ respectively; degree-day relative attention by replacing it with calendar-week relative attention through the same bias network; the base temperature by freezing its single scalar at the 8 °C initialization; and the exposure-count channels by zeroing them at the input while leaving the mean, standard deviation, order statistics and every downstream projection dimension unchanged. These seven hold the active parameter count at 246,757 and constitute the multiplicity family, within which Holm correction is applied. In the second kind a module is removed from the forward pass, so capacity falls together with information: bypassing the infestation-history projection leaves 242,405 active parameters, bypassing the cross-station layer leaves 229,473, and removing the exposure-count features leaves 226,785; these are reported alongside the family but are not corrected with it. The optimization-sensitivity control is not a hypothesis and is not corrected with them.

SM3.7 Representation controls and raw-hourly encoders. Three representative families were pre-selected by model class and by validation behavior under the history-only condition — Ridge for linear, Extra Trees for tree-based and TemporalConvNet for deep sequence models; the criterion was recorded qualitatively rather than as a numeric rule, and no test result was consulted. Two control conditions match the width of the real block exactly. The shuffled condition permutes the weather block across forecast origins independently within each station and split, preserving every marginal distribution, the cross-variable structure and the within-sample temporal structure while destroying only the correspondence between weather and target. The noise condition replaces the block with independent standard normal draws, which is distribution-matched because the block is already standardized on training statistics. Each condition was drawn fifty times and every draw re-selected hyperparameters under the same grid as the real block, so the control distribution includes selection variability as well as sampling variability. Significance is a one-sided permutation p value, $(b + 1)/(n + 1)$, where b counts the control draws whose penalty is no larger than the real block's; the smallest attainable value is 0.09 at ten draws and 0.02 at fifty. Both the local and the global scope are run. The two raw-hourly encoders consume the same hourly tensor as TG-PestNet and contain none of its structure: no learned exposure thresholds, no thermal-time encoder, no wind-informed station graph, no bounded growth recursion, no protected history anchor or gate and no ZOIB head. The first applies dilated causal convolutions across the full 1,344-hour lookback window; the second embeds each 24-hour block into a token, giving 56 daily tokens, and applies self-attention. Each station is encoded by the same shared weights and station identity enters the readout head as an indicator. The readout head receives the pooled encoder output together with the complete history-only column set — the eight weekly lags and their logits, the 14 summary features, the eight target-week calendar values and the four station indicators, 42 non-weather columns in all — which is more engineered history than TG-PestNet itself uses. Capacity is held to within ±10% of TG-PestNet's 246,757 parameters, that is 222,081 to 271,433. Selection used 16 candidates across the three development folds and three tuning seeds, 144 fits per architecture, and the

selected configuration was refit on ten final seeds. A within-architecture control repeats each architecture with the hourly tensor zeroed, leaving the encoder and the readout head unchanged.

SM4. Transfer, year-ahead, and early-history protocols

SM4.1 Leave-one-station-out transfer. Each station in turn is withheld: its labels are excluded from the training loss and from the epoch-selection criterion, while its hourly weather and observed infestation history remain inputs. The withheld station therefore receives no gradient at its own station-specific parameters, and its four gate logits and thirty-two anchor lag logits remain exactly at initialization (a gate of 0.5 and the initial lag profile); its station embedding is trained only through the cross-station layer. Results are compared with the jointly supervised model from the same harness and with the LastValue and Mean8 anchors at the withheld station, over seeds 40–49. The configuration was not re-selected.

SM4.2 Year-ahead evaluation. Each calendar year Y from 2018 to 2021 is held out in turn; the preceding complete year is the validation block used to select the refit epoch, all earlier origins form the training block, and the four-week embargo, which removes three intervening origins, separates the blocks, giving 59, 111, 164 and 213 training origins. The weather scaler, the epoch choice and all fits use only data preceding Y, and weights are not updated within Y. At each origin the model uses the infestation history observed up to that origin. Comparators are the history-only TemporalConvNet re-tuned on pre-Y data for each Y, LastValue, Mean8 and the no-cross-station variant. Errors are computed per year, station and horizon and averaged across years with equal weight; Diebold–Mariano tests are Holm-corrected across comparators within each year. The same forecasts are re-aggregated by the target week's meteorological season in Supplementary Table S12. The 2022 portion of the calendar holds only eleven joint origins and is not reported as a separate year.

SM4.3 Discarded early history. Four arms were compared at Huiyang and Shantou. Arm A is the reported joint model. Arm A′ is the joint model with cross-station attention masked to self-edges, the capacity-matched variant of SM3.6, whose fitted checkpoints are reused rather than refitted. Arm B is a single-station model: the frozen configuration with S = 1, so that cross-station attention reduces to the self-edge and the station embeddings, the history–encoder gate logits and the anchor lag logits exist for one station only, giving 246,457 parameters against 246,757; it is fitted on the common-calendar training block. Arm C is the same single-station model with its training block extended back to the first admissible origin of the station's archive, the week ending 18 April 2004 at Huiyang and the week ending 24 July 2005 at Shantou, giving 797 and 731 training origins against 195. The validation and test blocks, the four-week embargoes, the epoch-selection rule with tuning seeds 37–39 and the ten final seeds 40–49 are common to every arm, and the weather scaler of each arm is fitted on its own training block. Hourly ERA5 for the pre-2015 origins came from the same platform and is listed in the same manifest (21 files). The Shenzhen archive contains no weeks beyond the common calendar, so its B and C arms use identical data and were run as a determinism check; Dongguan's archive extends five weeks beyond the calendar's end, all of them after 10 April 2022, adds no training origins and was not run. Four contrasts are reported per station as relative differences in test RMSE, (RMSE of the first-named arm − RMSE of the second)/RMSE of the second: C versus B, A versus C, and the decomposition A versus A′ and A′ versus B. Holm correction is applied within the family of eight station-by-contrast tests, and the Shenzhen check is outside the family. No hyperparameter search was repeated: the configuration was selected at 195 joint training origins and is reused unchanged at 797 and 731, so the full-archive arm may be capacity-limited, which biases C versus B toward finding no benefit.

SM5. Thermal-regime and wind-edge counterfactual protocols

SM5.1 Regime stratification. Test errors were re-aggregated retrospectively by the thermal conditions of the target week. The strata are computed from the target week's realized meteorology, which no model received at forecast time, so they are a description of where errors fall and not model inputs. The unit is the station-by-horizon target-week forecast, 1,056 per seed, and the three stratifications partition the same forecasts: target-week mean temperature, target-week maximum hourly temperature, and target-week accumulated degree-days above 8 °C with tercile boundaries fixed on the training era within each station. A stratum is reported only when its forecasts come from at least 20 distinct origins; the 35–38 °C band failed that rule with 16 origins and was merged into 30–35 °C, contributing 20 of 472 forecasts. Comparators are the stored test predictions of the two thermal ablation variants, the raw-hourly encoder and the LastValue anchor; nothing was retrained.

SM5.2 Wind edge-bias counterfactual sweep. For each ordered station pair $i \rightarrow j$ and each of the ten frozen seeds, the normalized distance is held at its true value and the downwind projection is replaced by each of 41 equally spaced values between the 5th and 95th percentiles of that pair's training-era projections, with every other input unchanged. Because the sweep intervenes on the projection fed to the edge bias and not on the wind variables in the hourly tensor, the queries and keys of the cross-station attention are unaffected, and the realized attention weight is recomputed from the queries and keys captured from the frozen checkpoint. Both the bias and the realized weight are averaged over the four attention heads and the eight lookback weeks of the first sixteen test origins — sixteen of the sixty-six, a limitation of the analysis rather than of the model — giving one response curve per pair and seed, 120 curves in all. A curve is monotone increasing if the realized weight strictly increases at all 40 adjacent steps and non-increasing if it never strictly increases; the violation fraction is the share of the 40 steps on which the weight does not increase; and the downwind-minus-upwind difference is the mean weight over the sweep points with positive projection minus the mean over those with negative projection. No retraining is involved.

SM5.3 Sign-constrained variant. The perceptron in the edge bias is replaced by a sign-constrained form, $\mathrm{softplus}(a_n)\cdot\rho_{i\rightarrow j,l}/w_{scale} - \mathrm{softplus}(c_n)\cdot D_{ij} + d_n$ per head, so that the bias increases with downwind projection and decreases with distance by construction. It is trained and analyzed under the protocol of SM3.6: the same split, tuning seeds 37–39, final seeds 40–49, no repeated search and the same bootstrap. The three scalars per head replace the 260-parameter perceptron in the forward pass; the perceptron remains allocated but receives no gradient, so the registered parameter count rises by twelve to 246,769 while the edge bias itself falls from 260 free parameters to twelve. Because capacity is not matched, the variant is reported outside the multiplicity family.

Table S1. Frozen model configuration and parameter-accounting audit: hyperparameters of the model (Panel A) and the trainable-parameter ledger generated from model.named_parameters() of the frozen checkpoint, reconciled by module (Panel B) and listed tensor by tensor (Panel C).

Panel A. Frozen model configuration

Setting	Frozen value
Lookback / horizons	8 completed weeks / 1–4 weeks ahead; direct multi-horizon
Model width / heads / thermal blocks	64 / 4 / 2
Soft thresholds	4 per selected variable; 6 variables
Fourier order	3; harmonic index q; ISO-week/52 approximation
Dropout	0.10
Base-temperature range	5–15 °C; initialized at 8 °C; parameterized as ρ_T with logit initialization 0.3
**History branch**	**128 station-horizon-lag logits plus 16 gate logits; logit blend; initial gate 0.5; lag logits initialized with logit 2 on the most recent lag (weight ≈ 0.51), gate logits initialized at 0**
Loss	**Auxiliary encoder-branch ZOIB NLL + 2.0 MAE + 1.0 stabilized relative error on the final point forecast**
Optimizer	AdamW; base LR 3×10^{-4}; weight decay 1×10^{-4}
LR multipliers	Physical parameters ×0.1; anchor/gates ×20; backbone ×1
Schedule / clipping	Cosine to 1×10^{-5}; gradient clip 1.0
Epochs / early stopping / final refit	Maximum 80; patience 15; minimum-of-mean-curve rule; frozen training+validation refit = 12 epochs
Batch size	8 joint origins (4 stations × 4 horizons per origin)
Parameters	**246,757 in 101 tensors, all trainable; reconciled in Panel B**
Seeds	37–39 for selection; 40–49 for final test
Within-day order statistics	K = 5; q = (0.00, 0.25, 0.50, 0.75, 1.00); zero-based ranks (0, 6, 12, 17, 23) under round-half-to-even
Day- and week-level feature widths	f_day = 12 (mean, SD, 5 order statistics, hour-of-day weighted mean, 4 exposure-count slots); f_week = 24
Endpoint clipping	$\varepsilon_g = 10^{-4}$ at all three logit operations (history projection, decoder initialization, gate); relative-error floor $\varepsilon_r = 0.05$
Fixed scales	dd_scale = 100 °C·day; w_scale = 10 m s^{-1}; r_scale = 2.0; degree-day smooth-rectifier sharpness κ = 4
Exposure-threshold initialization	(−1.5, −0.5, 0.5, 1.5) standardized units per selected variable; sharpness initialized at 0.41
Hour-of-day initialization	Logits at zero, i.e. exactly uniform after the softmax
Standardization fitting scope	Training hours for the selection runs; training-plus-validation hours for the final refit. Last fitting week index 270; first test forecast origin index 274
Standardization variance guard	Scale set to 1 when the fitted standard deviation is below 10^{-6}
Variance screening	None applied in the frozen model; all 39 variables enter the encoder
Complete-week rule	A model input week must contain all 168 hourly observations; incomplete weeks are dropped and no imputation is performed
Masked-target rule	Non-finite targets are masked and excluded from the loss and from every reported metric
Structurally inert parameter	The final bias of the variable-group scorer (1 parameter) adds the same constant to all seven group logits before the softmax and receives an exactly zero gradient

Panel B. Parameter-accounting audit

Component (manuscript section; code attribute)	Parameters
Threshold-exposure hourly encoder (Section 2.3.1; m1)	91,942
Thermal-time encoder (Section 2.3.2; m2)	100,325
Wind-informed cross-station coupling (Section 2.3.2; m3)	17,284
Latent-state decoder and auxiliary ZOIB head, including the 128 lag logits (Section 2.3.3; m4)	32,838
**Infestation-history projection (Section 2.3.1; pest_history_proj)**	**4,352**
**Station-horizon gate logits (Section 2.3.3; logit_blend)**	**16**
**Total, generated from model.named_parameters() (101 tensors, all trainable)**	**246,757**

Panel C. Per-tensor ledger of the frozen checkpoint, all 101 tensors.

Parameter tensor (model.named_parameters())	Shape	Elements	requires_grad
logit_blend	4 × 4	16	True
m1.hour_logits	7 × 24	168	True
m1.thresh.tau	6 × 4	24	True
m1.thresh.log_s	6 × 4	24	True
m1.day_score.weight	1 × 12	12	True
m1.day_score.bias	1	1	True
m1.group_proj.0.0.weight	64 × 72	4,608	True
m1.group_proj.0.0.bias	64	64	True
m1.group_proj.0.3.weight	64 × 64	4,096	True
m1.group_proj.0.3.bias	64	64	True
m1.group_proj.1.0.weight	64 × 120	7,680	True
m1.group_proj.1.0.bias	64	64	True
m1.group_proj.1.3.weight	64 × 64	4,096	True
m1.group_proj.1.3.bias	64	64	True
m1.group_proj.2.0.weight	64 × 120	7,680	True
m1.group_proj.2.0.bias	64	64	True
m1.group_proj.2.3.weight	64 × 64	4,096	True
m1.group_proj.2.3.bias	64	64	True
m1.group_proj.3.0.weight	64 × 144	9,216	True
m1.group_proj.3.0.bias	64	64	True
m1.group_proj.3.3.weight	64 × 64	4,096	True
m1.group_proj.3.3.bias	64	64	True
m1.group_proj.4.0.weight	64 × 120	7,680	True
m1.group_proj.4.0.bias	64	64	True
m1.group_proj.4.3.weight	64 × 64	4,096	True
m1.group_proj.4.3.bias	64	64	True
m1.group_proj.5.0.weight	64 × 192	12,288	True
m1.group_proj.5.0.bias	64	64	True

Parameter tensor (model.named_parameters())	Shape	Elements	requires_grad
m1.group_proj.5.3.weight	64 × 64	4,096	True
m1.group_proj.5.3.bias	64	64	True
m1.group_proj.6.0.weight	64 × 168	10,752	True
m1.group_proj.6.0.bias	64	64	True
m1.group_proj.6.3.weight	64 × 64	4,096	True
m1.group_proj.6.3.bias	64	64	True
m1.group_attn.0.weight	32 × 64	2,048	True
m1.group_attn.0.bias	32	32	True
m1.group_attn.2.weight	1 × 32	32	True
m1.group_attn.2.bias	1	1	True
m1.norm.weight	64	64	True
m1.norm.bias	64	64	True
pest_history_proj.0.weight	64 × 2	128	True
pest_history_proj.0.bias	64	64	True
pest_history_proj.2.weight	64 × 64	4,096	True
pest_history_proj.2.bias	64	64	True
m2.tbase_raw	scalar	1	True
m2.bias_mlp.0.weight	32 × 2	64	True
m2.bias_mlp.0.bias	32	32	True
m2.bias_mlp.2.weight	4 × 32	128	True
m2.bias_mlp.2.bias	4	4	True
m2.blocks.0.ln1.weight	64	64	True
m2.blocks.0.ln1.bias	64	64	True
m2.blocks.0.ln2.weight	64	64	True
m2.blocks.0.ln2.bias	64	64	True
m2.blocks.0.qkv.weight	192 × 64	12,288	True
m2.blocks.0.qkv.bias	192	192	True
m2.blocks.0.out.weight	64 × 64	4,096	True
m2.blocks.0.out.bias	64	64	True
m2.blocks.0.ff.0.weight	256 × 64	16,384	True
m2.blocks.0.ff.0.bias	256	256	True
m2.blocks.0.ff.3.weight	64 × 256	16,384	True
m2.blocks.0.ff.3.bias	64	64	True
m2.blocks.1.ln1.weight	64	64	True
m2.blocks.1.ln1.bias	64	64	True
m2.blocks.1.ln2.weight	64	64	True
m2.blocks.1.ln2.bias	64	64	True
m2.blocks.1.qkv.weight	192 × 64	12,288	True
m2.blocks.1.qkv.bias	192	192	True
m2.blocks.1.out.weight	64 × 64	4,096	True
m2.blocks.1.out.bias	64	64	True
m2.blocks.1.ff.0.weight	256 × 64	16,384	True
m2.blocks.1.ff.0.bias	256	256	True
m2.blocks.1.ff.3.weight	64 × 256	16,384	True
m2.blocks.1.ff.3.bias	64	64	True
m2.norm.weight	64	64	True
m2.norm.bias	64	64	True
m3.station_emb.weight	4 × 64	256	True
m3.edge_mlp.0.weight	32 × 3	96	True
m3.edge_mlp.0.bias	32	32	True
m3.edge_mlp.2.weight	4 × 32	128	True
m3.edge_mlp.2.bias	4	4	True
m3.ln.weight	64	64	True
m3.ln.bias	64	64	True
m3.qkv.weight	192 × 64	12,288	True
m3.qkv.bias	192	192	True
m3.out.weight	64 × 64	4,096	True
m3.out.bias	64	64	True
m4.log_beta	scalar	1	True
m4.anchor_weight	4 × 4 × 8	128	True
m4.h_emb.weight	4 × 16	64	True
m4.rate_net.0.weight	128 × 86	11,008	True
m4.rate_net.0.bias	128	128	True
m4.rate_net.3.weight	1 × 128	128	True
m4.rate_net.3.bias	1	1	True
m4.phi_net.0.weight	128 × 80	10,240	True
m4.phi_net.0.bias	128	128	True
m4.phi_net.2.weight	1 × 128	128	True
m4.phi_net.2.bias	1	1	True
m4.mix_net.0.weight	128 × 81	10,368	True
m4.mix_net.0.bias	128	128	True
m4.mix_net.2.weight	3 × 128	384	True
m4.mix_net.2.bias	3	3	True
**Total, 101 tensors**	**—**	**246,757**	**All trainable**

Table S2. Complete test model rankings for all four stations. TG-PestNet and baseline values are mean ± standard deviation across seeds 40–49; deterministic baselines are reported once.

Station	Model	RMSE	MAE	MAPE (%)
Huiyang	TG-PestNet (proposed)	10.749 ± 0.417	7.352 ± 0.375	53.095 ± 1.825
	TemporalConvNet	10.834 ± 0.067	7.689 ± 0.078	71.592 ± 2.279
	LSTM	10.837 ± 0.129	7.779 ± 0.139	74.816 ± 2.946
	Transformer	10.920 ± 0.121	7.736 ± 0.116	71.370 ± 3.845
	N-HiTS-small	11.183 ± 0.179	8.127 ± 0.217	81.250 ± 5.990
	Ridge	11.305	7.939	70.183
	Random Forest	11.377 ± 0.028	8.029 ± 0.030	67.324 ± 0.557
	Extra Trees	11.456 ± 0.033	7.943 ± 0.031	66.075 ± 0.356
	SVR	11.466	8.422	76.620
	Raw-hourly TCN, weather off	11.762 ± 0.053	7.951 ± 0.038	60.806 ± 1.191
	NLinear	11.927 ± 0.816	9.161 ± 0.675	95.061 ± 17.860
	Histogram Gradient Boosting	12.230	8.495	70.223
	GRU	12.482 ± 0.858	9.799 ± 0.883	107.441 ± 21.487
	MLP	13.422 ± 1.581	10.459 ± 1.183	108.509 ± 17.026
Shantou	TG-PestNet (proposed)	3.032 ± 0.192	2.226 ± 0.123	14.240 ± 0.957
	Extra Trees	3.329 ± 0.018	2.586 ± 0.019	16.132 ± 0.107
	Ridge	3.402	2.589	16.018
	SVR	3.426	2.579	16.338
	Histogram Gradient Boosting	3.601	2.761	16.614
	Transformer	3.634 ± 0.169	2.901 ± 0.185	17.458 ± 1.054
	LSTM	3.704 ± 0.182	2.931 ± 0.193	17.988 ± 1.005
	Random Forest	3.733 ± 0.032	3.010 ± 0.026	18.045 ± 0.134
	TemporalConvNet	3.758 ± 0.157	3.039 ± 0.175	18.197 ± 0.967
	N-HiTS-small	4.829 ± 0.326	4.107 ± 0.295	23.420 ± 1.775
	Raw-hourly TCN, weather off	4.873 ± 0.247	3.971 ± 0.216	23.618 ± 1.150
	GRU	6.187 ± 1.423	5.276 ± 1.433	29.203 ± 7.408
	NLinear	6.692 ± 1.109	5.665 ± 1.060	30.737 ± 5.744
	MLP	8.298 ± 2.190	7.262 ± 2.223	39.512 ± 11.005
Shenzhen	TG-PestNet (proposed)	6.407 ± 0.150	4.378 ± 0.209	108.348 ± 7.276
	Raw-hourly TCN, weather off	6.995 ± 0.082	4.980 ± 0.100	148.708 ± 4.543
	SVR	7.241	5.616	151.647
	LSTM	9.208 ± 0.582	7.660 ± 0.643	224.997 ± 26.661
	Transformer	9.351 ± 0.551	7.857 ± 0.607	233.621 ± 19.383
	TemporalConvNet	9.369 ± 0.432	7.730 ± 0.484	235.274 ± 14.841
	Histogram Gradient Boosting	9.577	8.037	229.170
	Ridge	9.870	8.388	253.434
	Extra Trees	9.923 ± 0.049	8.420 ± 0.047	253.061 ± 1.678
	Random Forest	10.893 ± 0.109	9.419 ± 0.096	260.928 ± 2.562
	N-HiTS-small	11.571 ± 0.772	9.630 ± 0.757	296.547 ± 22.909
	MLP	14.277 ± 2.435	12.270 ± 2.735	338.179 ± 99.434
	NLinear	14.499 ± 2.604	12.516 ± 2.754	362.904 ± 80.610
	GRU	14.566 ± 1.980	12.420 ± 2.125	366.528 ± 74.525
Dongguan	TG-PestNet (proposed)	5.395 ± 0.284	4.052 ± 0.309	80.777 ± 7.917
	Raw-hourly TCN, weather off	5.714 ± 0.080	4.266 ± 0.122	81.324 ± 3.110
	SVR	6.164	4.754	97.633
	Random Forest	6.625 ± 0.049	5.300 ± 0.049	107.241 ± 0.862
	Transformer	7.050 ± 0.271	5.704 ± 0.283	111.533 ± 5.916
	Extra Trees	7.101 ± 0.050	5.955 ± 0.049	121.628 ± 0.913
	LSTM	7.122 ± 0.252	5.779 ± 0.285	113.485 ± 6.863
	Ridge	7.291	5.785	112.138
	TemporalConvNet	7.470 ± 0.268	6.055 ± 0.289	117.059 ± 6.180
	Histogram Gradient Boosting	8.670	6.517	123.918
	N-HiTS-small	9.117 ± 0.454	7.602 ± 0.440	145.461 ± 9.022
	MLP	12.163 ± 2.284	10.562 ± 2.243	192.731 ± 49.949
	GRU	12.586 ± 1.756	11.063 ± 1.862	207.377 ± 37.809
	NLinear	12.651 ± 1.919	11.112 ± 1.924	206.886 ± 36.013

Table S3. Median station-RMSE change after adding the 168-column weather block (history-plus-weather versus history-only), by model family. These are the exact values underlying Supplementary Figure S3.

Model family	Median RMSE change (%)	Primary handling of the 168-column block
Histogram Gradient Boosting	+2.0	Tree-based selection and shrinkage
Ridge	+12.0	Linear shrinkage
Extra Trees	+13.0	Bagging and feature selection
Random Forest	+13.8	Bagging and feature selection
SVR	+17.0	Kernel regularization
LSTM	+17.9	Gradient descent on flat block
GRU	+25.3	Gradient descent on flat block
Transformer	+33.3	Gradient descent on flat block
NLinear	+33.9	Gradient descent on flat block
N-HiTS-small	+55.8	Gradient descent on flat block
TemporalConvNet	+56.6	Gradient descent on flat block
MLP	+57.1	Gradient descent on flat block

Table S4. Variable-group occlusion, exact Shapley values and group attention weights, with seed-averaged ranks.

Variable group	Occlusion Δ RMSE (%)	p	Shapley value	p	Attention weight	Attention rank
Radiation	+1.18	0.033	+0.1361	0.056	0.1471	2
Wind	+0.87	0.007	+0.1180	0.007	0.1417	5
Pressure/cloud	+0.99	<0.001	+0.1495	<0.001	0.1438	4
Precipitation	+0.07	0.442	+0.0110	0.289	0.1446	3
Moisture	-0.13	0.725	+0.0442	0.250	0.1345	6
Soil	+0.83	0.156	+0.0408	0.486	0.1549	1
Thermal	+0.60	0.100	+0.0988	0.014	0.1334	7

Note. Positive occlusion values indicate higher error when a group is replaced by its test-tensor mean. Attention ranks are not interpreted as causal importance.

Table S5. Comparator-level Diebold–Mariano tests by station under the primary history-only protocol. Loss differential = TG-PestNet squared error minus comparator squared error; negative values favor TG-PestNet. Variance uses the Newey–West HAC estimator with the Harvey–Leybourne–Newbold small-sample adjustment. Comparator names are reported without inconsistent local/global tags; fitting details are defined in Section 2.4.1. The thirteenth comparator of Table S2, the raw-hourly TemporalConvNet with its weather input switched off, is not listed here: it was built after this table was generated, and it is instead tested against TG-PestNet by the paired block bootstrap of Section 2.4.1, which is the primary test for relative differences in this study. Its macro RMSE is 14.7% above TG-PestNet's (95% CI [+3.9, +21.8]%, Holm-adjusted p = 0.006 within the family of Supplementary Table S25).

Station	Comparator	Mean loss differential	DM statistic	p-value
Huiyang	MLP	-92.814	-3.245	0.0019
	GRU	-100.005	-2.969	0.0042
	NLinear	-88.128	-2.393	0.0196
	N-HiTS-small	-64.527	-1.736	0.0874
	TemporalConvNet	-57.262	-1.669	0.0999
	Histogram Gradient Boosting	-22.053	-1.321	0.1912
	Random Forest	-37.321	-1.181	0.2421
	SVR	-16.503	-0.936	0.3526
	Ridge	-8.079	-0.800	0.4265
	Extra Trees	-10.325	-0.485	0.6295
	Transformer	-3.810	-0.371	0.7115
	LSTM	-1.946	-0.211	0.8338
Shantou	GRU	-97.788	-5.432	<0.0001
	MLP	-102.531	-5.336	<0.0001
	NLinear	-78.307	-4.789	<0.0001
	TemporalConvNet	-62.721	-4.617	<0.0001
	Histogram Gradient Boosting	-6.094	-3.869	0.0003
	N-HiTS-small	-73.661	-3.801	0.0003
	Ridge	-11.362	-2.928	0.0047
	LSTM	-4.498	-2.328	0.0230
	Transformer	-4.110	-1.850	0.0689
	Extra Trees	-2.015	-1.846	0.0695
	SVR	-2.427	-1.316	0.1927
	Random Forest	-2.987	-1.027	0.3083
Shenzhen	TemporalConvNet	-176.911	-4.702	<0.0001
	GRU	-348.162	-4.345	<0.0001
	NLinear	-339.239	-4.230	0.0001
	Random Forest	-103.923	-4.211	0.0001
	MLP	-312.134	-4.206	0.0001
	N-HiTS-small	-261.003	-4.089	0.0001
	Extra Trees	-64.930	-4.025	0.0002
	Ridge	-65.335	-3.770	0.0004
	Transformer	-49.459	-3.766	0.0004
	LSTM	-46.588	-3.745	0.0004
	Histogram Gradient Boosting	-61.659	-3.273	0.0017
	SVR	-11.585	-2.378	0.0204
Dongguan	Extra Trees	-30.398	-4.927	<0.0001
	GRU	-206.977	-4.754	<0.0001
	Random Forest	-19.579	-4.661	<0.0001
	NLinear	-199.593	-4.124	0.0001
	MLP	-223.400	-3.911	0.0002
	TemporalConvNet	-42.980	-3.763	0.0004
	N-HiTS-small	-115.716	-3.407	0.0011
	Ridge	-30.987	-3.188	0.0022
	Histogram Gradient Boosting	-40.491	-3.134	0.0026
	LSTM	-22.365	-2.733	0.0081
	Transformer	-21.370	-2.711	0.0086
	SVR	-8.933	-1.984	0.0515

Table S6. Local baseline RMSE under the history-only and history-plus-weather conditions, with paired Diebold-Mariano p-values.

Station	Model	RMSE history-only	RMSE history-plus-weather	Change (%)	DM p
Huiyang	LSTM	16.61	16.76	+0.9	0.753
Huiyang	Extra Trees	11.48	12.76	+11.2	0.209
Huiyang	Random Forest	11.38	13.30	+16.9	0.186
Huiyang	SVR	12.54	14.84	+18.3	0.080
Huiyang	Ridge	12.27	14.68	+19.6	0.046
Huiyang	Histogram Gradient Boosting	11.39	13.68	+20.1	0.198
Huiyang	GRU	15.15	18.37	+21.3	0.139
Huiyang	NLinear	14.63	18.50	+26.4	0.025
Huiyang	Transformer	13.04	19.03	+45.9	0.048
Huiyang	MLP	13.09	20.13	+53.8	0.056
Huiyang	N-HiTS-small	13.01	20.62	+58.5	0.035
Huiyang	TemporalConvNet	11.94	19.17	+60.5	0.019
Shantou	Random Forest	3.73	3.24	-13.2	0.064
Shantou	Extra Trees	3.72	3.39	-9.0	0.142
Shantou	Histogram Gradient Boosting	4.10	3.76	-8.4	0.396
Shantou	Ridge	3.27	3.61	+10.5	0.128
Shantou	Transformer	5.03	6.50	+29.2	<0.001
Shantou	GRU	6.68	8.63	+29.3	<0.001
Shantou	SVR	3.20	4.15	+29.5	0.134
Shantou	LSTM	5.60	8.23	+47.0	<0.001
Shantou	TemporalConvNet	4.51	6.89	+52.7	<0.001
Shantou	N-HiTS-small	6.06	9.28	+53.0	0.007
Shantou	NLinear	5.21	8.37	+60.8	<0.001
Shantou	MLP	5.08	9.13	+79.8	<0.001
Shenzhen	LSTM	25.62	15.83	-38.2	<0.001
Shenzhen	SVR	22.54	18.70	-17.0	0.244
Shenzhen	GRU	20.68	19.13	-7.5	0.121
Shenzhen	NLinear	18.43	18.67	+1.3	0.657
Shenzhen	Histogram Gradient Boosting	14.21	15.22	+7.1	0.527
Shenzhen	Ridge	16.45	18.04	+9.6	0.141
Shenzhen	Random Forest	10.89	12.86	+18.1	0.092
Shenzhen	Extra Trees	12.29	14.98	+21.8	0.041
Shenzhen	Transformer	14.53	19.98	+37.5	0.006
Shenzhen	MLP	14.25	21.04	+47.6	0.002
Shenzhen	N-HiTS-small	15.45	24.71	+59.9	<0.001
Shenzhen	TemporalConvNet	9.40	20.97	+123.1	<0.001
Dongguan	Histogram Gradient Boosting	7.89	7.64	-3.1	0.828
Dongguan	Random Forest	6.62	7.33	+10.7	0.105
Dongguan	Ridge	7.42	8.43	+13.5	0.227
Dongguan	Extra Trees	6.77	7.78	+14.9	0.015
Dongguan	SVR	6.11	7.07	+15.8	0.113
Dongguan	Transformer	9.22	10.77	+16.8	0.013
Dongguan	GRU	9.77	12.74	+30.3	0.001
Dongguan	LSTM	8.94	12.05	+34.8	<0.001
Dongguan	NLinear	9.03	12.77	+41.4	<0.001
Dongguan	N-HiTS-small	9.25	13.83	+49.4	<0.001
Dongguan	TemporalConvNet	7.90	12.03	+52.2	<0.001
Dongguan	MLP	8.98	14.41	+60.5	<0.001

Note. The history-plus-weather condition contains the same variables as the history-only condition and adds 168 columns constructed solely from meteorology observed before the forecast origin. Change (%) = 100 × (history-plus-weather − history-only) / history-only. The p-value is from the horizon-averaged paired Diebold-Mariano test on squared error.

Table S7. Retraining ablations: capacity-matched versus bypassed implementations, and block-length sensitivity.

**Panel A. Capacity-matched vs bypassed**	**Active params**	**Δ (%)**	**95% CI (%)**	**Seeds worse**
Cross-station — masked to self-edges	246,757	+4.09	[−0.2, +6.9]	10/10
Cross-station — layer bypassed	229,473	+1.30	[−3.1, +3.8]	7/10
Threshold exposures — channels masked	246,757	+3.03	[−1.5, +6.0]	8/10
Threshold exposures — features removed	226,785	+3.56	[+0.2, +6.0]	10/10
History projection — bypassed	242,405	+9.48	[+3.0, +12.6]	10/10
Degree-day attention — input replaced by calendar week	246,757	+2.87	[−1.6, +5.9]	8/10
Relative attention bias — removed entirely	246,757	−1.12	[−3.4, +0.7]	4/10
Panel B. 95% CI by block length	4 weeks	8 weeks (primary)	12 weeks	
Remove history anchor	[+7.1, +21.3]	[+6.3, +19.8]	[+6.1, +18.5]	
Remove encoder branch	[+3.4, +16.9]	[+2.3, +18.5]	[+1.6, +20.6]	
No cross-station (self-edge)	[+0.5, +6.8]	[−0.2, +6.9]	[−0.2, +6.6]	
Mask threshold exposures	[−0.7, +6.0]	[−1.5, +6.0]	[−1.5, +5.8]	
Edge bias: distance only	[−0.9, +5.9]	[−1.5, +5.9]	[−1.5, +5.7]	
Optimization-sensitivity control	[−1.0, +5.9]	[−1.8, +5.9]	[−1.8, +5.6]	
Degree-day → calendar bias	[−1.0, +5.8]	[−1.6, +5.9]	[−1.7, +5.5]	
Fixed T_base at 8 °C	[−0.03, +0.01]	[−0.03, +0.01]	[−0.03, +0.01]	
Relative attention bias removed	[−3.4, +0.7]	[−3.4, +0.7]	[−3.5, +0.7]	

Note. Panel A pairs each intervention that can be implemented both ways. A masked implementation leaves every parameter in the forward pass and removes only the information; a bypassed implementation removes the module and its parameters as well. The two contrasts run in opposite directions, so matching capacity does not bias these comparisons in a consistent direction. Panel B repeats the primary eight-week block bootstrap at four and twelve weeks; no interval changes side of zero. The final pair of Panel A differs in kind from the others: both implementations keep every parameter, and they differ in how much of the mechanism is removed — the degree-day input replaced by a calendar week, or the relative attention bias dropped altogether. That pair is exploratory and sits outside the correction family of Table 4.

Table S8. Notation used in the TG-PestNet formulation.

Symbol	Meaning
S, L, H	Number of stations (4), lookback weeks (8), and forecast horizons (4)
V, G	Hourly weather variables (39) and physical variable groups (7)
X hour	Standardized hourly weather tensor with station, week, day, hour, and variable axes
T raw	Raw hourly temperature in degrees Celsius, used only for thermal-time (degree-day) accumulation
p s,l	Observed weekly DBM-infested plant percentage for station s at lookback week l, scaled to [0, 1]
u s,l, v s,l	Weekly mean 100-m eastward and northward wind components
theta ij, D ij	Bearing and normalized distance from source station i to receiving station j
ω k	ISO week number used for target horizon k in the frozen 52-week Fourier approximation (Eq. 9)
sigma, logit	Logistic function and its inverse
tau v,m, s v,m	Learned threshold and positive sharpness for variable v and threshold m (Eq. 2)
DD l, T_{base}	Weekly degree-days and learned base temperature (Supplementary Methods SM2.4)
Delta l,m	Thermal displacement between lookback weeks l and m (Eq. 4)
ρ_i→j	Projection of the weekly wind vector onto the i-to-j bearing; input to the learned edge-bias network (Eq. 6)
z_k, r_k, β	Bounded latent state and positive density-style adjustment (Eq. 10), and latent increment (Eq. 8; Supplementary Methods SM2.6)
ζ k	Embedding for forecast horizon k
a_s,k, w_s,k,l	History anchor and its convex lag weights (Eq. 11); the lag logits form one 4 × 4 × 8 tensor of 128 parameters
μ, ν, π	Interior mean, precision and endpoint-inflation weights of the auxiliary encoder-branch ZOIB distribution (Eq. 12)
m s,k^(w)	Mean of the auxiliary encoder-branch ZOIB mixture before history gating (Eq. 13)
g_s,k, ŷ_s,k	History–encoder gate and final bounded point forecast (Eq. 14); the gate logits form one 4 × 4 tensor of 16 parameters
y, Ω, λ_1, λ_2	Observed targets, valid-target mask and loss weights for the auxiliary likelihood and point errors (Eq. 15)
p̄, ā, m̄, ε_g	Endpoint-clipped quantities and clipping constant used before every logit operation; ε_g = 10^{-4} in the frozen implementation
r, k, q	Within-day hour index (1–24), forecast-horizon index (1–H), and Fourier harmonic index (1–F), respectively
K; q_1, …, q_K	Number and positions of the prespecified within-day order-statistic summaries; K = 5, q = (0.00, 0.25, 0.50, 0.75, 1.00), zero-based ranks (0, 6, 12, 17, 23) under round-half-to-even
ℓ_{g(v)}	Learned hour-of-day logits shared by variables in physical group g(v) (Supplementary Methods SM2.1)
κ	Fixed smooth-rectifier sharpness used in degree-day accumulation; κ = 4 (Supplementary Methods SM2.4)
dd_scale, w_scale, r_scale	Normalization or amplitude scales for thermal displacement, wind projection, and latent increments (Eqs. 4, 7 and 8); dd scale = 100 °C·day, w scale = 10 m s^{-1}, r scale = 2.0
F	Fourier order; F = 3 in the frozen configuration (Eq. 9)
f_d, f_g^week, E^week	Day-level feature vector, weekly representation of physical group g, and weekly station embedding (Eq. 3 and Supplementary Methods SM2.1); f d has 12 elements and f g^week has 24
ctx s, ζ k	Encoded station context and forecast-horizon embedding used by the decoder
h_s,l, Ẽ^week_s,l	Infestation-history embedding and the weekly station embedding after it is added, before the thermal-time encoder (Eq. 3a)
W_1, W_2, b_1, b_2	Weights and biases of the two-layer infestation-history projection, 64 × 2 and 64 × 64 with bias terms; 4,352 parameters (Eq. 3a)

Note. The history branch contains 128 station-horizon-lag logits (4 stations × 4 horizons × 8 lags) and 16 station-horizon gate logits. The lag logits are counted inside the decoder subtotal of Table S1, Panel B, and the gate logits are listed there as a separate tensor.

Table S9. Meteorological variable groups used by the hourly encoder.

Variable group	Count	Representative variables
Thermal	3	temperature_2m; apparent_temperature; wet_bulb_temperature_2m
Moisture	5	relative_humidity_2m; dew_point_2m; vapour_pressure_deficit
Precipitation	5	precipitation; rain; snowfall; snow_depth; weather_code
Radiation	6	shortwave_radiation; direct_radiation; diffuse_radiation
Wind	5	wind_speed_10m; wind_speed_100m; wind_direction_100m; gusts
Soil	8	soil temperature and soil moisture at four depths
Pressure/cloud	7	pressure_msl; surface_pressure; cloud cover; boundary-layer height

Note. Variable names are reproduced exactly as they appear in the source files, which use the Open-Meteo field-naming schema — hence temperature_2m, soil_moisture_0_to_7cm and the British spelling of vapour_pressure_deficit, which is retained here even though American English is used in the prose. That naming schema is why the platform can be mistaken for Open-Meteo; the files were obtained from HTQX, and the underlying product is ERA5 in both cases (Section 2.2). weather_code is categorical, although the frozen pipeline stored it as one numeric channel; its encoding and limitations are documented in SM1.3.

Table S10. Preprocessing corrections retrained under the protocol of Section 2.4.2 and reported outside the multiplicity family of Table 4.

Variant	Macro RMSE	Δ (%)	95% CI, 8-week	4-week	12-week
Exact day-of-year annual phase	6.403 ± 0.165	+0.10	[−0.41, +0.75]	[−0.41, +0.62]	[−0.36, +0.76]
Duplicate and degenerate channels removed	6.600 ± 0.148	+3.18	[−1.27, +6.35]	[−0.70, +6.28]	[−1.41, +6.38]

Note. Both variants use the frozen split, tuning seeds 37–39, final seeds 40–49 and no repeated hyperparameter search; the reference is the reported model at 6.397 ± 0.168. Δ and its intervals come from the paired block bootstrap of Section 2.4.1 with 2,000 replicates. Holm adjustment across these two comparisons gives 0.661 and 0.364, but the two test different objects and are reported for completeness rather than as a family. The reported model retains all 39 meteorological variables. The second row removes three hourly

channels — rain, which is numerically indistinguishable from precipitation over this window, and the two degenerate snow variables — and is therefore a different intervention from the optimization-sensitivity control of Table 4, which removes only the two snow variables and costs +2.91%. It is retrained here for the preprocessing question rather than as a yardstick.

Table S11. Field-surveillance protocol and availability of metadata in the dataset supplied to the authors.

Protocol dimension	Confirmed surveillance procedure	Available in weekly extract?	Remaining limitation
Survey frequency	Every 3–5 days	No record-level dates	Exact number of rounds per station-week unavailable
Fields	1–3 representative fields per round	No field identifiers	Exact weekly field count unavailable
Sampling design	Five points per field	No point-level records	Within-field spatial variation cannot be audited
Plants inspected	20 per point; 100 per field per round	Weekly percentage only	Exact weekly total unavailable
Weekly aggregation	Pooled numerator divided by pooled denominator	Final weekly percentage available	Contributing records unavailable
DBM diagnosis	Direct DBM observation or characteristic feeding signs	No plant-level diagnosis	Original classifications cannot be re-adjudicated
Insecticide use	No insecticides during the full crop-growing period	Confirmed by provider	Surrounding-field management unavailable
Longitudinal unit	Administrative monitoring area	Station identifier available	Physical fields could be replaced
Cultivar and crop stage	Recorded in local field logs	Not systematically retained	Cannot adjust for cultivar or phenology
Observer consistency	Same formal protocol throughout 2015–2022	Observer identifiers unavailable	Individual-observer continuity unknown

Note. Protocol details were confirmed in writing by the Department of Agriculture and Rural Affairs of Guangdong Province. “Available in weekly extract” refers to the aggregated weekly station-level dataset supplied to the authors, which is the only form of the response data available for this study.

Table S12. Year-ahead evaluation re-aggregated by season.

Season (target week)	Origins	TG-PestNet	TemporalConvNet, re-tuned	LastValue	Δ TG vs LastValue
December–February	60	10.030 ± 0.222	10.528	10.695	−6.2%
March–May	65	10.679 ± 0.340	10.556	12.773	−16.4%
June–August	62	8.311 ± 0.365	8.239	9.343	−11.0%
September–November	61	7.019 ± 0.148	7.489	7.936	−11.6%

Note. The same year-ahead forecasts as Table 5, Panel B, re-aggregated by the meteorological season of the target week and pooled over the four test years; no model was retrained. Macro RMSE in percentage points, computed per station and horizon within each season and averaged with equal weight, then averaged over seeds 40–49 (± SD across seeds).

Table S13. Near-zero-variance screening of the 39 hourly meteorological variables.

**Variable**	**Distinct**	**% at mode**	**Freq. ratio**	**% unique**	**NZV**	**Degenerate**
temperature_2m	358	1.0	1.03	0.1549	—	—
relative_humidity_2m	87	4.1	1.02	0.0376	—	—
dew_point_2m	424	1.5	1.02	0.1834	—	—
apparent_temperature	491	0.7	1.03	0.2124	—	—
precipitation	216	74.8	10.5	0.0934	—	—
rain	216	74.8	10.5	0.0934	—	—
snowfall	3	99.995	28,895	0.0013	yes	yes
snow_depth	1	100.000	∞	0.0004	yes	yes
weather_code	11	28.5	1.25	0.0048	—	—
pressure_msl	522	0.6	1.01	0.2258	—	—
surface_pressure	527	0.5	1.00	0.2280	—	—
cloud_cover	101	23.2	3.16	0.0437	—	—
cloud_cover_low	101	22.0	1.87	0.0437	—	—
cloud_cover_mid	101	22.1	3.80	0.0437	—	—
cloud_cover_high	101	43.2	5.87	0.0437	—	—
et0_fao_evapotranspiration	82	28.0	3.93	0.0355	—	—
vapour_pressure_deficit	39	14.0	1.19	0.0169	—	—
wind_speed_10m	162	3.2	1.04	0.0701	—	—
wind_speed_100m	235	2.0	1.00	0.1017	—	—
wind_direction_10m	360	1.2	1.45	0.1557	—	—
wind_direction_100m	361	0.7	1.04	0.1562	—	—
wind_gusts_10m	312	1.4	1.02	0.1350	—	—
soil_temperature_0_to_7cm	389	1.0	1.00	0.1683	—	—
soil_moisture_0_to_7cm	41	6.7	1.21	0.0177	—	—
shortwave_radiation	999	47.4	55.2	0.4322	yes	—
direct_radiation	851	51.7	34.9	0.3681	yes	—
diffuse_radiation	439	47.4	55.2	0.1899	yes	—
direct_normal_irradiance	928	51.7	171.1	0.4014	yes	—
global_tilted_irradiance	999	47.4	55.2	0.4322	yes	—
terrestrial_radiation	1,334	46.7	150.6	0.5771	yes	—
soil_temperature_7_to_28cm	268	1.2	1.01	0.1159	—	—
soil_temperature_28_to_100cm	174	2.1	1.07	0.0753	—	—
soil_temperature_100_to_255cm	106	2.6	1.04	0.0459	—	—
soil_moisture_7_to_28cm	33	6.2	1.06	0.0143	—	—
soil_moisture_28_to_100cm	40	6.6	1.16	0.0173	—	—
soil_moisture_100_to_255cm	18	9.2	1.02	0.0078	—	—
boundary_layer_height	396	0.8	1.03	0.1713	—	—
wet_bulb_temperature_2m	327	1.4	1.00	0.1415	—	—
total_column_integrated_water_vapour	759	0.3	1.02	0.3283	—	—

Note. Computed on the hourly record the reported model consumes: four stations over the common-calendar window of Sections 2.1 and 7 September 2015 to 10 April 2022, 231,168 station-hours. The caret-style rule is caret::nearZeroVar at its defaults. The frequency ratio is the count of the most common value divided by the count of the second most common, infinite when only one value occurs; per cent unique is the number of distinct values as a percentage of the 231,168 hours; a variable is flagged (NZV) when the ratio exceeds 19 and per cent unique falls below 10. The rule flags eight of the 39, but six of the eight are radiation variables whose modal value is zero for 46.7% to 51.7% of hours because half of every day is night: they carry the full diurnal cycle and are not degenerate, which is why the screening is reported rather than applied. Under the substantive criterion — constant over the window, or non-constant on fewer than 0.1% of hours and constant on validation and on test — exactly two of the 39 are degenerate, and both are snow variables. Snow depth is identically zero at every hour; its archived standardization scale is the unit scale that Section 2.2 assigns to any variable with a standard deviation below 10^{-6}, so it is exactly 0.0 after standardization and cannot influence any forecast. Snowfall is non-zero in 11 of the 231,168 hours (0.005%), all on 24 January 2016 and all inside the training era, and exactly constant on validation and on test. Its archived standardization scale is 8.50×10^{-4} at the selection stage and 7.34×10^{-4} at the final refit, so those eleven hours enter the network at approximately +165 σ and +191 σ; they fall in one calendar week and therefore perturb eight consecutive training origins, which is why Section 3.2 does not call the optimization-sensitivity control inert. One redundancy the rule does not detect is recorded here as well: precipitation and rain are numerically indistinguishable over this window (Pearson r = 1.000000; identical in 231,161 of the 231,168 hours). No screening was applied to the reported model, which retains all 39 variables; a variant retrained on the cleaned set is Supplementary Table S10.

Table S14. Chronological data partition and development folds.

A. Chronological split				
**Partition**	**Origins, n**	**Target rows, n**	**Start**	**End**
Training	195	780	2015-11-01	2019-07-21
Validation	66	264	2019-08-18	2020-11-15
Test	66	264	2020-12-13	2022-03-13
**B. Expanding folds for model selection**				
**Fold**	**Inner train, n**	**Inner validation, n**	**Outer fit, n**	**Outer evaluation, n**
1	126	32	161	31
2	161	31	195	31
3	195	31	229	32

Note. Origin counts are common to all four stations; target rows in Panel A are reported per station, with four forecast horizons per origin. Dates denote forecast origins, and associated target weeks may extend beyond the final date. A four-week embargo equal to the maximum forecast horizon separates training from validation and validation from test; this removes three intervening origin dates at each boundary. Panel B was used for the hyperparameter search of Section 2.4.1 and for tuning the baseline families; the reported TG-PestNet configuration was not selected from it.

Table S15. Audit index: the primary evidence for each structured component and the conclusion that evidence supports. Full statistics are in Tables S4, S7, S16, S17, S22, S23 and S28.

Component	Primary evidence	Supported conclusion
Base temperature T_base	Movement ≤ 0.006 °C from the 8.00 °C initialization at every seed; a twentyfold learning-rate sweep scatters it from 6.39 to 10.18 °C while accuracy falls 7.15% (Table S22)	Structural prior, not identified by these data
Degree-day pathway	Occlusion Δ = −0.0065%; calendar-week relative attention costs +2.87%, 95% CI [−1.6, +5.9]% (Table 4)	Not shown to be load-bearing; thermal time is not measurably better than calendar time here
Hour-of-day weights	Within approximately ±3% of uniform; entropy = $\log_2(24)$	No evidence of a learned diel window
Density-style term β	Moved 0.39% from initialization	No evidence of a learned demographic coefficient
Variable-group attention	Weights 0.133–0.155 around the uniform 1/7 (Table S4)	Routing weights, not validated importance
All weather	Occlusion of the frozen model +9.28%; retraining with meteorology zeroed +3.57%, 95% CI [−0.45, +8.98]% on the macro and +9.17%, [+1.7, +19.7]% at Huiyang (Table S25)	The frozen fitted model relies on meteorology. Under retraining, evidence is concentrated at Huiyang: its unadjusted interval excludes zero, but the result does not survive Holm adjustment across four stations (p = 0.072). No contribution is resolved at the other three stations
History anchor	Gate frozen at g = 1 costs +14.26%, 95% CI [+6.3, +19.8]%, Holm p < 0.001 (Table 4); the 16 gates are Table S16 and the 128 lag weights Figure S1	Load-bearing. The only ablation that survives multiplicity correction
Encoder branch	Gate frozen at g = 0 costs +9.91%, 95% CI [+2.3, +18.5]%, Holm p = 0.066	Contributes beyond the anchor, but the branch is not weather-only, so this is not by itself a meteorological result
Infestation-history projection	Bypassing the 4,352-parameter projection costs +9.48%, 95% CI [+3.0, +12.6]%, uncorrected p = 0.004	Interval excludes zero, but capacity falls with information, so the row is outside the correction family
Cross-station layer	Masking to self-edges costs +4.09%, [−0.2, +6.9]%; bypassing the layer entirely costs +1.30%, [−3.1, +3.8]%	Not shown to be load-bearing. Removing strictly more costs strictly less, so the two implementations bracket rather than estimate the effect
Station dependence	Occlusion +18.3% Huiyang, +9.8% Shantou, +3.6% Dongguan, −1.4% Shenzhen; retraining without meteorology +9.17%, −3.88%, −2.35% and +2.76% respectively	Station effects are heterogeneous. The macro figure is an aggregated RMSE percentage, not the mean of the station percentages
Attention versus perturbation attribution	Occlusion vs exact Shapley ρ = 0.79 (p = 0.05); group attention vs Shapley ρ = −0.14 (p = 0.78) (Table S4)	Attention weights cannot be reported as variable importance; only perturbation-based attribution supports ranking claims
Soft exposure thresholds	Masking the channels costs +3.03%, [−1.5, +6.0]%; removing the features costs +3.56%, [+0.2, +6.0]%; freezing all 24 at initialization changes RMSE by +0.06%; 4 of the 24 lie below the physical minimum (Table S17)	Not shown to be load-bearing and not claimed to be inert. A comparison against literature-derived fixed thresholds has not been run
Wind-informed edge bias	Wind group occlusion +0.87% (p = 0.007); distance-only edges cost +2.95%, [−1.5, +5.9]%; 42 of 120 sweep curves are not monotone increasing; the sign-constrained variant costs +2.55%, [−1.8, +5.4]% (Table S28)	The wind variables carry information; the edge bias that consumes them has no effect that is monotone, stable across seeds or separable from zero
Auxiliary ZOIB head	Randomized PIT departs from uniformity and the gate displaces the reported forecast from the ZOIB mean by 1.98 percentage points on average (Table S23)	Not a coherent final predictive distribution; no interval is reported from it

Table S16. Fitted history–encoder gate by station and forecast horizon.

Station	Horizon 1	Horizon 2	Horizon 3	Horizon 4	Mean
Huiyang	0.642 ± 0.022	0.695 ± 0.010	0.726 ± 0.011	0.717 ± 0.011	0.695
Dongguan	0.501 ± 0.015	0.527 ± 0.031	0.568 ± 0.013	0.548 ± 0.015	0.536
Shenzhen	0.442 ± 0.017	0.449 ± 0.021	0.472 ± 0.014	0.478 ± 0.020	0.460
Shantou	0.416 ± 0.020	0.419 ± 0.022	0.436 ± 0.017	0.452 ± 0.022	0.431

Note. Entries are the fitted gate of Equation (14) on the encoder-branch scale, so 0 is the pure history anchor and 1 the pure encoder branch, given as mean ± SD over the ten final seeds. The sixteen values are the complete 4 × 4 station-by-horizon tensor; the gate logits are initialized at zero, that is, at 0.5. Because the encoder branch also receives the observed history through the projection of Section 2.3.1, the gate measures the weight of the encoder pathway relative to the convex history anchor and not a pure weather-versus-history split. The mean encoder-branch weight at horizon 4 exceeds that at horizon 1 at every station, but the horizon profile is not monotone: it falls from horizon 3 to horizon 4 at Huiyang (0.726 to 0.717) and at Dongguan (0.568 to 0.548).

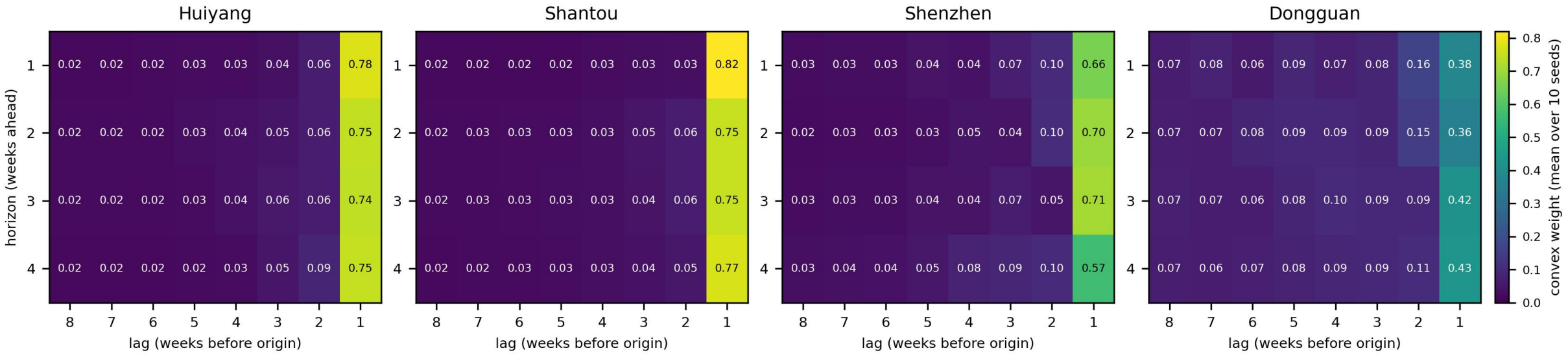

Figure S1. Fitted anchor lag weights, all 128 values. Each panel is one station; rows are forecast horizons and columns are the eight lookback weeks, most recent on the right. Cells give the convex weight averaged over the ten final seeds, so each row sums to one. Across-seed dispersion is small: the standard deviation of a single weight ranges from 0.0008 to 0.026. The profiles are station-specific, which is the direct summary of how each station and horizon use recent infestation history promised in Section 2.3.3. Writing the effective lag as the weighted mean number of weeks back, Huiyang and Shantou collapse toward persistence, placing 0.74–0.82 on the

most recent week for an effective lag of 1.6–1.8 weeks; Shenzhen is intermediate at 0.57–0.71 and 1.9–2.4 weeks; and Dongguan behaves like an eight-week smoother, placing 0.36–0.43 on the most recent week for an effective lag of 3.1–3.3 weeks. The corresponding profile entropies are 0.82–1.05 at Huiyang and Shantou, 1.15–1.49 at Shenzhen and 1.78–1.88 at Dongguan, against the ln 8 = 2.079 ceiling of a uniform profile. Averaged over the sixteen station–horizon cells, the total-variation distance from the initialization of Section 2.3.3 is 0.20. These are descriptive summaries of the fitted weights; the isolated forecasting contribution of the anchor is the retraining ablation of Section 3.2.

Table S17. Fitted exposure thresholds in physical units, all 24 values.

Variable	k	Fitted (SD units)	Fitted (physical)	Reachable	Hours ≥ threshold	Daily count range
Temperature (°C)	1	−1.5004 ±0.0059	14.371	Yes	90.1%	[0.01, 1.00]
	2	−0.5021 ±0.0050	20.018	Yes	67.3%	[0.00, 0.99]
	3	+0.5013 ±0.0049	25.694	Yes	38.3%	[0.00, 0.91]
	4	+1.5003 ±0.0062	31.346	Yes	2.7%	[0.00, 0.52]
Vapor-pressure deficit (kPa)	1	−1.5015 ±0.0049	−0.122	No	100.0%	[0.68, 1.00]
	2	−0.4985 ±0.0031	0.347	Yes	60.4%	[0.15, 1.00]
	3	+0.5006 ±0.0049	0.814	Yes	23.8%	[0.02, 0.98]
	4	+1.4985 ±0.0060	1.280	Yes	10.6%	[0.00, 0.84]
Precipitation (mm h⁻¹)	1	−1.5015 ±0.0050	−1.147	No	100.0%	[0.96, 1.00]
	2	−0.4994 ±0.0045	−0.232	No	100.0%	[0.65, 1.00]
	3	+0.4996 ±0.0047	0.680	Yes	7.9%	[0.14, 0.98]
	4	+1.4972 ±0.0051	1.591	Yes	3.7%	[0.01, 0.90]
Wind speed (m s⁻¹)	1	−1.4991 ±0.0067	0.744	Yes	97.7%	[0.51, 1.00]
	2	−0.4984 ±0.0057	2.202	Yes	63.2%	[0.10, 1.00]
	3	+0.4986 ±0.0065	3.654	Yes	28.2%	[0.01, 1.00]
	4	+1.4952 ±0.0058	5.105	Yes	7.8%	[0.00, 1.00]
Wind gusts (m s⁻¹)	1	−1.4982 ±0.0058	2.018	Yes	96.8%	[0.56, 1.00]
	2	−0.4966 ±0.0042	4.911	Yes	65.1%	[0.11, 1.00]
	3	+0.5019 ±0.0031	7.795	Yes	28.8%	[0.01, 1.00]
	4	+1.4992 ±0.0068	10.676	Yes	7.2%	[0.00, 1.00]
Shortwave radiation (W m⁻²)	1	−1.5000 ±0.0075	−198.065	No	100.0%	[0.89, 0.94]
	2	−0.5003 ±0.0054	51.302	Yes	43.7%	[0.41, 0.67]
	3	+0.5022 ±0.0052	301.372	Yes	26.1%	[0.06, 0.43]
	4	+1.4991 ±0.0048	550.043	Yes	13.1%	[0.01, 0.31]

Note. k indexes the four thresholds of each variable, initialized at −1.5, −0.5, +0.5 and +1.5 standardized units. Fitted values are means ± SD over the ten final seeds; the largest displacement of any threshold from its initialization is 0.005 standardized units. Physical values use the training-block standardization moments. A threshold is unreachable when it lies below the physical minimum of a non-negative variable, in which case every hour exceeds it and the exposure channel is saturated rather than informative. The sixth column is the share of the 231,168 station-hours in the joint study window at or above the fitted threshold. The final column gives the range of the daily exposure count of Equation (2), that is the 24-hour mean of the sigmoid, over the same hours; it shows that an unreachable threshold compresses the channel into a narrow high band rather than switching it off. The fitted sharpness barely moved either: across all 24 thresholds it lies between 0.406 and 0.408 standardized units against an initialization of 0.4065. Section 3.2 reports the forecasting effect of the exposure channels as a group.

Table S18. The 14 weekly weather variables of the flat block.

Weekly variable	Source column in the weekly panel	Type	Transform
temperature mean	temperature_2m_mean	state	identity
temperature min	temperature_2m_min	minimum	identity
temperature max	temperature_2m_max	maximum	identity
temperature range	temperature_2m_range	range	identity
relative humidity mean	relative_humidity_2m_mean	state	identity
vapor pressure deficit mean	vapour_pressure_deficit_mean	state	identity
precipitation total	precipitation_sum	accumulation	log1p_nonnegative
precipitation max hourly	precipitation_max_hourly	maximum	log1p_nonnegative
rainy hours or rainy days	precipitation_positive_hours	count	log1p_nonnegative
wind speed mean	wind_speed_10m_mean	state	identity
wind gust max	wind_gusts_10m_max	maximum	identity
shortwave radiation exposure	shortwave_radiation_exposure_proxy_sum	accumulation	identity
topsoil moisture mean	soil_moisture_0_to_7cm_mean	state	identity
surface pressure mean	surface_pressure_mean	state	identity

Note. Each weekly variable is a deterministic aggregate of one of the 39 hourly variables, computed on the weekly panel before any model sees it. The transform column is applied after aggregation; log1p_nonnegative is the natural logarithm of one plus the value, used for the three precipitation channels. The shortwave exposure proxy is the raw sum of hourly shortwave radiation over the week; no physical energy unit is claimed for it. Rain is excluded because it is numerically indistinguishable from precipitation in this record, and the two degenerate snow channels of Section 3.2 are excluded because they carry no information. This block enters the paired experiment of Section 2.4.1 as 112 lagged sequence columns and 56 window statistics.

Table S19. Baseline hyperparameter candidates and their selection scores.

Family	k	Configuration	Validation macro RMSE
Ridge	1	alpha=0.1	10.9171
	2	alpha=1.0	10.8621
	3	alpha=10.0	10.6486
	4	alpha=100.0	10.3773
Random Forest	1	max_depth=6, max_features=1.0, min_samples_leaf=2, n_estimators=250	10.4082
	2	max_depth=10, max_features=0.75, min_samples_leaf=3, n_estimators=350	10.4209
	3	max_depth=16, max_features=1.0, min_samples_leaf=4, n_estimators=450	10.4175
	4	max_depth=None, max_features=0.75, min_samples_leaf=5, n_estimators=500	10.3890
Extra Trees	1	max_depth=6, max_features=1.0, min_samples_leaf=2, n_estimators=250	10.2945
	2	max_depth=10, max_features=0.75, min_samples_leaf=3, n_estimators=350	10.2714
	3	max_depth=16, max_features=1.0, min_samples_leaf=4, n_estimators=450	10.3780
	4	max_depth=None, max_features=0.75, min_samples_leaf=5, n_estimators=500	10.2299
Hist. Gradient Boosting	1	learning_rate=0.08, max_iter=120, max_leaf_nodes=15, min_samples_leaf=10	10.8842
	2	learning_rate=0.05, max_iter=180, max_leaf_nodes=15, min_samples_leaf=14	10.8033
	3	learning_rate=0.03, max_iter=240, max_leaf_nodes=31, min_samples_leaf=16	10.9794
	4	learning_rate=0.02, max_iter=320, max_leaf_nodes=31, min_samples_leaf=20	10.8314
MLP	1	dropout=0.0, hidden_size=16, learning_rate=0.001, weight_decay=0.0001	11.6844
	2	dropout=0.05, hidden_size=32, learning_rate=0.0005, weight_decay=0.0001	10.7396
	3	dropout=0.1, hidden_size=64, learning_rate=0.0005, weight_decay=0.001	10.7725
	4	dropout=0.1, hidden_size=96, learning_rate=0.0003, weight_decay=0.001	11.0903
GRU	1	dropout=0.0, hidden_size=16, learning_rate=0.001, weight_decay=0.0001	11.5167
	2	dropout=0.05, hidden_size=24, learning_rate=0.0005, weight_decay=0.0001	11.9875
	3	dropout=0.1, hidden_size=32, learning_rate=0.0005, weight_decay=0.001	12.0783
	4	dropout=0.1, hidden_size=48, learning_rate=0.0003, weight_decay=0.001	11.3717
N-HiTS-small	1	dropout=0.0, hidden_size=16, learning_rate=0.001, weight_decay=0.0001	11.1931
	2	dropout=0.05, hidden_size=24, learning_rate=0.0005, weight_decay=0.0001	11.5446
	3	dropout=0.05, hidden_size=32, learning_rate=0.0005, weight_decay=0.001	10.7692
	4	dropout=0.1, hidden_size=48, learning_rate=0.0003, weight_decay=0.001	10.7471
NLinear	1	dropout=0.0, hidden_size=16, learning_rate=0.001, weight_decay=0.0001	11.9959
	2	dropout=0.0, hidden_size=24, learning_rate=0.0005, weight_decay=0.0001	12.0539
	3	dropout=0.05, hidden_size=32, learning_rate=0.0005, weight_decay=0.001	11.6987
	4	dropout=0.05, hidden_size=48, learning_rate=0.0003, weight_decay=0.001	11.4462
TemporalConvNet	1	dropout=0.0, hidden_size=16, learning_rate=0.001, weight_decay=0.0001	10.1289
	2	dropout=0.0, hidden_size=16, learning_rate=0.0005, weight_decay=0.0001	10.7507
	3	dropout=0.1, hidden_size=16, learning_rate=0.001, weight_decay=0.0001	9.9857
	4	dropout=0.1, hidden_size=16, learning_rate=0.0005, weight_decay=0.0001	10.2535
	5	dropout=0.0, hidden_size=24, learning_rate=0.001, weight_decay=0.0001	10.0177
	6	dropout=0.0, hidden_size=24, learning_rate=0.0005, weight_decay=0.0001	10.5842
	7	dropout=0.1, hidden_size=24, learning_rate=0.001, weight_decay=0.0001	9.9176
	8	dropout=0.1, hidden_size=24, learning_rate=0.0005, weight_decay=0.0001	10.4711
	9	dropout=0.0, hidden_size=32, learning_rate=0.001, weight_decay=0.0001	9.9138
	10	dropout=0.0, hidden_size=32, learning_rate=0.0005, weight_decay=0.0001	9.9810
	11	dropout=0.1, hidden_size=32, learning_rate=0.001, weight_decay=0.0001	9.8556
	12	dropout=0.1, hidden_size=32, learning_rate=0.0005, weight_decay=0.0001	9.8825
	13	dropout=0.0, hidden_size=48, learning_rate=0.001, weight_decay=0.0001	10.0050
	14	dropout=0.0, hidden_size=48, learning_rate=0.0005, weight_decay=0.0001	10.0272
	15	dropout=0.1, hidden_size=48, learning_rate=0.001, weight_decay=0.0001	9.8498
	16	dropout=0.1, hidden_size=48, learning_rate=0.0005, weight_decay=0.0001	9.8853
SVR	1	C=1.0, epsilon=0.05, gamma=scale	8.1826
	2	C=1.0, epsilon=0.05, gamma=0.01	7.8261
	3	C=1.0, epsilon=0.1, gamma=scale	8.1260
	4	C=1.0, epsilon=0.1, gamma=0.01	7.8242
	5	C=1.0, epsilon=0.2, gamma=scale	8.1530
	6	C=1.0, epsilon=0.2, gamma=0.01	7.7895
	7	C=10.0, epsilon=0.05, gamma=scale	9.7407
	8	C=10.0, epsilon=0.05, gamma=0.01	8.2789
	9	C=10.0, epsilon=0.1, gamma=scale	9.6838
	10	C=10.0, epsilon=0.1, gamma=0.01	8.2069
	11	C=10.0, epsilon=0.2, gamma=scale	10.2623
	12	C=10.0, epsilon=0.2, gamma=0.01	8.1640
	13	C=100.0, epsilon=0.05, gamma=scale	14.8762
	14	C=100.0, epsilon=0.05, gamma=0.01	9.9591
	15	C=100.0, epsilon=0.1, gamma=scale	14.5023

Family	k	Configuration	Validation macro RMSE
	16	C=100.0, epsilon=0.1, gamma=0.01	9.9972
	17	C=100.0, epsilon=0.2, gamma=scale	14.2904
	18	C=100.0, epsilon=0.2, gamma=0.01	10.5612
LSTM	1	dropout=0.0, hidden_size=16, learning_rate=0.0005, weight_decay=0.0001	7.9296
	2	dropout=0.0, hidden_size=16, learning_rate=0.001, weight_decay=0.0001	7.9250
	3	dropout=0.1, hidden_size=16, learning_rate=0.0005, weight_decay=0.0001	7.7583
	4	dropout=0.1, hidden_size=16, learning_rate=0.001, weight_decay=0.0001	7.7328
	5	dropout=0.2, hidden_size=16, learning_rate=0.0005, weight_decay=0.0001	7.6983
	6	dropout=0.2, hidden_size=16, learning_rate=0.001, weight_decay=0.0001	7.6961
	7	dropout=0.0, hidden_size=32, learning_rate=0.0005, weight_decay=0.0001	10.0362
	8	dropout=0.0, hidden_size=32, learning_rate=0.001, weight_decay=0.0001	10.1849
	9	dropout=0.1, hidden_size=32, learning_rate=0.0005, weight_decay=0.0001	10.0188
	10	dropout=0.1, hidden_size=32, learning_rate=0.001, weight_decay=0.0001	10.1744
	11	dropout=0.2, hidden_size=32, learning_rate=0.0005, weight_decay=0.0001	9.9860
	12	dropout=0.2, hidden_size=32, learning_rate=0.001, weight_decay=0.0001	10.1446
	13	dropout=0.0, hidden_size=64, learning_rate=0.0005, weight_decay=0.0001	10.1133
	14	dropout=0.0, hidden_size=64, learning_rate=0.001, weight_decay=0.0001	7.9291
	15	dropout=0.1, hidden_size=64, learning_rate=0.0005, weight_decay=0.0001	10.0938
	16	dropout=0.1, hidden_size=64, learning_rate=0.001, weight_decay=0.0001	7.8014
	17	dropout=0.2, hidden_size=64, learning_rate=0.0005, weight_decay=0.0001	10.0682
	18	dropout=0.2, hidden_size=64, learning_rate=0.001, weight_decay=0.0001	7.7315
Transformer	1	dropout=0.0, hidden_size=16, learning_rate=0.0005, weight_decay=0.0001	8.0601
	2	dropout=0.0, hidden_size=16, learning_rate=0.001, weight_decay=0.0001	7.9445
	3	dropout=0.1, hidden_size=16, learning_rate=0.0005, weight_decay=0.0001	7.9424
	4	dropout=0.1, hidden_size=16, learning_rate=0.001, weight_decay=0.0001	7.8211
	5	dropout=0.2, hidden_size=16, learning_rate=0.0005, weight_decay=0.0001	7.8813
	6	dropout=0.2, hidden_size=16, learning_rate=0.001, weight_decay=0.0001	7.7662
	7	dropout=0.0, hidden_size=32, learning_rate=0.0005, weight_decay=0.0001	8.0020
	8	dropout=0.0, hidden_size=32, learning_rate=0.001, weight_decay=0.0001	7.9787
	9	dropout=0.1, hidden_size=32, learning_rate=0.0005, weight_decay=0.0001	7.8280
	10	dropout=0.1, hidden_size=32, learning_rate=0.001, weight_decay=0.0001	7.7561
	11	dropout=0.2, hidden_size=32, learning_rate=0.0005, weight_decay=0.0001	7.7910
	12	dropout=0.2, hidden_size=32, learning_rate=0.001, weight_decay=0.0001	7.7169
	13	dropout=0.0, hidden_size=64, learning_rate=0.0005, weight_decay=0.0001	7.9838
	14	dropout=0.0, hidden_size=64, learning_rate=0.001, weight_decay=0.0001	7.9027
	15	dropout=0.1, hidden_size=64, learning_rate=0.0005, weight_decay=0.0001	7.9760
	16	dropout=0.1, hidden_size=64, learning_rate=0.001, weight_decay=0.0001	7.8158
	17	dropout=0.2, hidden_size=64, learning_rate=0.0005, weight_decay=0.0001	7.9350
	18	dropout=0.2, hidden_size=64, learning_rate=0.001, weight_decay=0.0001	7.7193

Note. k indexes the candidate grid of each family: 4 configurations for the first eight families, 16 for TemporalConvNet and 18 for SVR, LSTM and Transformer. Scores are validation macro RMSE for the history-only condition at global scope, the arm reported in Table 2; for the first nine families the score is the mean over the three development folds and three tuning seeds, and for SVR, LSTM and Transformer it is the single training-to-validation split on which those three were selected. The complete record, by fold, scope and feature condition, is released with the code. Selection never used the test split.

Table S20. Reported model by station and target-week mean temperature, the per-station composition of Supplementary Table S27, Panel A.

Station	Stratum	n	RMSE	MAE	Observed mean (%)
Huiyang	<20 °C	117	14.47	10.86	26.3
Huiyang	20–25 °C	51	9.12	7.49	27.8
Huiyang	25–30 °C	96	3.85	2.90	7.4
Shantou	<20 °C	128	2.67	1.90	20.1
Shantou	20–25 °C	44	3.04	2.39	20.7
Shantou	25–30 °C	92	3.49	2.59	17.6
Shenzhen	<20 °C	108	5.79	4.47	5.2
Shenzhen	20–25 °C	60	4.66	3.95	9.0
Shenzhen	25–30 °C	96	7.80	4.56	11.5
Dongguan	<20 °C	104	5.31	4.08	6.9
Dongguan	20–25 °C	64	6.51	4.45	11.3
Dongguan	25–30 °C	96	4.66	3.84	7.9

Note. RMSE and MAE in percentage points, computed per horizon, averaged with equal weight and then over the ten final seeds; the four station values of a stratum average to the corresponding cell of Supplementary Table S27, Panel A. n counts horizon-by-target-week forecasts per seed. Observed mean is the mean of the realized weekly DBM-infested plant percentage over the stratum. The cool-over-warm ordering of Supplementary Table S27 holds at Huiyang, is reversed at Shantou, and is not monotone at Shenzhen or Dongguan.

Table S21. Seed-level test RMSE for TG-PestNet and every stochastic comparator, seeds 40–49.

Station	Model	Test RMSE by seed, 40 to 49 (percentage points)	Mean ± SD
Huiyang	TG-PestNet (proposed)	10.788, 10.554, 11.206, 10.597, 10.404, 10.436, 10.158, 10.651, 11.309, 11.384	10.749 ± 0.417
Huiyang	TemporalConvNet	10.773, 10.818, 10.873, 10.707, 10.782, 10.849, 10.816, 10.911, 10.892, 10.916	10.834 ± 0.067
Huiyang	LSTM	10.913, 10.779, 10.736, 10.767, 10.750, 11.043, 10.966, 10.626, 10.954, 10.840	10.837 ± 0.129
Huiyang	Transformer	10.913, 10.915, 11.103, 10.805, 10.802, 11.110, 10.790, 10.930, 11.012, 10.823	10.920 ± 0.121
Huiyang	N-HiTS-small	11.082, 11.529, 11.202, 10.918, 11.184, 11.335, 11.000, 11.112, 11.132, 11.338	11.183 ± 0.179
Huiyang	Random Forest	11.365, 11.413, 11.401, 11.419, 11.374, 11.395, 11.361, 11.333, 11.356, 11.354	11.377 ± 0.028
Huiyang	Extra Trees	11.418, 11.426, 11.476, 11.473, 11.502, 11.469, 11.417, 11.499, 11.418, 11.458	11.456 ± 0.033
Huiyang	Raw-hourly TCN, weather off	11.790, 11.712, 11.698, 11.766, 11.791, 11.812, 11.805, 11.703, 11.703, 11.841	11.762 ± 0.053
Huiyang	NLinear	13.028, 11.052, 13.552, 11.327, 12.411, 11.609, 11.757, 11.607, 11.217, 11.713	11.927 ± 0.816
Huiyang	GRU	11.920, 11.474, 14.273, 12.925, 12.981, 13.051, 12.657, 12.065, 11.702, 11.772	12.482 ± 0.858
Huiyang	MLP	13.171, 13.172, 17.213, 12.610, 12.588, 11.967, 12.492, 12.820, 15.185, 13.003	13.422 ± 1.581
Shantou	TG-PestNet (proposed)	2.894, 3.186, 2.769, 2.983, 2.993, 3.382, 2.837, 2.933, 3.120, 3.224	3.032 ± 0.192
Shantou	TemporalConvNet	3.588, 3.792, 3.447, 4.023, 3.757, 3.707, 3.837, 3.864, 3.823, 3.740	3.758 ± 0.157
Shantou	LSTM	3.690, 3.724, 3.750, 3.997, 3.645, 3.694, 4.002, 3.454, 3.515, 3.570	3.704 ± 0.182
Shantou	Transformer	3.301, 3.693, 3.657, 3.687, 3.504, 3.774, 3.888, 3.747, 3.476, 3.610	3.634 ± 0.169
Shantou	N-HiTS-small	4.763, 5.581, 4.691, 4.803, 4.914, 5.032, 4.898, 4.702, 4.540, 4.369	4.829 ± 0.326
Shantou	Random Forest	3.703, 3.788, 3.737, 3.750, 3.692, 3.729, 3.706, 3.706, 3.740, 3.775	3.733 ± 0.032
Shantou	Extra Trees	3.328, 3.318, 3.348, 3.328, 3.361, 3.330, 3.299, 3.345, 3.312, 3.326	3.329 ± 0.018
Shantou	Raw-hourly TCN, weather off	4.863, 5.052, 4.984, 4.928, 4.708, 4.782, 4.653, 5.442, 4.627, 4.695	4.873 ± 0.247
Shantou	NLinear	6.720, 5.690, 9.070, 6.099, 7.421, 6.881, 5.255, 7.437, 5.771, 6.576	6.692 ± 1.109
Shantou	GRU	5.426, 7.602, 9.533, 6.412, 4.783, 5.878, 6.183, 5.253, 5.061, 5.738	6.187 ± 1.423
Shantou	MLP	6.338, 9.902, 12.656, 9.311, 7.032, 7.150, 7.942, 6.781, 10.294, 5.578	8.298 ± 2.190
Shenzhen	TG-PestNet (proposed)	6.182, 6.528, 6.419, 6.498, 6.155, 6.372, 6.338, 6.537, 6.433, 6.612	6.407 ± 0.150
Shenzhen	TemporalConvNet	8.348, 9.489, 9.193, 9.824, 9.695, 9.785, 9.095, 9.477, 9.293, 9.492	9.369 ± 0.432
Shenzhen	LSTM	9.427, 8.772, 8.246, 9.819, 9.538, 9.464, 9.722, 8.252, 9.230, 9.611	9.208 ± 0.582
Shenzhen	Transformer	9.473, 9.082, 9.601, 8.216, 8.982, 10.282, 9.799, 9.490, 9.110, 9.473	9.351 ± 0.551
Shenzhen	N-HiTS-small	11.471, 11.515, 12.044, 10.887, 12.370, 11.908, 12.469, 12.208, 10.305, 10.528	11.571 ± 0.772
Shenzhen	Random Forest	10.941, 10.709, 10.894, 10.865, 11.016, 10.833, 10.989, 10.915, 11.028, 10.740	10.893 ± 0.109
Shenzhen	Extra Trees	10.006, 9.914, 9.940, 9.968, 9.914, 9.870, 9.955, 9.884, 9.938, 9.843	9.923 ± 0.049
Shenzhen	Raw-hourly TCN, weather off	7.084, 7.016, 6.996, 7.041, 6.933, 7.057, 6.926, 7.119, 6.901, 6.878	6.995 ± 0.082
Shenzhen	NLinear	13.270, 15.655, 11.625, 13.762, 11.883, 20.073, 15.313, 17.060, 12.572, 13.779	14.499 ± 2.604
Shenzhen	GRU	11.682, 13.689, 11.133, 15.035, 15.211, 16.879, 16.311, 15.532, 13.699, 16.492	14.566 ± 1.980
Shenzhen	MLP	16.635, 12.102, 11.979, 13.186, 15.968, 17.754, 15.590, 14.431, 9.913, 15.217	14.277 ± 2.435
Dongguan	TG-PestNet (proposed)	5.063, 5.658, 5.165, 5.566, 5.162, 5.393, 5.123, 5.371, 5.482, 5.967	5.395 ± 0.284
Dongguan	TemporalConvNet	7.499, 7.721, 7.032, 7.334, 7.874, 7.413, 7.626, 7.187, 7.729, 7.284	7.470 ± 0.268
Dongguan	LSTM	7.174, 6.598, 7.063, 7.382, 6.924, 7.014, 7.313, 7.058, 7.227, 7.471	7.122 ± 0.252
Dongguan	Transformer	7.133, 6.655, 7.124, 6.827, 6.934, 7.434, 7.119, 7.382, 6.675, 7.214	7.050 ± 0.271
Dongguan	N-HiTS-small	8.863, 9.480, 8.687, 9.428, 9.604, 9.789, 8.857, 8.752, 9.277, 8.435	9.117 ± 0.454
Dongguan	Random Forest	6.630, 6.573, 6.688, 6.608, 6.550, 6.683, 6.597, 6.594, 6.642, 6.684	6.625 ± 0.049
Dongguan	Extra Trees	7.100, 7.137, 7.102, 7.033, 7.088, 7.077, 7.188, 7.138, 7.024, 7.127	7.101 ± 0.050
Dongguan	Raw-hourly TCN, weather off	5.741, 5.688, 5.799, 5.736, 5.652, 5.756, 5.640, 5.868, 5.643, 5.619	5.714 ± 0.080
Dongguan	NLinear	10.623, 14.121, 10.864, 11.634, 10.235, 14.926, 13.105, 15.972, 12.092, 12.939	12.651 ± 1.919
Dongguan	GRU	9.666, 11.479, 10.398, 13.076, 13.918, 14.331, 12.609, 13.714, 11.634, 15.031	12.586 ± 1.756
Dongguan	MLP	12.967, 12.000, 8.843, 12.484, 11.711, 12.612, 14.924, 13.270, 7.891, 14.929	12.163 ± 2.284

Note. Deterministic comparators — Ridge, SVR, Histogram Gradient Boosting — are fitted once and are omitted here; their single values are in Supplementary Table S2. Seed labels are paired across models under the registered same-label analysis convention; this convention does not imply identical stochastic trajectories across architectures. A row can therefore be read against another row of the same station as a reproducible descriptive pairing. At Huiyang TG-PestNet has the lower RMSE on six of the ten paired seeds against the TemporalConvNet, the comparison of Section 3.1 and Table 2, Panel B.

Table S22. Base temperature and accuracy under a sweep of the physical-parameter learning rate.

Arm	Physical-parameter learning rate	Refit epochs	T_base, mean ± SD (°C)	Across-seed range (°C)	Δ macro RMSE (%)	95% CI (%)
Reported model	0.1 × backbone	12	8.000 ± 0.003	8.00–8.01	—	—
Frozen base temperature	not trained	12	8.000, fixed	—	−0.01	[−0.03, +0.01]
Backbone rate	1 × backbone	12	8.000 ± 0.029	7.97–8.05	+0.08	[−0.11, +0.31]
Ten times backbone	10 × backbone	4	8.025 ± 0.094	7.92–8.23	+3.10	[−1.44, +5.98]
Twenty times backbone, long refit	20 × backbone	60	7.469 ± 1.078	6.39–10.18	+7.15	[+0.09, +16.64]

Note. Every arm follows the protocol of Section 2.4.2: the frozen split, tuning seeds 37–39, final seeds 40–49 and no repeated hyperparameter search. The refit epoch is chosen by the frozen policy — the argmin of the mean validation curve over the tuning seeds — which is why the ten-times arm stops at four epochs. Δ and its interval come from the paired block bootstrap of Section 2.4.1 with 2,000 replicates and eight-week blocks; these arms are reported outside the multiplicity family of Table 4. The last arm changes the learning rate and the refit length together and no reference-rate sixty-epoch control was run, so its accuracy cost is confounded; the across-seed range is the part of that row that speaks to identifiability.

Table S23. The auxiliary ZOIB head does not describe the reported forecast: interval coverage, probability integral transform, and the displacement introduced by the gate.

**A. Auxiliary ZOIB taken at face value as a predictive distribution for the observation**				
Nominal level	Empirical coverage	Gap (pp)	Mean width (pp)	Winkler score
0.50	56.7%	+6.7	9.08	17.48
0.80	79.3%	−0.7	17.36	26.64
0.90	86.1%	−3.9	22.48	33.74
0.95	91.0%	−4.0	27.56	41.30
Randomized probability integral transform: Kolmogorov–Smirnov D = 0.086 per seed on average, range 0.053 to 0.121; pooled D = 0.056, 95% CI [0.042, 0.148]; mean 0.483				
B. Displacement between the auxiliary distribution's mean and the reported point forecast				
Statistic	Mean	Median	95th percentile	Unit
Absolute displacement	1.98	1.42	5.52	percentage points

Note. Panel A evaluates the encoder-branch ZOIB of Section 2.3.3 as if it were the predictive distribution of the reported quantity, which the manuscript does not claim: coverage is the share of test observations falling inside the distribution's own central interval, and the probability integral transform is its CDF evaluated at the observation, tested against uniformity. The transform is randomized at the zero point mass, because the ordinary transform of a distribution with point masses is not uniform even under correct calibration; 370 of the 10,560 test rows are exact zeros and none is an exact one, and the randomization moves the pooled statistic by less than 0.0001. The statistic is given per seed, on 1,056 rows each, and pooled with a 95% interval from the moving-block bootstrap of Section 2.4.1 over the 66 forecast origins (block length 8, 2,000 replicates). No Kolmogorov–Smirnov p value is reported: the 10,560 pooled values are ten seeds × four stations × four overlapping forecast horizons × 66 origins and are not independent, so a nominal p value would be far too small. Panel B is the absolute difference between that distribution's mean and the gated point forecast of Equation (14), over all test rows. The calibration results document departures from nominal calibration. Separately, the gate displacement illustrates why the auxiliary distribution does not describe the reported point forecast; the absence of a coherent post-gate distribution follows from the model definition rather than from the calibration numbers themselves. Per-station and per-horizon displacements are in the released calibration output.

Table S24. Shuffled and noise controls on the flat weather block, by family and scope, at fifty draws.

Scope	Family	Control	H8	Real Δ	Control Δ	p (n=10)	p (n=50)	Not worse than real, of 50
local	Ridge	shuffled	9.856	+13.6%	+4.6%	1.000	0.961	48
		noise			+12.7%	0.455	0.628	31
	Extra Trees	shuffled	8.564	+16.0%	+3.2%	1.000	1.000	50
		noise			+7.5%	1.000	1.000	50
	TemporalConv Net	shuffled	8.450	+55.7%	+45.3%	0.909	0.824	41
		noise			+70.2%	0.091	0.078	3
global	Ridge	shuffled	7.967	+9.0%	+7.3%	0.727	0.824	41
		noise			+14.9%	0.091	0.020	0
	Extra Trees	shuffled	8.047	+1.7%	+0.1%	0.818	0.922	46
		noise			−0.7%	1.000	1.000	50
	TemporalConv Net	shuffled	7.790	+27.4%	+15.6%	1.000	1.000	50
		noise			+24.8%	0.818	0.863	43

Note. H8 is the history-only macro RMSE of that family and scope and Δ is the change from it; the control Δ is the mean over fifty draws. p = (b + 1)/(n + 1), one-sided, where b counts control draws whose penalty is no larger than the real block's, so the smallest attainable value is 0.09 at ten draws and 0.02 at fifty; the fifty draws include the original ten, and the n = 10 column is retained to show that the extension was applied to all twelve cells rather than to the two that were at the floor.

Table S25. Raw-hourly encoders with matched inputs, and three estimates of the contribution of meteorology.

Model or intervention	Macro RMSE	Δ vs TG-PestNet	95% CI	p
A. Input-matched architectures				
TG-PestNet	6.397 ± 0.168	—	—	—
History-only TemporalConvNet (reference)	7.858 ± 0.151	+22.8%	—	—
Raw-hourly TCN	8.085 ± 0.140	+26.4%	[+15.8, +35.4]%	<0.001
Raw-hourly TCN, hourly tensor zeroed	7.336 ± 0.091	+14.7%	[+3.9, +21.8]%	0.006
Raw-hourly Transformer	7.986 ± 0.189	+24.8%	[+14.3, +33.9]%	<0.001
Raw-hourly Transformer, hourly tensor zeroed	7.646 ± 0.236	+19.5%	[+6.9, +28.1]%	<0.001
Within architecture: TCN, with minus without	—	+10.2%	[+3.7, +19.0]%	0.006
Within architecture: Transformer, with minus without	—	+4.5%	[−2.4, +14.7]%	0.169
B. What each estimate of the meteorological contribution measures				
Occlusion of the frozen model (Section 3.2): how much the fitted model relies on meteorology	—	+9.28%	—	0.001
Encoder branch bypassed (Section 3.2): removes the branch and the history projection within it	7.031	+9.91%	[+2.3, +18.5]%	0.077
Retrained with meteorology zeroed: whether meteorology is necessary for the accuracy	6.625 ± 0.112	+3.57%	[−0.45, +8.98]%	0.064

Note. Δ, its interval and p come from the paired block bootstrap of Section 2.4.1 with 2,000 replicates and eight-week blocks. Holm correction is applied across the six comparisons of Panel A; no interval there changes side of zero at four or twelve weeks. The history-only TemporalConvNet is the primary comparator of Table 2, shown for reference and outside the correction family. Panel B is descriptive and its p values are uncorrected: the three rows are different interventions on different objects, not competing tests of one hypothesis. The retraining row is reported per station in Section 3.2: Huiyang +9.17% (95% CI [+1.7, +19.7]%, ten of ten seeds worse), Shenzhen +2.76% ([−3.3, +10.9]%), Dongguan −2.35% ([−8.5, +4.0]%) and Shantou −3.88% ([−14.1, +3.5]%); only the Huiyang unadjusted interval excludes zero (p = 0.018); the corresponding Holm-adjusted p value across the four stations is 0.072. One asymmetry favors the baselines: TG-PestNet's configuration was fixed from defaults and was not changed by its own search (Section 2.4.1), whereas each generic encoder's reported configuration is the winner of a 144-fit search.

Table S26. Archive coverage under the common calendar, and the four-arm comparison at Huiyang and Shantou.

Panel A. Archive coverage and training origins.

Station	Archive start	Archive weeks	Common-calendar weeks	Retained (%)	Training origins, common → full
Huiyang	2004-02-23	951	344	36.2	195 → 797
Shantou	2005-05-30	886	344	38.8	195 → 731
Shenzhen	2015-09-07	344	344	100.0	195 → 195
Dongguan	2015-09-07	349	344	98.6	195 → 195

Panel B. Test RMSE by arm, percentage points, mean ± SD over the ten final seeds.

Station	A, joint	A′, joint without exchange	B, single-station, common calendar	C, single-station, full archive
Huiyang	10.699 ± 0.262	11.113 ± 0.445	10.758 ± 0.216	11.363 ± 0.478
Shantou	3.048 ± 0.223	2.993 ± 0.180	3.061 ± 0.205	2.787 ± 0.109
Shenzhen	—	—	6.576 ± 0.300	6.576 ± 0.300

Panel C. Contrasts, Δ RMSE (%) with 95% confidence interval and Holm-adjusted p within the family of eight.

Contrast	Question	Huiyang	Shantou
C vs B	restoring early history in a single-station model	+5.63 [−1.5, +16.9], p = 0.71	−8.95 [−19.8, +2.4], p = 0.71
A vs C	reported joint model against a single-station model given the full archive	−5.84 [−12.5, −1.3], p = 0.14	+9.39 [−1.4, +21.9], p = 0.71
A vs A′	adding cross-station exchange	−3.72 [−8.8, +1.1], p = 0.71	+1.85 [−5.7, +12.6], p = 1.00
A′ vs B	joint training without exchange against single-station	+3.30 [−4.9, +13.7], p = 1.00	−2.22 [−12.5, +7.3], p = 1.00

Note. Δ = (RMSE of the first-named arm − RMSE of the second)/RMSE of the second; negative values favor the first-named arm. Panel B reports the RMSE of each arm at that station; Shenzhen has no history beyond the common calendar, so its B and C arms use identical data and are shown as a determinism check. Intervals and p values come from the paired block bootstrap of Section 2.4.1 with 2,000 replicates and eight-week blocks; Holm correction is applied within the eight station-by-contrast tests of Panel C, and the Shenzhen check is outside the family. The uncorrected p for A vs C at Huiyang is 0.018, and its interval excludes zero at eight- and twelve-week blocks but not at four. The four contrasts at a station are linked — A vs C is the composition of the other three — so the family is conservative. The configuration was selected at 195 joint origins and reused unchanged in every arm (Section 2.4.2).

Table S27. Test RMSE by thermal regime of the target week (percentage points; macro convention of Table 2; mean over seeds 40–49).

Panel A. Target-week mean temperature.

**Stratum**	**n**	**Reported model**	**Degree-day → calendar bias**	**Mask threshold exposures**	**Raw-hourly TemporalConvNet**	**LastValue**	**Δ calendar bias vs reported model (%) [95% CI]**
<20 °C	457	7.06	7.35	7.36	9.34	7.97	+4.07 [−3.1, +6.6]
20–25 °C	219	5.83	5.70	5.73	7.47	6.95	−2.24 [−8.3, +4.6]
25–30 °C	380	4.95	5.24	5.24	6.00	6.77	+5.90 [−6.4, +14.3]
>30 °C	0	—	—	—	—	—	—

Panel B. Target-week maximum hourly temperature.

**Stratum**	**n**	**Reported model**	**Degree-day → calendar bias**	**Mask threshold exposures**	**Raw-hourly TemporalConvNet**	**LastValue**	**Δ calendar bias vs reported model (%) [95% CI]**
<30 °C	584	6.67	6.90	6.91	8.85	7.68	+3.45 [−3.3, +6.0]
30–38 °C	472	5.65	5.73	5.74	6.73	7.14	+1.43 [−5.8, +8.5]
≥38 °C	0	—	—	—	—	—	—

Panel C. Target-week accumulated degree-days, training-era terciles.

**Stratum**	**n**	**Reported model**	**Degree-day → calendar bias**	**Mask threshold exposures**	**Raw-hourly TemporalConvNet**	**LastValue**	**Δ calendar bias vs reported model (%) [95% CI]**
Low	456	7.13	7.43	7.44	9.36	8.16	+4.12 [−3.2, +6.5]
Mid	248	5.49	5.40	5.42	7.32	6.56	−1.67 [−6.8, +4.5]
High	352	5.02	5.33	5.32	6.00	6.74	+6.14 [−4.8, +15.1]

Note. Strata are computed from the target week's realized meteorology, which no model received at forecast time; they are descriptive and carry no causal reading. n counts station-by-horizon target-week forecasts per seed, and the three panels partition the same 1,056 forecasts. Cut-points: mean and maximum temperature as listed; degree-days above 8 °C with tercile boundaries fixed on the training era within each station (Section 2.4.2). The 30–38 °C row of Panel B merges 35–38 °C, whose forecasts came from 16 distinct origins, below the 20-origin minimum, into 30–35 °C; it contributed 20 of the 472 forecasts. Comparators are the stored test predictions of Table 4 (two thermal variants), Supplementary Table S25 (raw-hourly encoder) and the LastValue anchor; nothing was retrained. Δ = (RMSE of the calendar-attention variant − RMSE of the reported model)/RMSE of the reported model, so positive values favor the reported model; the interval comes from the paired block bootstrap of Section 2.4.1 with 2,000 replicates and eight-week blocks, and no multiplicity correction is applied.

Table S28. Wind edge bias: counterfactual sweep of the ten frozen seeds, and the sign-constrained retrained variant.

Panel A. Sweep by ordered station pair, ten seeds per pair.

**Pair (i → j)**	**Distance (km)**	**Increasing / decreasing / non-monotone (of 10)**	**Mean violation fraction**	**Downwind − upwind Δ attention, mean ± SD**
Dongguan → Huiyang	74	6 / 2 / 2	0.250	+0.0007 ± 0.0017
Dongguan → Shantou	302	7 / 3 / 0	0.300	+0.0009 ± 0.0023
Dongguan → Shenzhen	61	6 / 2 / 2	0.235	+0.0011 ± 0.0028
Huiyang → Dongguan	74	7 / 3 / 0	0.300	+0.0006 ± 0.0026
Huiyang → Shantou	230	8 / 2 / 0	0.200	+0.0007 ± 0.0028
Huiyang → Shenzhen	64	6 / 3 / 1	0.330	+0.0012 ± 0.0051
Shantou → Dongguan	302	6 / 3 / 1	0.335	+0.0012 ± 0.0040
Shantou → Huiyang	230	7 / 3 / 0	0.300	+0.0016 ± 0.0040
Shantou → Shenzhen	283	6 / 3 / 1	0.302	+0.0019 ± 0.0041
Shenzhen → Dongguan	61	6 / 3 / 1	0.338	+0.0006 ± 0.0029
Shenzhen → Huiyang	64	7 / 2 / 1	0.287	+0.0013 ± 0.0042
Shenzhen → Shantou	283	6 / 3 / 1	0.342	+0.0001 ± 0.0022
All pairs	—	78 / 32 / 10	0.293	+0.0010 ± 0.0033

Panel B. Sign-constrained variant, with two rows of Table 4 repeated for reference.

**Variant**	**Macro RMSE**	**Δ (%)**	**Seeds worse**	**95% CI, 8-week**	**4-week**	**12-week**	**Boot p**	**Registered params**
Sign-constrained edge bias	6.560	+2.55	7/10	[−1.8, +5.4]	[−1.1, +5.5]	[−1.9, +5.3]	0.296	246,769
Edge bias: distance only (Table 4)	6.585	+2.95	8/10	[−1.5, +5.9]	[−0.9, +5.9]	[−1.5, +5.7]	0.235	246,757
Optimization-sensitivity control (Table 4)	6.583	+2.91	8/10	[−1.8, +5.9]	[−1.0, +5.9]	[−1.8, +5.6]	0.272	246,757

Note. Panel A: the sweep replaces the downwind projection of Equation (6) by 41 values between the training-era 5th and 95th percentiles of that pair while distance and every other input are held at their true values; the realized weight is recomputed from the queries and keys captured from the frozen checkpoint and averaged over the four heads and the eight lookback weeks of the first sixteen test origins (Section 2.4.2). Monotone increasing means strictly increasing at all 40 adjacent steps; the non-increasing class is defined as never strictly increasing, which is a weaker condition than strictly decreasing. Distances are great-circle values between the station coordinates of Table 1. The mean realized weight on these edges is 0.246, against 0.250 for a uniform weighting over the four stations. Panel B: the sign-constrained variant is trained under the protocol of Section 2.4.2 and reported outside the family of Table 4 because the constraint changes the capacity of the edge bias; the distance-only and optimization-sensitivity control rows are reproduced from Table 4 and Supplementary Table S7. Intervals come from the paired block bootstrap of Section 2.4.1 with 2,000 replicates. The parameter column of Panel B counts registered parameters: the sign-constrained edge bias has twelve free parameters, and the 260-parameter perceptron it replaces remains allocated but receives no gradient (Section 2.4.2).

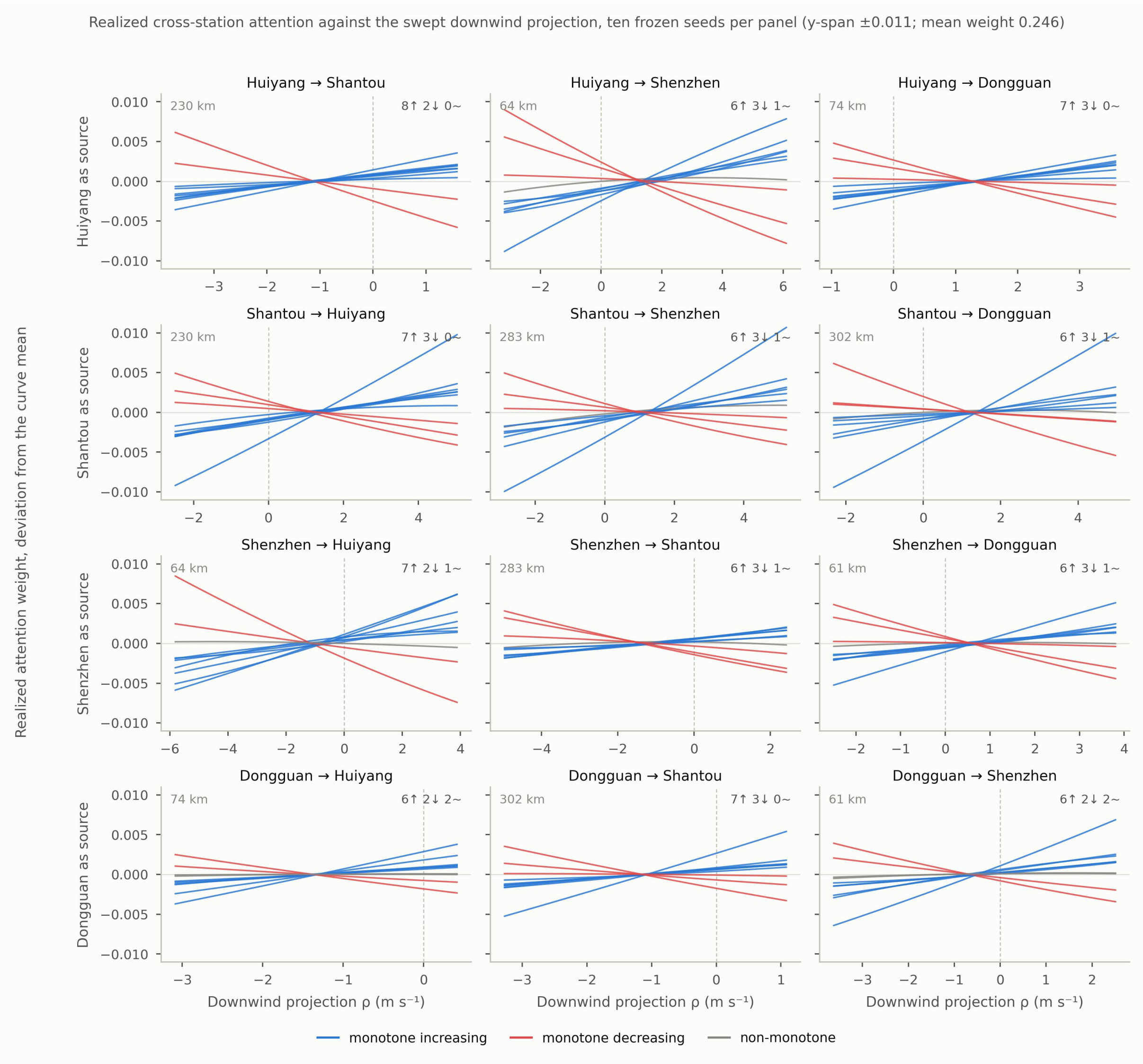

Realized cross-station attention against the swept downwind projection, ten frozen seeds per panel (y-span ±0.011; mean weight 0.246)
Realized attention weight, deviation from the curve mean
Huiyang as source
Shantou as source
Shenzhen as source
Dongguan as source
Huiyang → Shantou
230 km
8↑ 2↓ 0~
Huiyang → Shenzhen
64 km
6↑ 3↓ 1~
Huiyang → Dongguan
74 km
7↑ 3↓ 0~
Shantou → Huiyang
230 km
7↑ 3↓ 0~
Shantou → Shenzhen
283 km
6↑ 3↓ 1~
Shantou → Dongguan
302 km
6↑ 3↓ 1~
Shenzhen → Huiyang
64 km
7↑ 2↓ 1~
Shenzhen → Shantou
283 km
6↑ 3↓ 1~
Shenzhen → Dongguan
61 km
6↑ 3↓ 1~
Dongguan → Huiyang
74 km
6↑ 2↓ 2~
Dongguan → Shantou
302 km
7↑ 3↓ 0~
Dongguan → Shenzhen
61 km
6↑ 2↓ 2~
Downwind projection ρ (m s⁻¹)
monotone increasing
monotone decreasing
non-monotone

Figure S2. Counterfactual sweep of the wind edge bias: realized cross-station attention weight against the downwind projection of Equation (6), for each of the twelve ordered station pairs, with the ten frozen seeds drawn separately (Section 2.4.2). Each curve is centered on its own mean and all panels share a span of ±0.011, against a mean realized weight of 0.246 on these edges; curves are colored by whether the weight increases at all 40 sweep steps, never increases, or neither. Rows group the pairs by source station, the dashed line marks a zero projection, and each panel is annotated with the great-circle distance and the counts of the three classes.

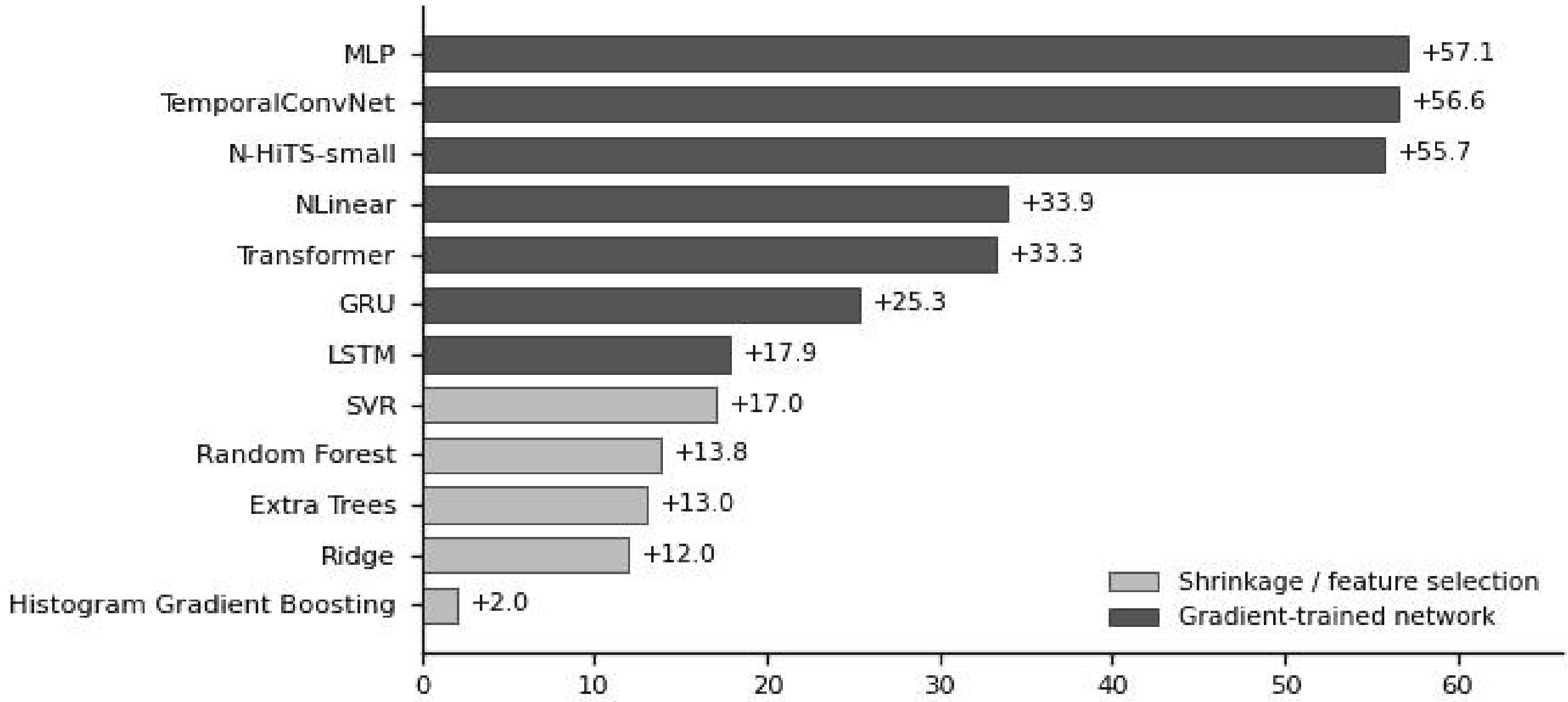

Figure S3. Median station-RMSE change after adding the 168-column weather block (history-plus-weather versus history-only), by model family. Families are ordered by the size of the penalty; the two bar groups distinguish models with shrinkage or built-in feature selection from gradient-trained networks receiving the flat 168-column block. The exact values are in Supplementary Table S3.

Note. Positive values indicate higher RMSE after weather was added. These results pertain to one flat distributed-lag encoding and do not establish that meteorology is intrinsically uninformative.
